# Supplementary material for: A Metabolomics Workflow for Analyzing Complex Biological Samples Using a Combined Method of Untargeted and Target-List Based Approaches
Source: Metabolites. 2020 Aug 25;10(9):342. doi: 10.3390/metabo10090342 (PMC7570008; doi:10.3390/metabo10090342)
Supplement: Supplementary file 1 [file metabolites-10-00342-s001.pdf]

Supporting Information

A metabolomics workflow for analyzing complex biological samples using a combined method of non-targeted and targeted approaches

Thomas Züllig 1†*, Martina Zandl-Lang 2†, Martin Trötzmüller 1, Jürgen Hartler 3, Barbara Plecko 2 and Harald C. Köfeler 1

1 Core Facility Mass Spectrometry, Medical University of Graz, Graz, Austria

2 Department of Paediatrics and Adolescent Medicine, Division of General Paediatrics, University Childrens' Hospital Graz, Medical University Graz, Graz, Austria.

3 Daten von Jürgen

* Correspondence: thomas.zuellig@medunigraz.at;

† Authors equally share first authorship

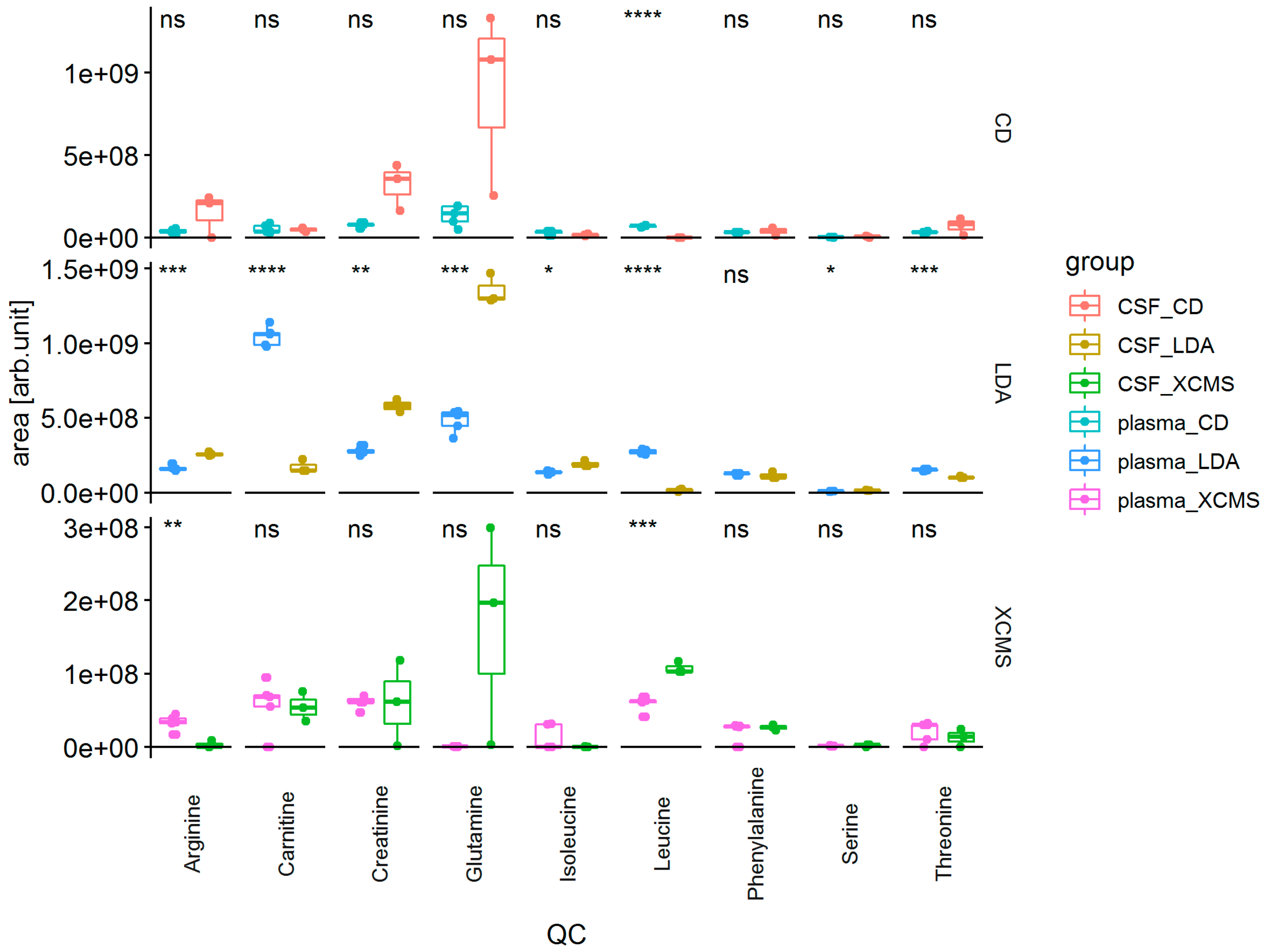

Figure S1: Increase of statistical power with LDA approach. Compounds with confident level 1 found in CSF and Plasma samples with all three data processing workflows: Compound Discoverer 3.0 (CD), XCMS and the combined approach with CD and Lipid Data analyzer 2.6 (LDA). ns: $p > 0.05$, *: $p \le 0.05$, **: $p \le 0.01$. ***: $p \le 0.001$, ****: $p \le 0.0001$

Table S1: List of detected compounds with the combined approach of Lipid Data Analyzer and Compound Discoverer 3.1. Compounds which were only detectable with CD or XCMS online are not listed. Confident level shows the confidence of the annotated compound (0-4), m/z are the detect mass to charge ratio of the compound, RT are the retention time.

Compounds	Confident level	m/z	RT	$Area_{QC1}$	$Area_{QC2}$	$Area_{QC3}$	$Area_{QC4}$	$Area_{QC5}$	Mean	SD	RSD	Matrix	Method
Adenosine	level1f1	268.1040	7.6	1.71E+05	3.05E+05	2.94E+05	1.64E+05	2.37E+05	2.34E+05	6.63E+04	28.32	plasma	LDA
Adenosine	level1f1	268.1043	7.6	4.52E+04	5.46E+04	6.30E+04	3.96E+04	4.92E+04	5.03E+04	8.98E+03	17.84	plasma	XCMS
Alanine	level1f2	90.0549	17.1	1.34E+08	1.41E+08	1.34E+08	1.28E+08	1.20E+08	1.32E+08	7.78E+06	5.91	plasma	LDA
Alanine	level1f2	90.0550	17.2	6.82E+04	9.27E+04	1.03E+05	1.75E+05	6.90E+04	1.02E+05	4.38E+04	43.12	plasma	XCMS
Alanine	level1f2	90.0551	16.7	2.56E+07	2.49E+07	2.24E+07	2.61E+07	2.07E+07	2.39E+07	2.30E+06	9.61	plasma	CD
Arginine	level1f3	175.1189	30.2	1.53E+08	1.47E+08	1.58E+08	1.64E+08	1.92E+08	1.63E+08	1.76E+07	10.85	plasma	LDA
Arginine	level1f3	175.1194	30.6	2.05E+07	5.35E+07	3.56E+07	4.50E+07	3.22E+07	3.74E+07	1.26E+07	33.66	plasma	CD
Arginine	level1f3	175.1194	30.4	1.73E+07	4.51E+07	3.39E+07	3.94E+07	3.25E+07	3.37E+07	1.04E+07	30.92	plasma	XCMS
Asparagine	level1f4	133.0607	22.0	5.37E+07	5.93E+07	5.94E+07	5.34E+07	4.56E+07	5.43E+07	5.65E+06	10.41	plasma	LDA
Asparagine	level1f4	133.0600	21.4	1.35E+07	9.85E+06	9.82E+06	1.30E+07	5.10E+06	1.03E+07	3.35E+06	32.69	plasma	CD
Aspartate	level1f5	134.0448	19.7	5.99E+05	2.19E+06	1.89E+06	1.22E+06	1.16E+06	1.41E+06	6.32E+05	44.79	plasma	LDA
Aspartate	level1f5	134.0450	19.6	1.34E+05	5.04E+05	2.16E+05	3.07E+05	2.21E+05	2.77E+05	1.41E+05	51.03	plasma	XCMS
Carnitine	level1f6	162.1125	9.3	1.06E+09	1.14E+09	1.07E+09	9.77E+08	9.90E+08	1.05E+09	6.43E+07	6.15	plasma	LDA
Carnitine	level1f6	162.1129	9.3	2.63E+07	8.74E+07	3.58E+07	3.56E+07	7.04E+07	5.11E+07	2.64E+07	51.62	plasma	CD
Carnitine	level1f6	162.1128	9.3	4.75E+05	6.87E+07	5.54E+07	7.05E+07	9.50E+07	5.80E+07	3.52E+07	60.69	plasma	XCMS
Choline	level1f7	104.1075	7.6	3.22E+08	2.50E+08	2.42E+08	2.72E+08	2.79E+08	2.73E+08	3.11E+07	11.40	plasma	LDA
Choline	level1f7	104.1074	7.6	5.35E+07	5.88E+07	6.18E+07	5.83E+07	6.80E+07	6.01E+07	5.34E+06	8.89	plasma	CD
Choline	level1f7	104.1073	7.5	6.67E+07	5.74E+07	5.49E+07	6.11E+07	6.33E+07	6.07E+07	4.68E+06	7.71	plasma	XCMS
Citrulline	level1f8	176.1029	22.2	4.54E+07	5.02E+07	4.84E+07	4.06E+07	4.04E+07	4.50E+07	4.46E+06	9.92	plasma	LDA
Citrulline	level1f8	176.1033	22.1	1.11E+07	1.37E+07	1.71E+07	1.48E+07	1.54E+07	1.44E+07	2.23E+06	15.45	plasma	CD
Creatinine	level1f9	114.0662	8.7	3.15E+08	2.69E+08	2.74E+08	2.48E+08	2.84E+08	2.78E+08	2.45E+07	8.80	plasma	LDA
Creatinine	level1f9	114.0664	8.7	9.20E+07	7.85E+07	7.97E+07	7.37E+07	5.57E+07	7.59E+07	1.32E+07	17.32	plasma	CD
Creatinine	level1f9	114.0664	8.6	7.02E+07	6.12E+07	6.56E+07	6.08E+07	4.71E+07	6.10E+07	8.65E+06	14.19	plasma	XCMS
Decanoylcarnitine	level1f10	316.2482	4.4	1.70E+07	1.11E+07	1.46E+07	2.29E+07	2.96E+07	1.90E+07	7.30E+06	38.36	plasma	LDA

Decanoylcarnitine	level1f10	316.2489	4.5	3.28E+06	2.77E+06	3.78E+06	6.66E+06	6.75E+06	4.65E+06	1.91E+06	41.16	plasma	CD
Decanoylcarnitine	level1f10	316.2487	4.5	3.25E+06	2.73E+06	3.37E+06	5.60E+06	6.37E+06	4.26E+06	1.62E+06	37.87	plasma	XCMS
Glutamic acid	level1f11	148.0604	19.3	5.05E+07	6.89E+07	7.97E+07	7.33E+07	6.32E+07	6.71E+07	1.11E+07	16.49	plasma	LDA
Glutamic acid	level1f11	148.0605	18.3	2.14E+07	1.73E+07	2.18E+07	1.90E+07	1.73E+07	1.94E+07	2.17E+06	11.22	plasma	CD
Glutamine	level1f12	147.0764	21.5	4.44E+08	5.19E+08	5.44E+08	5.35E+08	3.63E+08	4.81E+08	7.71E+07	16.03	plasma	LDA
Glutamine	level1f12	147.0768	20.4	1.88E+08	1.91E+08	4.88E+07	1.47E+08	9.83E+07	1.35E+08	6.10E+07	45.34	plasma	CD
Glutamine	level1f12	147.0765	21.7	7.50E+03	1.05E+06	9.89E+05	7.63E+05	8.51E+05	7.32E+05	4.20E+05	57.41	plasma	XCMS
Hippuric acid	level1f13	180.0655	2.3	1.89E+06	1.05E+06	1.96E+06	6.67E+05	1.18E+06	1.35E+06	5.58E+05	41.38	plasma	LDA
Histidine	level1f14	156.0767	30.5	9.85E+07	1.82E+08	1.06E+08	1.52E+08	1.27E+08	1.33E+08	3.41E+07	25.65	plasma	LDA
Histidine	level1f14	156.0770	30.6	3.80E+07	2.44E+07	3.48E+07	3.96E+07	3.53E+07	3.44E+07	5.93E+06	17.22	plasma	CD
Isoleucine	level1f15	132.1019	10.9	1.36E+08	1.40E+08	1.34E+08	1.22E+08	1.47E+08	1.36E+08	9.33E+06	6.88	plasma	LDA
Isoleucine	level1f15	132.1021	11.0	1.26E+07	3.78E+07	3.41E+07	3.01E+07	3.96E+07	3.08E+07	1.08E+07	35.16	plasma	CD
Isoleucine	level1f15	132.1020	11.1	1.23E+04	3.15E+07	6.40E+04	3.74E+05	3.10E+07	1.26E+07	1.70E+07	135.31	plasma	XCMS
Leucine	level1f16	132.1019	9.8	2.54E+08	2.69E+08	2.85E+08	2.91E+08	2.61E+08	2.72E+08	1.57E+07	5.76	plasma	LDA
Leucine	level1f16	132.1024	9.7	7.42E+07	7.05E+07	7.07E+07	6.40E+07	6.07E+07	6.80E+07	5.53E+06	8.13	plasma	CD
Leucine	level1f16	132.1022	9.7	4.16E+07	6.82E+07	6.32E+07	6.09E+07	6.28E+07	5.93E+07	1.02E+07	17.27	plasma	XCMS
Lysine	level1f17	147.1128	31.2	1.18E+08	1.67E+08	2.18E+08	1.63E+08	1.68E+08	1.67E+08	3.55E+07	21.30	plasma	LDA
Lysine	level1f17	147.1128	31.4	4.60E+07	1.58E+07	4.81E+07	4.84E+07	3.52E+07	3.87E+07	1.39E+07	35.88	plasma	CD
Methionine	level1f18	150.0583	13.3	9.15E+06	8.91E+06	8.67E+06	8.01E+06	7.95E+06	8.54E+06	5.35E+05	6.27	plasma	LDA
Methionine	level1f18	150.0586	13.2	2.22E+06	1.46E+06	2.29E+06	2.01E+06	2.03E+06	2.00E+06	3.25E+05	16.26	plasma	CD
Methioninesulfoxide	level1f19	166.0532	20.9	4.55E+06	6.14E+06	6.74E+06	6.05E+06	5.46E+06	5.79E+06	8.28E+05	14.31	plasma	LDA
Methioninesulfoxide	level1f19	166.0534	20.8	1.17E+06	1.50E+06	1.70E+06	1.52E+06	1.34E+06	1.45E+06	1.99E+05	13.76	plasma	CD
Methioninesulfoxide	level1f19	166.0533	20.8	1.09E+06	1.43E+06	1.65E+06	1.44E+06	1.31E+06	1.38E+06	2.06E+05	14.87	plasma	XCMS
Ornithine	level1f20	133.0971	31.9	3.99E+07	4.85E+07	3.58E+07	5.67E+07	5.07E+07	4.63E+07	8.42E+06	18.19	plasma	LDA
Ornithine	level1f20	133.0975	32.0	9.14E+06	1.85E+07	8.34E+06	1.62E+07	1.05E+07	1.25E+07	4.55E+06	36.23	plasma	CD
Ornithine	level1f20	133.0975	32.0	8.89E+06	1.29E+07	5.85E+06	1.20E+07	8.90E+06	9.71E+06	2.82E+06	29.06	plasma	XCMS
Palmitoylcarnitine	level1f21	400.3421	4.0	1.47E+07	9.96E+06	9.90E+06	1.30E+07	1.41E+07	1.23E+07	2.28E+06	18.49	plasma	LDA
Palmitoylcarnitine	level1f21	400.3425	4.0	4.01E+06	2.18E+06	2.79E+06	4.05E+06	3.86E+06	3.38E+06	8.46E+05	25.07	plasma	CD
Palmitoylcarnitine	level1f21	400.3425	4.0	3.93E+06	2.58E+06	2.61E+06	3.32E+06	3.94E+06	3.28E+06	6.72E+05	20.52	plasma	XCMS
Phenylalanine	level1f22	166.0862	10.2	1.29E+08	1.13E+08	1.25E+08	1.27E+08	1.29E+08	1.24E+08	6.62E+06	5.32	plasma	LDA

Phenylalanine	level1f22	166.0867	10.1	3.12E+07	2.75E+07	3.14E+07	3.08E+07	3.07E+07	3.03E+07	1.59E+06	5.26	plasma	CD
Phenylalanine	level1f22	166.0867	10.1	9.66E+04	2.73E+07	2.87E+07	2.83E+07	2.95E+07	2.28E+07	1.27E+07	55.78	plasma	XCMS
Proline	level1f23	116.0706	14.1	3.45E+08	3.68E+08	3.71E+08	3.69E+08	3.55E+08	3.62E+08	1.11E+07	3.08	plasma	LDA
Proline	level1f23	116.0709	14.0	8.75E+07	6.66E+07	8.80E+07	8.88E+07	7.04E+07	8.03E+07	1.08E+07	13.50	plasma	CD
Proline	level1f23	116.0709	14.0	7.99E+07	1.17E+06	8.18E+07	8.37E+07	5.81E+07	6.09E+07	3.50E+07	57.41	plasma	XCMS
Serine	level1f24	106.0499	20.9	1.05E+07	9.27E+06	1.09E+07	9.87E+06	7.34E+06	9.58E+06	1.40E+06	14.60	plasma	LDA
Serine	level1f24	106.0501	20.5	1.22E+06	1.26E+06	2.73E+06	2.41E+06	1.87E+06	1.90E+06	6.78E+05	35.71	plasma	CD
Serine	level1f24	106.0500	20.5	8.66E+05	2.12E+06	2.00E+06	2.22E+06	1.70E+06	1.78E+06	5.47E+05	30.75	plasma	XCMS
Taurine	level1f25	126.0219	14.6	2.17E+07	2.23E+07	2.21E+07	2.18E+07	2.14E+07	2.19E+07	3.63E+05	1.66	plasma	LDA
Taurine	level1f25	126.0223	14.4	8.97E+06	8.82E+06	8.89E+06	8.76E+06	7.22E+06	8.53E+06	7.38E+05	8.65	plasma	CD
Taurine	level1f25	126.0222	14.4	8.45E+04	5.28E+06	5.22E+06	5.25E+06	5.06E+06	4.18E+06	2.29E+06	54.81	plasma	XCMS
Threonine	level1f26	120.0655	18.3	1.51E+08	1.52E+08	1.59E+08	1.52E+08	1.42E+08	1.51E+08	5.82E+06	3.85	plasma	LDA
Threonine	level1f26	120.0658	18.3	3.60E+07	3.29E+07	3.90E+07	2.33E+07	3.30E+07	3.28E+07	5.89E+06	17.92	plasma	CD
Threonine	level1f26	120.0657	18.3	3.01E+07	1.04E+07	3.28E+07	3.46E+05	3.02E+07	2.08E+07	1.45E+07	69.97	plasma	XCMS
Tryptophan	level1f27	205.0971	10.2	4.40E+07	5.45E+07	5.16E+07	4.80E+07	5.04E+07	4.97E+07	3.95E+06	7.95	plasma	LDA
Tryptophan	level1f27	205.0975	10.3	1.17E+07	1.33E+07	1.34E+07	7.31E+04	1.27E+07	1.02E+07	5.71E+06	55.91	plasma	XCMS
Tyrosine	level1f28	182.0811	14.9	7.18E+07	6.68E+07	6.70E+07	6.94E+07	6.55E+07	6.81E+07	2.50E+06	3.67	plasma	LDA
Tyrosine	level1f28	182.0815	14.6	1.70E+07	1.63E+07	1.64E+07	1.65E+07	1.57E+07	1.64E+07	4.61E+05	2.81	plasma	XCMS
Valerylcarnitine	level1f29	246.1700	5.8	8.61E+06	9.33E+06	8.24E+06	1.09E+07	7.55E+06	8.93E+06	1.29E+06	14.41	plasma	LDA
Valerylcarnitine	level1f29	246.1703	5.8	2.26E+06	2.75E+06	2.18E+06	3.00E+06	2.00E+06	2.44E+06	4.19E+05	17.20	plasma	CD
Valerylcarnitine	level1f29	246.1703	5.8	2.32E+06	2.29E+06	2.10E+06	2.86E+06	1.92E+06	2.30E+06	3.54E+05	15.39	plasma	XCMS
Valine	level1f30	118.0862	10.9	6.52E+08	7.83E+08	7.56E+08	6.89E+08	6.70E+08	7.10E+08	5.67E+07	7.99	plasma	LDA
Valine	level1f30	118.0866	10.9	9.73E+07	1.55E+08	7.36E+07	1.60E+08	1.33E+08	1.24E+08	3.73E+07	30.13	plasma	CD
Valine	level1f30	118.0866	10.9	9.78E+07	1.48E+08	1.27E+08	1.18E+08	1.30E+08	1.24E+08	1.83E+07	14.76	plasma	XCMS
1-Methylpyrrolinium	level2f1	84.0807	31.4	7.80E+06	9.95E+06	9.68E+06	1.04E+07	9.52E+06	9.48E+06	1.00E+06	10.58	plasma	LDA
1-Methylpyrrolinium	level2f1	84.0808	31.4	1.91E+06	1.37E+06	2.31E+06	2.52E+06	1.74E+06	1.97E+06	4.59E+05	23.30	plasma	CD
1-Methylpyrrolinium	level2f1	84.0809	31.3	1.50E+06	1.78E+06	1.69E+06	2.08E+06	1.69E+06	1.74E+06	2.12E+05	12.17	plasma	XCMS
2-Aminoisobutyric acid	level2f2	104.0707	14.7	1.03E+07	1.29E+07	1.05E+07	9.67E+06	7.75E+06	1.02E+07	1.85E+06	18.12	plasma	LDA
2-Aminoisobutyric acid	level2f2	104.0708	14.7	2.50E+06	2.01E+06	2.28E+06	2.37E+06	2.02E+06	2.24E+06	2.12E+05	9.48	plasma	CD
2-Aminoisobutyric acid	level2f2	104.0708	14.7	2.46E+06	2.89E+06	2.47E+06	2.27E+06	1.86E+06	2.39E+06	3.72E+05	15.58	plasma	XCMS

4-Hydroxybenzaldehyde	level2f3	123.0441	14.6	3.24E+06	3.02E+06	3.29E+06	3.11E+06	3.29E+06	3.19E+06	1.23E+05	3.86	plasma	LDA
4-Hydroxybenzaldehyde	level2f3	123.0441	14.6	8.51E+05	7.30E+05	7.82E+05	7.42E+05	7.75E+05	7.76E+05	4.73E+04	6.10	plasma	CD
4-Hydroxybenzaldehyde	level2f3	123.0443	14.6	8.17E+05	7.59E+05	8.13E+05	7.65E+05	8.01E+05	7.91E+05	2.75E+04	3.48	plasma	XCMS
L-Pyroglutamic acid @RT:20.360	level2f4	130.0500	20.4	1.87E+08	2.12E+08	2.04E+08	1.85E+08	1.71E+08	1.92E+08	1.63E+07	8.50	plasma	LDA
L-Pyroglutamic acid @RT:20.360	level2f4	130.0501	20.4	4.68E+07	5.27E+07	4.46E+07	4.94E+07	1.76E+07	4.22E+07	1.41E+07	33.40	plasma	CD
L-Pyroglutamic acid @RT:20.360	level2f4	130.0502	20.4	3.85E+07	3.84E+07	4.13E+07	4.21E+07	3.17E+07	3.84E+07	4.11E+06	10.71	plasma	XCMS
Pipecolic acid @RT:10.502	level2f5	130.0865	10.5	8.68E+06	1.15E+07	1.11E+07	1.04E+07	9.37E+06	1.02E+07	1.17E+06	11.43	plasma	LDA
Pipecolic acid @RT:10.502	level2f5	130.0865	10.5	2.40E+06	2.81E+06	2.67E+06	2.42E+06	2.28E+06	2.52E+06	2.20E+05	8.74	plasma	CD
Pipecolic acid @RT:10.502	level2f5	130.0865	10.5	2.29E+06	2.84E+06	2.78E+06	2.61E+06	2.12E+06	2.53E+06	3.12E+05	12.32	plasma	XCMS
trans-4-Hydroxy-L-proline	level2f6	132.0657	17.8	1.92E+07	2.36E+07	1.93E+07	1.89E+07	1.41E+07	1.90E+07	3.36E+06	17.67	plasma	LDA
trans-4-Hydroxy-L-proline	level2f6	132.0657	17.6	5.23E+06	5.11E+06	5.07E+06	4.21E+06	4.18E+06	4.76E+06	5.18E+05	10.88	plasma	CD
trans-4-Hydroxy-L-proline	level2f6	132.0657	17.7	4.32E+06	3.17E+06	4.01E+06	3.23E+06	1.65E+06	3.28E+06	1.04E+06	31.60	plasma	XCMS
Creatine	level2f7	132.0772	14.0	1.39E+08	1.28E+08	1.32E+08	1.22E+08	1.18E+08	1.28E+08	8.17E+06	6.38	plasma	LDA
Creatine	level2f7	132.0770	14.0	3.96E+07	3.01E+07	3.93E+07	3.32E+07	3.24E+07	3.49E+07	4.32E+06	12.38	plasma	CD
Creatine	level2f7	132.0771	14.0	3.29E+07	1.92E+05	3.02E+07	2.87E+07	4.00E+05	1.85E+07	1.67E+07	90.19	plasma	XCMS
L-Norleucine @RT:11.081	level2f8	132.1022	11.0	1.33E+08	1.36E+08	1.40E+08	1.17E+08	1.27E+08	1.31E+08	8.90E+06	6.81	plasma	LDA
L-Norleucine @RT:11.081	level2f8	132.1021	11.0	1.26E+07	3.78E+07	3.41E+07	3.01E+07	3.96E+07	3.08E+07	1.08E+07	35.16	plasma	CD
L-Norleucine @RT:11.081	level2f8	132.1020	11.1	1.23E+04	3.15E+07	6.40E+04	3.74E+05	3.10E+07	1.26E+07	1.70E+07	135.31	plasma	XCMS
L-Norleucine @RT:11.950	level2f9	132.1024	12.0	1.03E+08	1.10E+08	1.08E+08	1.04E+08	1.04E+08	1.06E+08	3.13E+06	2.96	plasma	LDA
L-Norleucine @RT:11.950	level2f9	132.1021	11.9	2.60E+07	2.75E+07	2.61E+07	2.50E+07	2.55E+07	2.60E+07	9.45E+05	3.63	plasma	CD
L-Norleucine @RT:11.950	level2f9	132.1021	12.0	2.42E+07	2.36E+07	2.18E+07	2.24E+07	2.19E+07	2.28E+07	1.07E+06	4.69	plasma	XCMS
1-Aminocyclohexanecarboxylic acid	level2f10	144.1023	12.1	1.97E+07	1.56E+07	2.08E+07	1.31E+07	1.29E+07	1.64E+07	3.67E+06	22.41	plasma	LDA
1-Aminocyclohexanecarboxylic acid	level2f10	144.1021	12.1	1.78E+07	1.70E+07	1.66E+07	1.51E+07	1.63E+07	1.66E+07	1.01E+06	6.08	plasma	CD
1-Aminocyclohexanecarboxylic acid	level2f10	144.1020	12.1	4.69E+06	4.62E+06	4.78E+06	4.08E+06	4.23E+06	4.48E+06	3.09E+05	6.89	plasma	XCMS
Acetylcholine	level2f11	146.1179	7.6	2.73E+07	1.49E+07	1.48E+07	1.51E+07	1.67E+07	1.78E+07	5.40E+06	30.42	plasma	LDA
Acetylcholine	level2f11	146.1179	7.6	6.32E+06	4.00E+06	3.91E+06	3.97E+06	4.19E+06	4.48E+06	1.03E+06	23.12	plasma	CD
Acetylcholine	level2f11	146.1179	7.6	6.95E+06	3.69E+06	3.49E+06	3.25E+06	4.16E+06	4.31E+06	1.51E+06	35.13	plasma	XCMS
2-Hydroxycinnamic acid	level2f12	165.0547	14.6	6.41E+06	5.52E+06	5.79E+06	5.77E+06	5.72E+06	5.84E+06	3.33E+05	5.71	plasma	LDA
2-Hydroxycinnamic acid	level2f12	165.0548	14.6	2.05E+07	1.97E+07	1.94E+07	1.97E+07	1.53E+07	1.89E+07	2.06E+06	10.91	plasma	CD
2-Hydroxycinnamic acid	level2f12	165.0548	14.6	1.55E+06	1.31E+06	1.27E+06	1.39E+06	1.50E+04	1.11E+06	6.20E+05	55.95	plasma	XCMS

Methyl-DL-histidine	level2f13	170.0926	28.7	7.98E+06	9.62E+06	1.02E+07	7.84E+06	7.57E+06	8.64E+06	1.18E+06	13.63	plasma	LDA
Methyl-DL-histidine	level2f13	170.0925	28.6	2.39E+06	2.53E+06	2.65E+06	1.91E+06	1.63E+06	2.22E+06	4.35E+05	19.56	plasma	CD
Methyl-DL-histidine	level2f13	170.0926	28.5	1.80E+04	2.26E+06	2.21E+06	1.53E+06	1.78E+06	1.56E+06	9.13E+05	58.60	plasma	XCMS
N6,N6,N6-Trimethyl-L-lysine	level2f14	189.1601	28.4	6.22E+06	5.61E+06	4.59E+06	3.63E+06	3.06E+06	4.62E+06	1.32E+06	28.48	plasma	LDA
N6,N6,N6-Trimethyl-L-lysine	level2f14	189.1601	28.4	1.51E+06	1.38E+06	1.16E+06	8.63E+05	7.93E+05	1.14E+06	3.14E+05	27.51	plasma	CD
N6,N6,N6-Trimethyl-L-lysine	level2f14	189.1600	28.2	1.49E+06	1.24E+06	1.07E+06	7.15E+05	7.39E+05	1.05E+06	3.30E+05	31.50	plasma	XCMS
Acetyl-L-carnitine @RT:7.100	level2f15	204.1275	7.1	8.26E+08	7.06E+08	6.38E+08	7.54E+08	6.60E+08	7.17E+08	7.58E+07	10.58	plasma	LDA
Acetyl-L-carnitine @RT:7.100	level2f15	204.1190	7.1	1.45E+06	1.20E+06	1.15E+06	1.62E+06	1.28E+06	1.34E+06	1.92E+05	14.35	plasma	CD
Acetyl-L-carnitine @RT:7.100	level2f15	204.1275	7.1	1.64E+06	1.22E+06	1.12E+06	1.50E+06	1.17E+06	1.33E+06	2.28E+05	17.12	plasma	CD
Acetyl-L-carnitine @RT:7.100	level2f15	204.1235	7.0	1.94E+08	1.59E+08	1.54E+08	1.74E+08	1.54E+08	1.67E+08	1.72E+07	10.30	plasma	XCMS
Propionylcarnitine	level2f16	218.1392	6.5	5.49E+07	4.61E+07	4.34E+07	4.87E+07	3.45E+07	4.55E+07	7.46E+06	16.39	plasma	LDA
Propionylcarnitine	level2f16	218.1392	6.5	1.34E+07	1.22E+07	1.10E+07	1.23E+07	6.49E+06	1.11E+07	2.69E+06	24.33	plasma	CD
Propionylcarnitine	level2f16	218.1390	6.5	1.35E+07	1.18E+07	1.07E+07	1.16E+07	2.14E+04	9.51E+06	5.40E+06	56.74	plasma	XCMS
Lidocaine	level2f17	235.1808	5.1	2.38E+08	2.32E+08	2.81E+08	3.95E+08	4.46E+08	3.18E+08	9.68E+07	30.42	plasma	LDA
Lidocaine	level2f17	235.1808	5.1	6.63E+07	4.08E+07	7.56E+07	9.91E+07	1.20E+08	8.03E+07	3.04E+07	37.83	plasma	CD
Lidocaine	level2f17	235.1809	5.1	5.75E+07	5.40E+07	4.30E+07	9.43E+07	1.08E+08	7.13E+07	2.81E+07	39.33	plasma	XCMS
Carbamazepine @RT:2.135	level2f18	237.1029	2.1	2.97E+08	2.57E+08	2.44E+08	3.17E+08	3.04E+08	2.84E+08	3.15E+07	11.11	plasma	LDA
Carbamazepine @RT:2.135	level2f18	237.1026	2.1	7.72E+07	7.22E+07	6.76E+07	8.07E+07	7.69E+07	7.49E+07	5.07E+06	6.77	plasma	CD
Carbamazepine @RT:2.492	level2f19	237.1029	2.5	3.87E+08	3.51E+08	3.42E+08	3.72E+08	3.66E+08	3.64E+08	1.75E+07	4.80	plasma	LDA
Carbamazepine @RT:2.492	level2f19	237.1029	2.5	9.88E+07	9.29E+07	9.05E+07	1.06E+08	9.61E+07	9.69E+07	6.02E+06	6.21	plasma	CD
Carbamazepine @RT:2.492	level2f19	237.1027	2.5	5.82E+05	8.64E+07	8.74E+07	8.99E+07	8.92E+07	7.07E+07	3.92E+07	55.48	plasma	XCMS
L-Cystine	level2f20	241.0311	32.1	1.28E+07	2.54E+07	2.04E+07	3.14E+07	3.08E+07	2.42E+07	7.75E+06	32.11	plasma	LDA
L-Cystine	level2f20	241.0313	32.0	3.68E+06	6.71E+06	4.89E+06	7.61E+06	7.74E+06	6.12E+06	1.78E+06	29.04	plasma	CD
L-Cystine	level2f20	241.0315	32.0	2.55E+06	4.53E+06	3.27E+06	5.81E+06	5.68E+06	4.37E+06	1.44E+06	33.06	plasma	XCMS
Hexadecanamide	level2f21	256.2633	1.9	1.25E+08	1.83E+08	2.15E+08	2.39E+08	2.87E+08	2.10E+08	6.09E+07	29.02	plasma	LDA
Hexadecanamide	level2f21	256.2633	1.9	3.28E+07	4.79E+07	5.21E+07	1.09E+07	6.62E+07	4.20E+07	2.11E+07	50.14	plasma	CD
Hexadecanamide	level2f21	256.2624	2.0	7.72E+06	9.14E+04	1.17E+05	1.15E+07	1.17E+07	6.23E+06	5.81E+06	93.30	plasma	XCMS
N-Phenylacetylglutamine	level2f22	265.1187	3.5	6.21E+06	4.20E+06	3.62E+06	4.00E+06	1.76E+06	3.96E+06	1.59E+06	40.06	plasma	LDA
N-Phenylacetylglutamine	level2f22	265.1187	3.5	2.00E+06	1.13E+06	8.84E+05	8.04E+05	5.36E+05	1.07E+06	5.59E+05	52.23	plasma	CD
N-Phenylacetylglutamine	level2f22	265.1188	3.5	1.76E+06	1.23E+06	1.04E+06	9.49E+05	5.34E+05	1.10E+06	4.46E+05	40.47	plasma	XCMS

Pinolenic acid	level2f23	279.2319	1.9	5.91E+07	5.24E+07	4.40E+07	3.21E+07	3.19E+07	4.39E+07	1.21E+07	27.64	plasma	LDA
Pinolenic acid	level2f23	279.2321	1.9	9.01E+06	1.35E+07	8.51E+06	5.83E+06	6.67E+06	8.70E+06	2.97E+06	34.19	plasma	CD
Pinolenic acid	level2f23	279.2323	1.9	1.42E+07	1.18E+07	1.06E+07	7.61E+06	7.49E+06	1.03E+07	2.85E+06	27.58	plasma	XCMS
1-Methyladenosine	level2f24	282.1204	15.3	6.96E+06	9.09E+06	7.74E+06	7.10E+06	5.46E+06	7.27E+06	1.32E+06	18.09	plasma	LDA
1-Methyladenosine	level2f24	282.1206	15.3	1.26E+06	1.76E+06	1.96E+06	1.49E+06	1.37E+06	1.57E+06	2.86E+05	18.28	plasma	CD
1-Methyladenosine	level2f24	282.1202	15.3	1.69E+06	2.15E+06	1.82E+06	1.68E+06	1.36E+06	1.74E+06	2.85E+05	16.42	plasma	XCMS
Oleamide @RT:1.887	level2f25	282.2714	1.9	1.22E+09	1.53E+09	1.71E+09	2.08E+09	2.51E+09	1.81E+09	5.00E+08	27.66	plasma	LDA
Oleamide @RT:1.887	level2f25	282.2794	1.9	3.16E+08	3.93E+08	5.09E+08	5.20E+08	7.85E+08	5.05E+08	1.78E+08	35.30	plasma	CD
Oleamide @RT:1.887	level2f25	282.2800	1.9	2.60E+08	3.63E+08	3.79E+05	1.80E+06	2.21E+08	1.69E+08	1.62E+08	95.79	plasma	XCMS
Bis(4-ethylbenzylidene)sorbitol @RT:1.981	level2f26	415.2180	2.0	4.66E+08	4.38E+08	4.80E+08	5.17E+08	4.52E+08	4.71E+08	3.04E+07	6.46	plasma	LDA
Bis(4-ethylbenzylidene)sorbitol @RT:1.981	level2f26	415.2129	2.0	1.55E+08	1.57E+08	1.87E+08	2.04E+08	1.78E+08	1.76E+08	2.05E+07	11.63	plasma	CD
Bis(4-ethylbenzylidene)sorbitol @RT:1.981	level2f26	415.2135	2.0	9.56E+05	6.47E+05	5.32E+05	1.25E+08	5.65E+05	2.56E+07	5.58E+07	217.72	plasma	XCMS
3,4-Diaminopyridine	level3f1	110.0714	30.8	3.32E+07	4.51E+07	5.00E+07	4.91E+07	4.67E+07	4.48E+07	6.78E+06	15.11	plasma	LDA
3,4-Diaminopyridine	level3f1	110.0714	30.7	7.76E+06	1.40E+07	1.24E+07	1.25E+07	1.02E+07	1.14E+07	2.43E+06	21.41	plasma	CD
3,4-Diaminopyridine	level3f1	110.0715	30.5	4.66E+06	1.15E+07	8.78E+04	1.09E+07	6.50E+06	6.72E+06	4.70E+06	69.88	plasma	XCMS
Cyclohexylamine	level3f4	100.1123	7.2	2.40E+07	2.63E+07	2.64E+07	3.23E+07	3.07E+07	2.79E+07	3.41E+06	12.21	plasma	LDA
Cyclohexylamine	level3f4	100.1123	7.2	5.45E+06	6.30E+06	6.09E+06	7.66E+06	7.14E+06	6.53E+06	8.75E+05	13.39	plasma	CD
Cyclohexylamine	level3f4	100.1123	7.1	5.43E+06	6.18E+06	5.95E+06	7.55E+06	7.17E+06	6.46E+06	8.79E+05	13.61	plasma	XCMS
Piperidine @RT:11.950	level3f5	86.0965	11.9	9.00E+06	8.30E+06	8.10E+06	8.54E+06	7.55E+06	8.30E+06	5.38E+05	6.49	plasma	LDA
Piperidine @RT:11.950	level3f5	86.0965	12.0	2.56E+06	2.25E+06	2.11E+06	2.14E+06	2.04E+06	2.22E+06	2.05E+05	9.25	plasma	CD
Piperidine @RT:11.950	level3f5	86.0964	12.0	2.21E+06	2.17E+06	2.02E+06	2.08E+06	1.98E+06	2.09E+06	9.85E+04	4.70	plasma	XCMS
1-Nitrosopiperidine	level3f6	115.0870	32.0	7.87E+06	1.10E+07	7.66E+06	9.08E+06	7.56E+06	8.63E+06	1.44E+06	16.73	plasma	LDA
1-Nitrosopiperidine	level3f6	115.0869	32.0	1.97E+06	2.42E+06	7.96E+05	2.38E+06	1.66E+06	1.85E+06	6.64E+05	35.98	plasma	CD
1-Nitrosopiperidine	level3f6	115.0868	31.9	1.55E+06	2.33E+06	1.54E+06	2.13E+06	1.55E+06	1.82E+06	3.82E+05	21.02	plasma	XCMS
2-Aminoacetophenone;O-Acetylaniline	level3f7	136.0758	14.6	1.50E+07	1.28E+07	1.43E+07	1.35E+07	1.38E+07	1.39E+07	8.14E+05	5.87	plasma	LDA
2-Aminoacetophenone;O-Acetylaniline	level3f7	136.0758	14.6	7.09E+06	6.22E+06	6.59E+06	6.42E+06	5.84E+06	6.43E+06	4.62E+05	7.19	plasma	CD
2-Aminoacetophenone;O-Acetylaniline	level3f7	136.0760	14.6	3.58E+06	3.08E+06	3.44E+06	3.20E+06	2.71E+06	3.20E+06	3.35E+05	10.46	plasma	XCMS

3-Aminobenzamide	level3f8	137.0711	9.0	1.19E+07	1.22E+07	1.19E+07	1.19E+07	1.06E+07	1.17E+07	6.39E+05	5.46	plasma	LDA
3-Aminobenzamide	level3f8	137.0711	9.0	2.67E+06	3.00E+06	2.87E+06	2.87E+06	2.65E+06	2.81E+06	1.49E+05	5.31	plasma	CD
3-Aminobenzamide	level3f8	137.0711	8.9	3.97E+04	2.60E+04	2.82E+06	2.81E+06	2.58E+06	1.66E+06	1.48E+06	89.66	plasma	XCMS
allopurinol @RT:6.491	level3f10	137.0461	6.5	2.14E+08	2.22E+08	2.10E+08	2.43E+08	1.83E+08	2.14E+08	2.18E+07	10.19	plasma	LDA
allopurinol @RT:6.491	level3f10	137.0460	6.5	5.30E+07	4.69E+07	5.29E+07	3.95E+07	3.75E+07	4.59E+07	7.26E+06	15.81	plasma	CD
allopurinol @RT:6.491	level3f10	137.0460	6.4	4.97E+07	5.21E+07	5.06E+07	5.73E+07	4.21E+07	5.04E+07	5.48E+06	10.87	plasma	XCMS
Coumarone	level3f11	119.0491	14.7	6.29E+06	5.37E+06	5.76E+06	5.52E+06	5.96E+06	5.78E+06	3.64E+05	6.30	plasma	LDA
Coumarone	level3f11	119.0491	14.6	1.57E+06	1.26E+06	1.38E+06	1.32E+06	1.06E+06	1.32E+06	1.87E+05	14.16	plasma	CD
Coumarone	level3f11	119.0494	14.6	1.49E+06	1.28E+06	1.39E+06	1.29E+06	1.45E+06	1.38E+06	9.40E+04	6.81	plasma	XCMS
Dihydrothymine	level3f12	129.0661	14.7	1.55E+07	1.35E+07	1.33E+07	1.25E+07	1.14E+07	1.32E+07	1.51E+06	11.37	plasma	LDA
Dihydrothymine	level3f12	129.0661	14.7	3.86E+06	3.29E+06	2.60E+06	2.82E+06	2.08E+06	2.93E+06	6.80E+05	23.22	plasma	CD
Dihydrothymine	level3f12	129.0662	14.7	3.65E+06	3.21E+06	3.03E+06	2.96E+06	2.57E+06	3.08E+06	3.93E+05	12.74	plasma	XCMS
Indole;1-Benzazole @RT:10.217	level3f13	118.0653	10.3	3.25E+07	3.38E+07	3.22E+07	3.17E+07	3.37E+07	3.28E+07	9.10E+05	2.78	plasma	LDA
Indole;1-Benzazole @RT:10.217	level3f13	118.0655	10.2	9.12E+06	9.70E+06	6.12E+06	4.83E+06	9.62E+06	7.88E+06	2.25E+06	28.58	plasma	CD
Indole;1-Benzazole @RT:10.217	level3f13	118.0654	10.2	6.41E+06	8.05E+06	6.36E+06	7.39E+06	7.86E+06	7.21E+06	7.97E+05	11.04	plasma	XCMS
Indole;1-Benzazole @RT:14.613	level3f14	118.0652	14.6	5.40E+06	5.18E+06	4.26E+06	5.09E+06	4.93E+06	4.97E+06	4.32E+05	8.68	plasma	LDA
Indole;1-Benzazole @RT:14.613	level3f14	118.0652	14.6	1.91E+06	1.64E+06	1.78E+06	1.72E+06	1.53E+06	1.72E+06	1.41E+05	8.24	plasma	CD
Indole;1-Benzazole @RT:14.613	level3f14	118.0654	14.6	1.86E+06	1.63E+06	1.52E+06	1.70E+06	1.40E+06	1.62E+06	1.75E+05	10.78	plasma	XCMS
Isoquinoline	level3f15	130.0658	10.3	6.60E+06	7.37E+06	6.88E+06	6.19E+06	6.73E+06	6.76E+06	4.29E+05	6.34	plasma	LDA
Isoquinoline	level3f15	130.0658	10.3	1.62E+06	1.83E+06	1.66E+06	1.18E+06	1.56E+06	1.57E+06	2.40E+05	15.27	plasma	CD
Isoquinoline	level3f15	130.0654	10.3	1.76E+06	1.83E+06	1.78E+06	1.38E+04	1.73E+06	1.42E+06	7.89E+05	55.43	plasma	XCMS
Naphthalen-2-amine	level3f16	144.0815	10.2	1.62E+07	1.64E+07	1.38E+07	1.38E+07	1.02E+07	1.40E+07	2.51E+06	17.85	plasma	LDA
Naphthalen-2-amine	level3f16	144.0814	10.2	2.07E+06	3.08E+06	3.05E+06	2.20E+06	2.41E+06	2.56E+06	4.76E+05	18.59	plasma	CD
Naphthalen-2-amine	level3f16	144.0810	10.3	2.04E+06	3.43E+06	3.13E+06	3.65E+05	2.95E+04	1.80E+06	1.56E+06	86.44	plasma	XCMS
Stachydrine @RT:10.375	level3f17	144.1015	10.5	2.70E+08	2.87E+08	2.83E+08	2.77E+08	2.56E+08	2.75E+08	1.22E+07	4.45	plasma	LDA
Stachydrine @RT:10.375	level3f17	144.1023	10.4	7.05E+07	7.25E+07	6.78E+07	6.98E+07	6.54E+07	6.92E+07	2.72E+06	3.93	plasma	CD
Stachydrine @RT:10.375	level3f17	144.1023	10.4	5.80E+07	6.66E+07	6.53E+07	6.35E+07	5.99E+07	6.27E+07	3.65E+06	5.83	plasma	XCMS
Urea	level3f18	121.0724	4.4	5.79E+07	3.22E+07	3.66E+07	6.30E+07	5.01E+07	4.80E+07	1.33E+07	27.71	plasma	LDA
Urea	level3f18	121.0724	4.4	2.57E+07	1.47E+07	1.48E+07	2.79E+07	1.74E+07	2.01E+07	6.26E+06	31.11	plasma	CD
Urea	level3f18	121.0723	4.4	1.35E+07	5.54E+04	8.54E+06	1.44E+07	1.64E+05	7.34E+06	6.97E+06	94.97	plasma	XCMS

(E)-4-hydroxyphenylacetaldehydeoxime	level3f19	152.0709	9.0	2.75E+07	4.95E+06	3.25E+07	3.58E+06	2.73E+07	1.92E+07	1.38E+07	71.83	plasma	LDA
(E)-4-hydroxyphenylacetaldehydeoxime	level3f19	152.0708	9.1	7.06E+06	8.57E+06	8.23E+06	7.53E+06	6.54E+06	7.58E+06	8.30E+05	10.94	plasma	CD
(E)-4-hydroxyphenylacetaldehydeoxime	level3f19	152.0708	9.1	5.76E+06	7.54E+06	7.52E+06	7.49E+06	6.32E+06	6.92E+06	8.33E+05	12.04	plasma	XCMS
1 5-Anhydro-D-mannitol	level3f20	182.1024	3.0	4.18E+06	3.60E+06	9.43E+05	2.76E+06	4.00E+06	3.10E+06	1.32E+06	42.69	plasma	LDA
1 5-Anhydro-D-mannitol	level3f20	182.1025	3.0	8.00E+05	7.84E+05	5.42E+05	5.87E+05	5.60E+05	6.54E+05	1.26E+05	19.32	plasma	XCMS
1 5-Anhydro-D-mannitol	level3f20	182.1024	3.0	1.53E+06	1.65E+06	1.18E+06	1.22E+06	6.20E+05	1.24E+06	4.00E+05	32.23	plasma	CD
1 7-Dimethylxanthine(paraxanthine)	level3f21	181.0720	3.0	2.96E+07	5.93E+07	5.11E+07	5.55E+07	5.82E+07	5.07E+07	1.22E+07	24.12	plasma	LDA
1 7-Dimethylxanthine(paraxanthine)	level3f21	181.0720	2.9	5.51E+06	1.28E+06	6.14E+06	5.88E+06	4.32E+06	4.62E+06	1.99E+06	43.13	plasma	CD
1 7-Dimethylxanthine(paraxanthine)	level3f21	181.0723	3.0	7.77E+06	8.00E+06	7.57E+06	7.24E+06	1.45E+07	9.01E+06	3.06E+06	33.97	plasma	XCMS
1(2H)-Isoquinolinone	level3f22	146.0604	10.3	1.11E+07	1.48E+07	1.37E+07	1.29E+07	1.37E+07	1.32E+07	1.34E+06	10.10	plasma	LDA
1(2H)-Isoquinolinone	level3f22	146.0602	10.2	2.98E+06	3.74E+06	3.25E+06	1.48E+06	3.35E+06	2.96E+06	8.72E+05	29.43	plasma	CD
1(2H)-Isoquinolinone	level3f22	146.0629	10.2	2.27E+04	3.85E+04	3.53E+06	3.58E+04	3.03E+04	7.31E+05	1.56E+06	213.88	plasma	XCMS
3-Indoleacetonitrile	level3f23	157.0765	10.3	4.54E+07	4.18E+07	4.08E+07	3.76E+07	3.69E+07	4.05E+07	3.45E+06	8.52	plasma	LDA
3-Indoleacetonitrile	level3f23	157.0764	10.2	1.10E+07	1.07E+07	1.04E+07	1.02E+07	9.28E+06	1.03E+07	6.45E+05	6.25	plasma	CD
3-Indoleacetonitrile	level3f23	157.0763	10.3	1.14E+07	9.90E+06	1.01E+07	5.97E+04	9.07E+06	8.11E+06	4.58E+06	56.43	plasma	XCMS
4-Methylene-L-glutamine	level3f24	159.0766	22.2	1.30E+07	1.40E+07	1.45E+07	1.20E+07	1.19E+07	1.31E+07	1.17E+06	8.99	plasma	LDA
4-Methylene-L-glutamine	level3f24	159.0767	22.2	9.24E+06	1.12E+07	1.48E+07	1.28E+07	1.34E+07	1.23E+07	2.15E+06	17.45	plasma	CD
4-Methylene-L-glutamine	level3f24	159.0766	22.1	3.00E+06	3.35E+06	3.21E+06	2.76E+06	2.70E+06	3.00E+06	2.82E+05	9.38	plasma	XCMS
allylcysteine @RT:5.655	level3f25	162.0579	5.6	9.13E+08	9.47E+08	8.08E+08	8.72E+08	7.33E+08	8.55E+08	8.53E+07	9.98	plasma	LDA
allylcysteine @RT:5.655	level3f25	162.0588	5.6	2.25E+08	2.30E+08	1.90E+08	2.31E+08	1.66E+08	2.09E+08	2.91E+07	13.94	plasma	CD
allylcysteine @RT:5.655	level3f25	162.0587	5.6	1.69E+06	2.12E+08	1.94E+08	1.99E+08	1.67E+08	1.55E+08	8.71E+07	56.30	plasma	XCMS
L-Carnitinamide	level3f26	161.1287	29.4	1.92E+07	1.97E+07	2.03E+07	1.47E+07	1.26E+07	1.73E+07	3.43E+06	19.82	plasma	LDA
L-Carnitinamide	level3f26	161.1289	29.4	4.86E+06	4.68E+06	5.02E+06	3.59E+06	2.75E+06	4.18E+06	9.74E+05	23.29	plasma	CD
L-Carnitinamide	level3f26	161.1287	29.2	4.54E+06	3.74E+04	4.79E+06	3.30E+06	3.10E+06	3.15E+06	1.89E+06	60.03	plasma	XCMS
N1-Methyl-2-pyridone-5-carboxamide	level3f27	153.0661	3.7	1.30E+07	1.11E+07	1.56E+07	1.34E+07	1.44E+07	1.35E+07	1.68E+06	12.41	plasma	LDA
N1-Methyl-2-pyridone-5-carboxamide	level3f27	153.0660	3.7	3.80E+06	3.05E+06	4.26E+06	3.65E+06	4.07E+06	3.76E+06	4.67E+05	12.41	plasma	CD

N1-Methyl-2-pyridone-5-carboxamide	level3f27	153.0660	3.7	3.77E+06	2.99E+06	3.69E+06	3.47E+06	3.77E+06	3.54E+06	3.31E+05	9.35	plasma	XCMS
N-Amidino-L-aspartate	level3f28	176.0662	12.0	8.90E+06	8.50E+06	7.89E+06	7.11E+06	6.82E+06	7.84E+06	8.88E+05	11.31	plasma	LDA
N-Amidino-L-aspartate	level3f28	176.0660	11.9	2.43E+06	2.25E+06	2.09E+06	1.89E+06	1.79E+06	2.09E+06	2.60E+05	12.45	plasma	CD
N-Amidino-L-aspartate	level3f28	176.0659	12.0	2.37E+06	2.14E+06	1.96E+06	1.73E+06	1.71E+06	1.98E+06	2.79E+05	14.10	plasma	XCMS
Pregabalin	level3f29	160.1331	7.3	1.20E+08	8.31E+07	8.46E+07	8.58E+07	8.36E+07	9.14E+07	1.60E+07	17.49	plasma	LDA
Pregabalin	level3f29	160.1334	7.3	2.93E+07	2.00E+07	1.96E+07	2.15E+07	2.07E+07	2.22E+07	4.03E+06	18.13	plasma	CD
Pregabalin	level3f29	160.1335	7.2	2.17E+07	1.95E+07	2.00E+07	1.97E+07	1.95E+07	2.01E+07	9.14E+05	4.55	plasma	XCMS
tranexamic acid	level3f30	158.1179	10.2	4.85E+06	4.41E+06	4.00E+06	4.52E+06	4.27E+06	4.41E+06	3.13E+05	7.09	plasma	LDA
tranexamic acid	level3f30	158.1179	10.1	1.13E+06	1.10E+06	9.91E+05	1.12E+06	1.09E+06	1.09E+06	5.66E+04	5.20	plasma	CD
tranexamic acid	level3f30	158.1178	10.1	1.17E+06	1.09E+06	9.50E+05	1.03E+06	9.44E+05	1.04E+06	9.40E+04	9.07	plasma	XCMS
(E)-Furazolidone	level3f31	226.0453	14.7	6.46E+07	6.53E+07	5.70E+07	5.25E+07	5.00E+07	5.79E+07	6.89E+06	11.90	plasma	LDA
(E)-Furazolidone	level3f31	226.0453	14.6	1.68E+07	1.65E+07	1.42E+07	1.06E+07	9.50E+06	1.35E+07	3.33E+06	24.67	plasma	CD
[FA(18:2)]9Z 11E-octadecadienoicacid	level3f32	281.2469	1.9	4.87E+07	4.71E+07	3.06E+07	1.71E+07	1.66E+07	3.20E+07	1.55E+07	48.52	plasma	LDA
[FA(18:2)]9Z 11E-octadecadienoicacid	level3f32	281.2469	1.9	5.56E+06	9.02E+06	4.84E+06	1.53E+06	1.40E+06	4.47E+06	3.17E+06	70.84	plasma	CD
[FA(18:2)]9Z 11E-octadecadienoicacid	level3f32	281.2477	1.9	1.03E+07	9.38E+06	6.35E+06	3.38E+06	1.28E+06	6.15E+06	3.86E+06	62.69	plasma	XCMS
11-Aminoundecanoic acid	level3f33	202.1802	8.4	3.73E+07	3.41E+07	2.87E+07	2.38E+07	2.31E+07	2.94E+07	6.25E+06	21.25	plasma	LDA
11-Aminoundecanoic acid	level3f33	202.1803	8.4	8.90E+06	8.85E+06	7.23E+06	5.93E+06	5.71E+06	7.33E+06	1.53E+06	20.92	plasma	CD
11-Aminoundecanoic acid	level3f33	202.1804	8.3	9.06E+06	8.34E+06	6.92E+06	5.71E+06	5.79E+06	7.16E+06	1.50E+06	20.98	plasma	XCMS
2-[(5Z)-5-Tetradecen-1-yl]cyclobutanone	level3f34	265.2523	1.9	1.84E+07	3.05E+07	3.35E+07	3.95E+07	5.57E+07	3.55E+07	1.36E+07	38.34	plasma	LDA
2-[(5Z)-5-Tetradecen-1-yl]cyclobutanone	level3f34	265.2525	1.9	3.21E+08	4.02E+08	5.18E+08	1.24E+07	8.01E+08	4.11E+08	2.88E+08	70.00	plasma	CD
2-methylbutyrylcarnitine	level3f35	246.1701	5.8	9.81E+06	8.61E+06	8.38E+06	1.11E+07	7.55E+06	9.10E+06	1.40E+06	15.36	plasma	LDA
2-methylbutyrylcarnitine	level3f35	246.1703	5.8	2.26E+06	2.75E+06	2.18E+06	3.00E+06	2.00E+06	2.44E+06	4.19E+05	17.20	plasma	CD
2-methylbutyrylcarnitine	level3f35	246.1703	5.8	2.32E+06	2.29E+06	2.10E+06	2.86E+06	1.92E+06	2.30E+06	3.54E+05	15.39	plasma	XCMS
4-Aminoantipyrine	level3f36	204.1133	3.1	7.56E+07	6.98E+07	8.08E+07	8.60E+07	8.19E+07	7.88E+07	6.25E+06	7.93	plasma	LDA
4-Aminoantipyrine	level3f36	204.1133	3.1	1.91E+07	1.53E+07	2.14E+07	2.29E+07	2.13E+07	2.00E+07	2.98E+06	14.88	plasma	CD
4-Aminoantipyrine	level3f36	204.1135	3.1	1.84E+07	1.75E+07	1.98E+07	2.06E+07	1.95E+07	1.92E+07	1.25E+06	6.50	plasma	XCMS
alpha beta-Didehydrotryptophan	level3f37	203.0817	10.3	3.28E+07	3.31E+07	3.32E+07	3.12E+07	3.34E+07	3.28E+07	8.86E+05	2.70	plasma	LDA

alpha beta-Didehydrotryptophan	level3f37	203.0821	10.2	8.22E+06	8.52E+06	8.36E+06	8.10E+06	8.59E+06	8.36E+06	2.04E+05	2.44	plasma	CD
alpha beta-Didehydrotryptophan	level3f37	203.0820	10.3	8.42E+06	7.95E+06	8.36E+06	5.52E+04	8.26E+06	6.61E+06	3.67E+06	55.50	plasma	XCMS
Butyryl-L-carnitine	level3f38	232.1546	6.1	2.04E+07	1.80E+07	1.91E+07	1.91E+07	1.36E+07	1.80E+07	2.60E+06	14.43	plasma	LDA
Butyryl-L-carnitine	level3f38	232.1545	6.1	5.05E+06	4.61E+06	4.55E+06	4.86E+06	3.41E+06	4.49E+06	6.38E+05	14.19	plasma	CD
Butyryl-L-carnitine	level3f38	232.1545	6.1	5.00E+06	4.57E+06	4.77E+06	4.74E+06	3.49E+06	4.51E+06	5.94E+05	13.16	plasma	XCMS
CA9275000	level3f39	194.0969	2.1	9.30E+07	9.13E+07	7.67E+07	1.03E+08	9.67E+07	9.22E+07	9.77E+06	10.60	plasma	LDA
CA9275000	level3f39	194.0968	2.1	2.19E+07	2.33E+07	2.09E+07	2.58E+07	2.39E+07	2.32E+07	1.88E+06	8.11	plasma	CD
CA9275000	level3f39	194.0968	2.1	2.29E+07	2.17E+07	1.83E+07	2.51E+07	2.39E+07	2.24E+07	2.60E+06	11.61	plasma	XCMS
Dambonitol	level3f40	209.1024	2.9	2.82E+07	2.51E+07	2.16E+07	2.20E+07	2.06E+07	2.35E+07	3.11E+06	13.24	plasma	LDA
Dambonitol	level3f40	209.1023	2.9	6.43E+06	2.54E+06	4.46E+06	4.66E+06	4.75E+06	4.57E+06	1.38E+06	30.27	plasma	CD
Dambonitol	level3f40	209.1023	2.9	5.46E+06	5.18E+06	4.09E+06	4.40E+06	4.72E+06	4.77E+06	5.56E+05	11.65	plasma	XCMS
Desethylatrazine @RT:10.208	level3f41	188.0712	10.3	6.26E+07	7.42E+07	6.60E+07	6.89E+07	7.06E+07	6.85E+07	4.40E+06	6.43	plasma	LDA
Desethylatrazine @RT:10.208	level3f41	188.0709	10.2	2.73E+07	3.50E+07	3.19E+07	2.94E+07	3.21E+07	3.11E+07	2.90E+06	9.33	plasma	CD
Desethylatrazine @RT:10.208	level3f41	188.0710	10.2	1.67E+07	1.90E+07	1.80E+07	1.43E+05	5.68E+04	1.08E+07	9.78E+06	90.76	plasma	XCMS
Desethylatrazine @RT:11.996	level3f42	188.0711	12.0	1.94E+07	1.98E+07	1.90E+07	1.82E+07	1.85E+07	1.90E+07	6.37E+05	3.36	plasma	LDA
Desethylatrazine @RT:11.996	level3f42	188.0708	12.0	8.73E+06	9.02E+06	8.58E+06	8.48E+06	8.64E+06	8.69E+06	2.08E+05	2.40	plasma	CD
Desethylatrazine @RT:11.996	level3f42	188.0708	12.0	4.93E+06	4.70E+06	4.63E+06	4.32E+06	4.53E+06	4.62E+06	2.25E+05	4.87	plasma	XCMS
Desethylatrazine @RT:7.465	level3f43	188.0709	7.5	3.65E+06	8.50E+06	7.24E+06	5.70E+06	3.06E+06	5.63E+06	2.31E+06	41.06	plasma	LDA
Desethylatrazine @RT:7.465	level3f43	188.0709	7.5	1.04E+06	2.75E+06	1.78E+06	1.42E+06	7.67E+05	1.55E+06	7.71E+05	49.75	plasma	CD
Desethylatrazine @RT:7.465	level3f43	188.0710	7.5	9.29E+05	2.02E+06	1.76E+06	1.45E+06	8.25E+05	1.40E+06	5.17E+05	37.00	plasma	XCMS
Ecgonine	level3f44	186.1128	9.0	7.70E+06	7.39E+06	7.96E+06	7.79E+06	7.21E+06	7.61E+06	3.04E+05	3.99	plasma	LDA
Ecgonine	level3f44	186.1128	9.0	1.88E+06	1.80E+06	1.87E+06	1.92E+06	1.85E+06	1.87E+06	4.22E+04	2.26	plasma	CD
Ecgonine	level3f44	186.1127	8.9	1.91E+06	1.77E+06	1.91E+06	1.84E+06	1.78E+06	1.84E+06	7.00E+04	3.80	plasma	XCMS
FARNESYL ACETONE	level3f45	263.2380	1.9	7.63E+07	8.55E+07	8.64E+07	9.49E+07	6.62E+07	8.19E+07	1.09E+07	13.36	plasma	LDA
FARNESYL ACETONE	level3f45	263.2378	2.0	1.32E+08	1.70E+08	7.43E+07	1.20E+08	1.80E+08	1.35E+08	4.22E+07	31.23	plasma	CD
FARNESYL ACETONE	level3f45	263.2377	1.9	1.23E+07	1.22E+07	1.08E+07	2.20E+07	1.26E+07	1.40E+07	4.55E+06	32.55	plasma	XCMS
gamma-Glu-gln	level3f46	276.1192	23.8	1.31E+07	2.20E+07	2.21E+07	1.57E+07	1.48E+07	1.75E+07	4.22E+06	24.10	plasma	LDA
gamma-Glu-gln	level3f46	276.1193	23.8	4.20E+06	5.83E+06	6.08E+06	5.06E+06	1.48E+06	4.53E+06	1.86E+06	40.96	plasma	CD
gamma-Glu-gln	level3f46	276.1193	23.8	2.89E+06	3.84E+06	4.00E+06	2.72E+06	2.59E+06	3.21E+06	6.65E+05	20.72	plasma	XCMS
Leu-Pro(Leucyl-Proline)	level3f47	229.1548	7.8	8.37E+07	7.28E+07	6.92E+07	8.43E+07	7.06E+07	7.61E+07	7.33E+06	9.63	plasma	LDA

Leu-Pro(Leucyl-Proline)	level3f47	229.1548	7.8	1.87E+07	2.04E+07	1.21E+07	2.29E+07	8.74E+06	1.66E+07	5.92E+06	35.76	plasma	CD
Leu-Pro(Leucyl-Proline)	level3f47	229.1551	7.7	1.01E+07	7.75E+06	1.11E+07	8.71E+06	6.84E+06	8.90E+06	1.72E+06	19.36	plasma	XCMS
Linoleamide	level3f48	280.2648	1.9	4.42E+08	4.99E+08	5.54E+08	6.45E+08	7.18E+08	5.72E+08	1.11E+08	19.42	plasma	LDA
Linoleamide	level3f48	280.2647	2.0	6.47E+07	7.30E+07	1.00E+08	1.07E+08	3.03E+07	7.51E+07	3.08E+07	40.98	plasma	CD
Linoleamide	level3f48	280.2645	2.0	8.50E+07	8.14E+07	8.40E+07	1.08E+08	1.09E+08	9.34E+07	1.38E+07	14.74	plasma	XCMS
Muscone	level3f49	261.2222	1.9	3.12E+06	2.99E+06	3.37E+06	3.76E+06	2.73E+06	3.19E+06	3.94E+05	12.32	plasma	LDA
Muscone	level3f49	261.2222	2.0	7.56E+06	6.53E+06	7.35E+06	8.12E+06	8.10E+06	7.53E+06	6.51E+05	8.65	plasma	CD
Muscone	level3f49	261.2220	2.0	4.12E+05	8.17E+05	7.74E+05	7.90E+05	4.68E+05	6.52E+05	1.95E+05	29.97	plasma	XCMS
Mutagen X	level3f50	216.9233	11.7	5.76E+07	7.33E+07	8.87E+07	7.61E+07	7.53E+07	7.42E+07	1.11E+07	14.94	plasma	LDA
Mutagen X	level3f50	216.9230	11.6	2.42E+07	2.35E+07	1.83E+07	2.52E+07	1.78E+07	2.18E+07	3.47E+06	15.91	plasma	CD
Mutagen X	level3f50	216.9230	11.6	8.82E+06	1.76E+07	1.73E+07	1.13E+07	1.25E+07	1.35E+07	3.84E+06	28.47	plasma	XCMS
Noramidopyrine @RT:5.416	level3f51	218.1293	5.2	2.15E+08	1.38E+08	1.36E+08	1.45E+08	1.56E+08	1.58E+08	3.30E+07	20.89	plasma	LDA
Noramidopyrine @RT:5.416	level3f51	218.1293	5.4	3.96E+04	3.50E+07	3.67E+07	3.20E+07	4.21E+07	2.92E+07	1.67E+07	57.22	plasma	CD
Noramidopyrine @RT:5.416	level3f51	218.1292	5.1	4.76E+05	3.14E+05	3.31E+07	3.45E+07	3.73E+07	2.11E+07	1.90E+07	89.87	plasma	XCMS
octadec-9-ynoic acid	level3f52	263.2366	1.9	7.64E+07	8.55E+07	8.64E+07	9.50E+07	1.20E+08	9.26E+07	1.64E+07	17.76	plasma	LDA
octadec-9-ynoic acid	level3f52	263.2378	2.0	1.32E+08	1.70E+08	7.43E+07	1.20E+08	1.80E+08	1.35E+08	4.22E+07	31.23	plasma	CD
octadec-9-ynoic acid	level3f52	263.2377	1.9	1.23E+07	1.22E+07	1.08E+07	2.20E+07	1.26E+07	1.40E+07	4.55E+06	32.55	plasma	XCMS
PEG-4	level3f53	195.1229	2.8	1.22E+08	1.10E+08	1.10E+08	1.26E+08	1.13E+08	1.16E+08	7.37E+06	6.35	plasma	LDA
PEG-4	level3f53	195.1232	2.8	1.40E+07	2.84E+07	3.00E+07	3.21E+07	1.93E+07	2.48E+07	7.73E+06	31.22	plasma	CD
PEG-4	level3f53	195.1232	2.7	1.52E+07	1.35E+07	1.32E+07	1.41E+07	1.14E+07	1.35E+07	1.40E+06	10.36	plasma	XCMS
prilocaine	level3f54	221.1653	5.7	7.23E+07	7.76E+07	8.57E+07	1.16E+08	1.22E+08	9.47E+07	2.28E+07	24.03	plasma	LDA
prilocaine	level3f54	221.1652	5.7	1.15E+07	2.01E+07	2.23E+07	2.87E+07	1.98E+07	2.05E+07	6.18E+06	30.14	plasma	CD
prilocaine	level3f54	221.1652	5.6	8.04E+06	1.87E+07	8.36E+04	2.83E+07	2.18E+07	1.54E+07	1.13E+07	73.19	plasma	XCMS
sn-glycero-3-Phosphocholine @RT:23.413	level3f55	258.1107	23.2	2.97E+07	3.13E+07	2.87E+07	2.56E+07	2.55E+07	2.82E+07	2.55E+06	9.06	plasma	LDA
sn-glycero-3-Phosphocholine @RT:23.413	level3f55	258.1105	23.0	7.93E+06	7.57E+06	7.24E+06	3.98E+06	6.08E+06	6.56E+06	1.60E+06	24.35	plasma	CD
Symmetricdimethylarginine @RT:26.364	level3f56	203.1508	26.4	7.84E+06	6.79E+06	5.76E+06	4.41E+06	4.18E+06	5.79E+06	1.56E+06	26.88	plasma	LDA
Symmetricdimethylarginine @RT:26.364	level3f56	203.1508	26.4	2.01E+03	1.54E+06	1.43E+06	1.11E+06	1.10E+06	1.04E+06	6.10E+05	58.87	plasma	CD
Symmetricdimethylarginine	level3f56	203.1505	26.3	1.86E+06	1.62E+06	1.38E+06	1.04E+06	1.02E+06	1.39E+06	3.65E+05	26.36	plasma	XCMS

@RT:26.364													
Symmetricdimethylarginine @RT:26.646	level3f57	203.1503	26.7	9.30E+06	7.95E+06	6.91E+06	5.76E+06	5.47E+06	7.08E+06	1.59E+06	22.40	plasma	LDA
Symmetricdimethylarginine @RT:26.646	level3f57	203.1505	26.8	1.58E+06	1.82E+06	1.72E+06	1.35E+06	1.34E+06	1.56E+06	2.14E+05	13.72	plasma	CD
Symmetricdimethylarginine @RT:26.646	level3f57	203.1504	26.7	2.21E+06	1.77E+06	1.54E+06	1.26E+06	1.22E+06	1.60E+06	4.07E+05	25.44	plasma	XCMS
Tetraacetylethylenediamine	level3f58	229.1192	16.0	1.43E+07	1.49E+07	1.27E+07	1.09E+07	9.80E+06	1.25E+07	2.16E+06	17.30	plasma	LDA
Tetraacetylethylenediamine	level3f58	229.1187	15.8	9.10E+05	3.79E+06	3.26E+06	2.97E+06	2.22E+06	2.63E+06	1.12E+06	42.44	plasma	CD
Tetraacetylethylenediamine	level3f58	229.1186	15.8	2.45E+04	3.64E+06	2.76E+06	1.96E+06	2.31E+06	2.14E+06	1.34E+06	62.61	plasma	XCMS
Xylazine	level3f59	221.1104	18.1	4.76E+06	6.40E+06	3.85E+06	2.28E+06	7.94E+05	3.62E+06	2.17E+06	60.02	plasma	LDA
Xylazine	level3f59	221.1104	18.0	1.16E+06	1.54E+06	9.61E+05	5.61E+05	1.99E+05	8.84E+05	5.22E+05	59.05	plasma	CD
Xylazine	level3f59	221.1081	18.0	4.75E+03	1.51E+06	9.30E+05	4.89E+03	2.19E+03	4.89E+05	6.95E+05	142.04	plasma	XCMS
(+/-)12(13)-DiHOME	level3f60	297.2418	1.9	3.12E+07	3.01E+07	2.14E+07	1.46E+07	1.15E+07	2.18E+07	8.90E+06	40.88	plasma	LDA
(+/-)12(13)-DiHOME	level3f60	297.2427	1.9	1.13E+07	1.97E+07	1.29E+07	1.16E+07	9.96E+06	1.31E+07	3.83E+06	29.26	plasma	CD
(+/-)12(13)-DiHOME	level3f60	297.2427	1.9	1.49E+06	5.42E+06	5.09E+06	3.42E+06	1.03E+06	3.29E+06	2.01E+06	61.04	plasma	XCMS
(2R)-1-[(6Z,9Z,12Z,15Z)-6,9,12,15-Octadecatetraenoyloxy]-3-(phosphonooxy)-2-propanyl (4Z,7Z,10Z,13Z,16Z,19Z)-4,7,10,13,16,19-docosahexaenoate	level3f61	371.2285	2.6	2.61E+07	3.61E+07	3.73E+07	3.23E+07	2.71E+07	3.18E+07	5.08E+06	15.99	plasma	LDA
(2R)-1-[(6Z,9Z,12Z,15Z)-6,9,12,15-Octadecatetraenoyloxy]-3-(phosphonooxy)-2-propanyl (4Z,7Z,10Z,13Z,16Z,19Z)-4,7,10,13,16,19-docosahexaenoate	level3f61	371.2288	3.0	4.76E+07	4.73E+07	4.16E+07	4.76E+07	3.10E+07	4.30E+07	7.17E+06	16.68	plasma	CD
(2R)-1-[(6Z,9Z,12Z,15Z)-6,9,12,15-Octadecatetraenoyloxy]-3-(phosphonooxy)-2-propanyl (4Z,7Z,10Z,13Z,16Z,19Z)-4,7,10,13,16,19-docosahexaenoate	level3f61	371.2285	2.6	5.86E+06	8.87E+06	7.22E+06	7.28E+06	6.26E+06	7.10E+06	1.17E+06	16.42	plasma	XCMS
(5S)-5-hydroxy-1,7-diphenylheptan-3-one	level3f62	300.2021	2.6	1.30E+08	1.19E+08	1.10E+08	5.74E+07	1.17E+08	1.07E+08	2.85E+07	26.70	plasma	LDA
(5S)-5-hydroxy-1,7-diphenylheptan-3-one	level3f62	300.2021	2.6	1.51E+07	1.48E+07	1.17E+07	1.68E+07	1.77E+07	1.52E+07	2.31E+06	15.19	plasma	CD
(5S)-5-hydroxy-1,7-diphenylheptan-3-one	level3f62	300.2026	2.6	1.60E+07	1.56E+07	1.26E+07	1.39E+07	1.21E+07	1.40E+07	1.74E+06	12.41	plasma	XCMS

(8E)-2-Amino-8-octadecene-1,3,4-triol	level3f63	316.2848	2.2	3.58E+06	3.36E+06	2.43E+06	2.94E+06	2.92E+06	3.05E+06	4.44E+05	14.57	plasma	LDA
(8E)-2-Amino-8-octadecene-1,3,4-triol	level3f63	316.2848	2.2	2.69E+06	1.89E+07	2.09E+07	2.65E+07	2.78E+07	1.94E+07	1.00E+07	51.84	plasma	CD
(9S 10S)-9 10-Dihydroxyoctadecanoate @RT:1.903	level3f64	317.2680	1.9	3.71E+07	3.16E+07	2.55E+07	1.92E+07	1.47E+07	2.56E+07	9.05E+06	35.32	plasma	LDA
(9S 10S)-9 10-Dihydroxyoctadecanoate @RT:1.903	level3f64	317.2681	1.9	9.08E+06	7.95E+06	6.68E+06	6.17E+05	3.85E+06	5.64E+06	3.42E+06	60.62	plasma	CD
15S-hydroxyeicosatrienoic acid	level3f65	323.2565	1.9	3.96E+06	3.51E+06	4.31E+06	4.37E+06	5.45E+06	4.32E+06	7.21E+05	16.68	plasma	LDA
15S-hydroxyeicosatrienoic acid	level3f65	323.2565	1.9	6.30E+05	1.33E+06	7.36E+05	2.11E+05	4.62E+05	6.73E+05	4.16E+05	61.82	plasma	CD
15S-hydroxyeicosatrienoic acid	level3f65	323.2578	1.9	3.53E+05	6.86E+05	7.60E+05	7.24E+05	7.55E+05	6.55E+05	1.72E+05	26.21	plasma	XCMS
2-Oxooctadecanoicacid	level3f66	299.2576	1.9	3.82E+07	4.09E+07	3.59E+07	1.84E+07	1.72E+07	3.01E+07	1.14E+07	37.77	plasma	LDA
2-Oxooctadecanoicacid	level3f66	299.2577	1.9	2.50E+07	2.26E+07	1.56E+07	1.01E+07	7.83E+06	1.62E+07	7.51E+06	46.22	plasma	CD
2-Oxooctadecanoicacid	level3f66	299.2576	1.9	7.90E+06	8.23E+06	5.79E+06	4.79E+06	5.00E+06	6.34E+06	1.62E+06	25.52	plasma	XCMS
4147610	level3f67	344.2805	4.4	1.12E+07	5.80E+06	6.97E+06	7.58E+06	6.44E+06	7.59E+06	2.12E+06	27.89	plasma	LDA
4147610	level3f67	344.2803	4.4	2.74E+06	1.10E+06	1.80E+06	1.41E+06	1.58E+06	1.72E+06	6.24E+05	36.20	plasma	CD
4147610	level3f67	344.2803	4.3	2.71E+06	1.44E+06	1.47E+06	1.99E+06	1.67E+06	1.86E+06	5.25E+05	28.26	plasma	XCMS
4149853	level3f68	372.3110	4.2	3.45E+06	1.85E+06	1.30E+06	1.72E+06	7.78E+05	1.82E+06	1.00E+06	55.02	plasma	LDA
4149853	level3f68	372.3110	4.3	8.26E+05	4.02E+05	3.24E+05	3.98E+05	3.41E+05	4.58E+05	2.09E+05	45.52	plasma	CD
4149853	level3f68	372.3109	4.1	8.78E+05	4.73E+05	3.50E+05	4.91E+05	4.13E+05	5.21E+05	2.07E+05	39.69	plasma	XCMS
46644	level3f69	328.1035	9.2	4.71E+06	5.94E+06	5.92E+06	5.48E+06	3.93E+06	5.20E+06	8.66E+05	16.67	plasma	LDA
46644	level3f69	328.1035	9.1	4.11E+06	5.37E+06	5.12E+06	3.90E+06	4.01E+06	4.50E+06	6.86E+05	15.25	plasma	CD
46644	level3f69	328.1032	9.1	1.16E+06	1.54E+06	1.51E+06	1.42E+06	1.03E+06	1.33E+06	2.26E+05	16.98	plasma	XCMS
4-methoxy-6-(2-{4-[(3-methylbut-2-en-1-yl)oxy]phenyl}ethyl)-2H-1,3-benzodioxole @RT:2.635	level3f70	358.2075	2.5	3.50E+07	4.85E+07	3.50E+07	3.35E+07	3.22E+07	3.68E+07	6.64E+06	18.02	plasma	LDA
4-methoxy-6-(2-{4-[(3-methylbut-2-en-1-yl)oxy]phenyl}ethyl)-2H-1,3-benzodioxole @RT:2.635	level3f70	358.2075	2.6	2.26E+06	1.53E+07	7.11E+06	1.16E+07	9.80E+06	9.22E+06	4.90E+06	53.13	plasma	CD
4-methoxy-6-(2-{4-[(3-methylbut-2-en-1-yl)oxy]phenyl}ethyl)-2H-1,3-benzodioxole @RT:2.635	level3f70	358.2082	3.0	9.60E+06	1.02E+07	8.53E+06	9.12E+06	8.31E+06	9.15E+06	7.70E+05	8.42	plasma	XCMS
8 11 14-Eicosatrienoicacid	level3f71	307.2623	1.9	1.38E+07	1.47E+07	1.45E+07	1.52E+07	1.77E+07	1.52E+07	1.46E+06	9.62	plasma	LDA

8 11 14-Eicosatrienoicacid	level3f71	307.2623	2.0	3.38E+06	3.85E+06	3.14E+04	1.04E+06	2.41E+06	2.14E+06	1.60E+06	74.46	plasma	CD
ADBICA	level3f72	344.2291	2.6	3.79E+08	2.67E+08	3.31E+08	1.74E+08	1.82E+08	2.66E+08	9.02E+07	33.85	plasma	LDA
ADBICA	level3f72	344.2291	3.0	3.29E+07	3.12E+07	3.07E+07	5.76E+07	3.44E+07	3.74E+07	1.14E+07	30.55	plasma	CD
ADBICA	level3f72	344.2292	2.6	4.55E+07	4.11E+07	3.80E+07	4.13E+07	3.67E+07	4.05E+07	3.44E+06	8.49	plasma	XCMS
Icosadienoicacid	level3f73	309.2789	1.8	1.32E+08	1.18E+08	1.44E+08	1.97E+08	2.15E+08	1.61E+08	4.23E+07	26.23	plasma	LDA
Icosadienoicacid	level3f73	309.2789	1.9	3.35E+07	2.78E+07	3.77E+07	4.21E+07	5.11E+07	3.85E+07	8.82E+06	22.94	plasma	CD
Icosadienoicacid	level3f73	309.2788	1.8	3.38E+07	3.02E+07	3.37E+07	4.48E+07	5.47E+07	3.95E+07	1.01E+07	25.72	plasma	XCMS
PALGLY	level3f74	314.2692	2.1	4.20E+06	2.72E+06	2.80E+06	3.62E+06	3.04E+06	3.28E+06	6.24E+05	19.05	plasma	LDA
PALGLY	level3f74	314.2694	2.1	9.45E+05	6.81E+05	5.70E+05	8.04E+05	7.24E+05	7.45E+05	1.40E+05	18.83	plasma	CD
PALGLY	level3f74	314.2695	2.1	9.12E+05	7.08E+05	6.48E+05	8.90E+05	6.23E+05	7.56E+05	1.36E+05	18.01	plasma	XCMS
trans-2-Dodecenoylcarnitine	level3f75	342.2650	4.4	1.71E+07	9.42E+06	1.63E+06	1.66E+07	1.64E+07	1.22E+07	6.71E+06	54.93	plasma	LDA
trans-2-Dodecenoylcarnitine	level3f75	342.2646	4.4	5.12E+06	2.53E+06	3.22E+06	4.32E+06	4.37E+06	3.91E+06	1.03E+06	26.27	plasma	CD
trans-2-Dodecenoylcarnitine	level3f75	342.2646	4.4	4.33E+06	2.93E+06	3.05E+06	3.92E+06	4.12E+06	3.67E+06	6.40E+05	17.44	plasma	XCMS
UNII:OUT5YHB7BO	level3f76	298.2745	1.9	7.42E+07	7.74E+07	8.71E+07	9.54E+07	1.15E+08	8.98E+07	1.63E+07	18.13	plasma	LDA
UNII:OUT5YHB7BO	level3f76	298.2745	1.9	1.76E+06	2.07E+07	2.27E+07	2.60E+07	3.07E+07	2.04E+07	1.11E+07	54.34	plasma	CD
(19R,25S)-22,25,28,28-Tetrahydroxy-22,28-dioxido-16-oxo-17,21,23,27-tetraoxa-22lambda~5~,28lambda~5~-diphosphaoctacosan-19-yl (11Z)-11-octadecenoate	level3f77	415.2545	2.6	8.69E+06	1.14E+07	1.22E+07	9.91E+06	1.07E+07	1.06E+07	1.34E+06	12.70	plasma	LDA
(19R,25S)-22,25,28,28-Tetrahydroxy-22,28-dioxido-16-oxo-17,21,23,27-tetraoxa-22lambda~5~,28lambda~5~-diphosphaoctacosan-19-yl (11Z)-11-octadecenoate	level3f77	415.2639	2.6	5.35E+05	4.46E+05	6.26E+05	4.49E+05	4.84E+05	5.08E+05	7.51E+04	14.78	plasma	CD
(19R,25S)-22,25,28,28-Tetrahydroxy-22,28-dioxido-16-oxo-17,21,23,27-tetraoxa-22lambda~5~,28lambda~5~-diphosphaoctacosan-19-yl (11Z)-11-octadecenoate	level3f77	415.2543	2.6	1.38E+06	2.54E+06	2.29E+06	3.58E+05	2.13E+06	1.74E+06	8.85E+05	50.93	plasma	XCMS
(3Z,6Z,9Z,12Z,21R)-27-Amino-24-hydroxy-24-oxido-18-oxo-19,23,25-	level3f78	392.7488	2.6	1.08E+07	8.24E+06	1.63E+07	1.68E+07	1.59E+07	1.36E+07	3.86E+06	28.33	plasma	LDA

trioxa-24lambda~5~-phosphaheptacosa-3,6,9,12-tetraen-21-yl (4Z,7Z,10Z,13Z,16Z,19Z)-4,7,10,13,16,19-docosahexaenoate													
(3Z,6Z,9Z,12Z,21R)-27-Amino-24-hydroxy-24-oxido-18-oxo-19,23,25-trioxa-24lambda~5~-phosphaheptacosa-3,6,9,12-tetraen-21-yl (4Z,7Z,10Z,13Z,16Z,19Z)-4,7,10,13,16,19-docosahexaenoate	level3f78	392.7494	2.6	1.54E+06	1.88E+06	1.44E+06	1.34E+06	2.32E+06	1.71E+06	4.00E+05	23.44	plasma	CD
(3Z,6Z,9Z,12Z,21R)-27-Amino-24-hydroxy-24-oxido-18-oxo-19,23,25-trioxa-24lambda~5~-phosphaheptacosa-3,6,9,12-tetraen-21-yl (4Z,7Z,10Z,13Z,16Z,19Z)-4,7,10,13,16,19-docosahexaenoate	level3f78	392.7492	2.8	2.88E+06	2.92E+06	2.60E+06	2.47E+06	2.61E+06	2.70E+06	1.93E+05	7.16	plasma	XCMS
2-{5-[2-({2-[5-(2-hydroxybutyl)oxolan-2-yl]propanoyl}oxy)butyl]oxolan-2-yl}propanoic acid @RT:2.644	level3f80	432.2816	2.6	2.03E+08	2.23E+08	1.97E+08	2.24E+08	2.04E+08	2.10E+08	1.25E+07	5.95	plasma	LDA
2-{5-[2-({2-[5-(2-hydroxybutyl)oxolan-2-yl]propanoyl}oxy)butyl]oxolan-2-yl}propanoic acid @RT:2.644	level3f80	432.2819	2.6	8.16E+07	8.34E+07	7.10E+07	7.72E+07	7.43E+07	7.75E+07	5.10E+06	6.58	plasma	CD
2-{5-[2-({2-[5-(2-hydroxybutyl)oxolan-2-yl]propanoyl}oxy)butyl]oxolan-2-yl}propanoic acid @RT:2.644	level3f80	432.2820	2.6	5.64E+07	5.44E+07	4.57E+07	4.88E+07	5.49E+07	5.20E+07	4.55E+06	8.74	plasma	XCMS
2-{5-[2-({2-[5-(2-hydroxybutyl)oxolan-2-yl]propanoyl}oxy)butyl]oxolan-2-yl}propanoic acid @RT:3.041	level3f81	432.2816	3.0	1.66E+08	1.74E+08	1.35E+08	1.52E+08	1.51E+08	1.56E+08	1.50E+07	9.62	plasma	LDA
2-{5-[2-({2-[5-(2-hydroxybutyl)oxolan-2-yl]propanoyl}oxy)butyl]oxolan-2-yl}propanoic acid @RT:3.041	level3f81	432.2821	3.0	3.62E+07	3.82E+07	3.48E+07	3.58E+07	3.28E+07	3.56E+07	1.96E+06	5.52	plasma	CD
2-{5-[2-({2-[5-(2-hydroxybutyl)oxolan-2-	level3f81	432.2814	3.1	3.79E+07	3.96E+07	3.28E+07	3.44E+07	3.39E+07	3.57E+07	2.90E+06	8.13	plasma	XCMS

yl]propanoyl}oxy)butyl]oxolan-2-yl}propanoic acid @RT:3.041													
MFCD22416941	level3f82	426.3588	2.7	2.25E+06	1.65E+06	6.31E+05	1.24E+06	3.78E+06	1.91E+06	1.20E+06	62.85	plasma	LDA
MFCD22416941	level3f82	426.3588	2.6	1.45E+05	1.69E+05	1.10E+05	6.44E+05	9.69E+05	4.07E+05	3.82E+05	93.88	plasma	CD
Militarinone A	level3f83	460.2715	2.0	3.19E+06	9.22E+06	1.80E+07	1.78E+07	5.19E+06	1.07E+07	6.94E+06	64.96	plasma	LDA
Militarinone A	level3f83	460.2709	2.0	8.36E+05	2.14E+06	5.84E+06	4.78E+06	1.58E+06	3.04E+06	2.16E+06	71.17	plasma	CD
1-[(9Z)-hexadecenoyl]-sn-glycero-3-phosphocholine	level3f84	494.3267	7.8	5.73E+07	6.75E+07	5.66E+07	6.29E+07	6.45E+07	6.18E+07	4.71E+06	7.62	plasma	LDA
1-[(9Z)-hexadecenoyl]-sn-glycero-3-phosphocholine	level3f84	494.3266	7.8	1.87E+07	1.96E+07	1.85E+07	1.97E+07	1.88E+07	1.91E+07	5.55E+05	2.91	plasma	CD
1-[(9Z)-hexadecenoyl]-sn-glycero-3-phosphocholine	level3f84	494.3262	7.7	1.43E+07	1.20E+07	1.41E+07	1.23E+07	1.38E+07	1.33E+07	1.05E+06	7.93	plasma	XCMS
1-heptadecanoyl-sn-glycero-3-phosphocholine	level3f85	510.3562	8.1	1.51E+07	1.39E+07	1.31E+07	1.46E+07	1.84E+07	1.50E+07	2.05E+06	13.65	plasma	LDA
1-heptadecanoyl-sn-glycero-3-phosphocholine	level3f85	510.3565	8.1	4.31E+06	3.73E+06	3.65E+06	5.47E+06	4.69E+06	4.37E+06	7.49E+05	17.13	plasma	CD
1-heptadecanoyl-sn-glycero-3-phosphocholine	level3f85	510.3566	8.1	4.12E+06	3.46E+06	3.23E+06	3.85E+06	5.78E+06	4.09E+06	1.01E+06	24.60	plasma	XCMS
1-Linoleoylglycerophosphocholine	level3f86	520.3409	7.9	7.69E+08	1.55E+09	1.19E+09	1.00E+09	8.86E+08	1.08E+09	3.03E+08	28.17	plasma	LDA
1-Linoleoylglycerophosphocholine	level3f86	520.3414	7.5	2.19E+08	2.94E+08	2.06E+08	2.72E+08	2.59E+08	2.50E+08	3.67E+07	14.66	plasma	CD
1-Linoleoylglycerophosphocholine	level3f86	520.3415	7.5	1.23E+08	2.17E+08	1.46E+08	2.26E+08	2.32E+08	1.89E+08	5.05E+07	26.72	plasma	XCMS
1-O-Hexadecyl-lyso-sn-glycero-3-phosphocholine	level3f87	482.3611	8.4	4.30E+06	8.11E+06	5.65E+06	7.15E+06	3.67E+06	5.78E+06	1.87E+06	32.35	plasma	LDA
1-O-Hexadecyl-lyso-sn-glycero-3-phosphocholine	level3f87	482.3611	8.5	1.87E+06	1.77E+06	9.03E+05	3.90E+05	3.38E+05	1.06E+06	7.36E+05	69.73	plasma	CD
1-O-Hexadecyl-lyso-sn-glycero-3-phosphocholine	level3f87	482.3612	8.5	1.73E+06	1.36E+06	9.72E+05	7.71E+05	4.70E+05	1.06E+06	4.96E+05	46.75	plasma	XCMS
1-Oleoylglycerophosphocholine @RT:7.403	level3f88	522.3574	7.3	5.20E+08	5.52E+08	5.18E+08	5.20E+08	7.09E+08	5.64E+08	8.21E+07	14.56	plasma	LDA
1-Oleoylglycerophosphocholine @RT:7.403	level3f88	522.3568	7.4	1.42E+08	1.19E+08	1.62E+08	9.25E+07	1.84E+08	1.40E+08	3.59E+07	25.61	plasma	CD
1-Oleoylglycerophosphocholine @RT:7.403	level3f88	522.3568	7.4	5.65E+07	1.10E+08	1.18E+08	1.57E+08	1.21E+08	1.13E+08	3.62E+07	32.11	plasma	XCMS
1-Oleoylglycerophosphocholine @RT:8.184	level3f89	522.3566	8.1	4.41E+08	5.39E+08	5.25E+08	5.20E+08	5.75E+08	5.20E+08	4.92E+07	9.47	plasma	LDA
1-Oleoylglycerophosphocholine @RT:8.184	level3f89	522.3568	8.1	1.62E+08	8.29E+07	1.44E+08	1.33E+08	1.87E+08	1.42E+08	3.88E+07	27.29	plasma	CD
1-Oleoylglycerophosphocholine	level3f89	522.3566	8.0	1.08E+08	9.11E+07	9.38E+07	1.30E+08	1.15E+08	1.08E+08	1.60E+07	14.89	plasma	XCMS

@RT:8.184														
1-Palmitoylglycerophosphocholine	level3f90	496.3419	8.0	2.24E+09	3.99E+09	8.80E+08	2.32E+09	3.41E+09	2.57E+09	1.20E+09	46.64	plasma	LDA
1-Palmitoylglycerophosphocholine	level3f90	496.3414	7.7	6.84E+08	5.72E+08	5.90E+08	6.52E+08	6.91E+08	6.38E+08	5.41E+07	8.49	plasma	CD
1-Palmitoylglycerophosphocholine	level3f90	496.3417	7.6	4.72E+08	4.17E+08	5.39E+08	4.58E+08	3.78E+08	4.53E+08	6.05E+07	13.37	plasma	XCMS
1-stearoyl-sn-glycero-3-phosphoethanolamine	level3f91	482.3252	8.0	2.38E+07	9.76E+06	3.44E+07	2.11E+07	2.09E+07	2.20E+07	8.78E+06	39.97	plasma	LDA
1-stearoyl-sn-glycero-3-phosphoethanolamine	level3f91	482.3256	8.1	5.38E+06	4.52E+06	3.90E+06	6.39E+06	6.33E+06	5.30E+06	1.10E+06	20.76	plasma	CD
1-stearoyl-sn-glycero-3-phosphoethanolamine	level3f91	482.3255	8.4	4.74E+06	4.97E+06	3.93E+06	3.75E+06	3.66E+06	4.21E+06	5.99E+05	14.22	plasma	XCMS
2-linoleoyl-sn-glycero-3-phosphoethanolamine	level3f92	478.2940	7.7	3.04E+07	1.73E+07	1.56E+07	3.15E+07	1.30E+07	2.16E+07	8.71E+06	40.40	plasma	LDA
2-linoleoyl-sn-glycero-3-phosphoethanolamine	level3f92	478.2942	7.7	3.89E+06	4.76E+06	4.39E+06	2.99E+06	4.54E+06	4.11E+06	7.07E+05	17.20	plasma	CD
2-linoleoyl-sn-glycero-3-phosphoethanolamine	level3f92	478.2940	7.7	3.83E+06	4.26E+06	3.29E+06	3.84E+06	3.48E+06	3.74E+06	3.73E+05	9.96	plasma	XCMS
L-alpha-lysophosphatidylcholine	level3f93	468.3094	8.2	3.55E+07	6.31E+07	3.27E+07	3.25E+07	2.80E+07	3.84E+07	1.41E+07	36.69	plasma	LDA
L-alpha-lysophosphatidylcholine	level3f93	468.3096	8.5	1.21E+07	1.22E+07	9.41E+06	7.85E+06	7.28E+06	9.76E+06	2.30E+06	23.57	plasma	CD
L-alpha-lysophosphatidylcholine	level3f93	468.3096	8.5	9.34E+06	8.75E+06	7.28E+06	6.09E+06	4.98E+06	7.29E+06	1.81E+06	24.82	plasma	XCMS
LysoPC(18:3(9Z,12Z,15Z)) @RT:7.699	level3f94	518.3232	7.7	2.78E+07	1.31E+07	9.90E+06	1.25E+07	1.25E+07	1.51E+07	7.20E+06	47.53	plasma	LDA
LysoPC(18:3(9Z,12Z,15Z)) @RT:7.699	level3f94	518.3234	7.7	8.00E+06	3.25E+06	1.96E+06	3.03E+06	2.70E+06	3.79E+06	2.40E+06	63.47	plasma	CD
LysoPC(18:3(9Z,12Z,15Z)) @RT:8.360	level3f95	518.3228	8.3	4.80E+07	2.61E+07	4.05E+07	5.07E+07	5.50E+07	4.40E+07	1.13E+07	25.71	plasma	LDA
LysoPC(18:3(9Z,12Z,15Z)) @RT:8.360	level3f95	518.3233	8.3	1.32E+07	6.72E+06	1.19E+07	1.33E+07	1.16E+07	1.13E+07	2.69E+06	23.75	plasma	CD
LysoPC(18:3(9Z,12Z,15Z)) @RT:8.360	level3f95	518.3232	8.2	1.14E+07	5.57E+06	1.02E+07	1.31E+07	1.26E+07	1.06E+07	3.01E+06	28.47	plasma	XCMS
Netilmicin @RT:2.622	level3f96	476.3078	2.6	2.24E+08	1.88E+08	1.72E+08	1.79E+08	1.68E+08	1.86E+08	2.26E+07	12.11	plasma	LDA
Netilmicin @RT:2.622	level3f96	476.3083	2.6	4.49E+07	3.21E+07	5.70E+07	6.17E+07	5.88E+07	5.09E+07	1.23E+07	24.19	plasma	CD
Netilmicin @RT:2.622	level3f96	476.3084	2.6	4.92E+07	4.57E+07	4.11E+07	4.26E+07	3.91E+07	4.35E+07	3.97E+06	9.12	plasma	XCMS
Netilmicin @RT:3.065	level3f97	476.3074	3.1	1.43E+08	1.23E+08	1.09E+08	1.25E+08	1.06E+08	1.21E+08	1.47E+07	12.14	plasma	LDA
Netilmicin @RT:3.065	level3f97	476.3081	3.1	2.94E+07	1.83E+07	2.77E+07	2.85E+07	2.74E+07	2.63E+07	4.51E+06	17.17	plasma	CD
Netilmicin @RT:3.065	level3f97	476.3077	3.1	3.17E+07	3.06E+07	2.66E+07	2.75E+07	2.62E+07	2.85E+07	2.45E+06	8.60	plasma	XCMS
[FA(18:1)]9Z-Octadecen-12-	level3f98	557.4543	1.9	3.77E+06	3.38E+06	3.77E+06	3.02E+06	1.94E+06	3.18E+06	7.59E+05	23.90	plasma	LDA

ynoicacid													
[FA(18:1)]9Z-Octadecen-12-ynoicacid	level3f98	557.4570	1.9	1.25E+06	9.62E+05	7.39E+05	8.21E+05	6.36E+05	8.81E+05	2.37E+05	26.85	plasma	CD
1-[(8Z,11Z,14Z)-icosatrienoyl]-sn-glycero-3-phosphocholine	level3f99	546.3564	7.4	3.06E+07	3.39E+07	3.22E+07	3.85E+07	4.19E+07	3.54E+07	4.65E+06	13.12	plasma	LDA
1-[(8Z,11Z,14Z)-icosatrienoyl]-sn-glycero-3-phosphocholine	level3f99	546.3562	7.3	7.39E+06	6.94E+06	9.67E+06	1.01E+07	5.79E+06	7.97E+06	1.83E+06	22.99	plasma	CD
1-[(8Z,11Z,14Z)-icosatrienoyl]-sn-glycero-3-phosphocholine	level3f99	546.3561	7.3	6.58E+06	4.95E+06	7.74E+06	9.46E+06	9.85E+06	7.72E+06	2.03E+06	26.31	plasma	XCMS
NP-012766 @RT:2.667	level3f100	604.3556	2.7	3.85E+07	3.46E+07	2.77E+07	4.16E+07	2.72E+07	3.39E+07	6.39E+06	18.83	plasma	LDA
NP-012766 @RT:2.667	level3f100	604.3554	2.7	7.36E+06	6.25E+06	6.00E+06	6.34E+06	5.27E+06	6.24E+06	7.53E+05	12.06	plasma	CD
NP-012766 @RT:2.667	level3f100	604.3555	2.9	1.51E+07	1.41E+07	1.34E+07	1.45E+07	1.23E+07	1.39E+07	1.08E+06	7.75	plasma	XCMS
NP-012766 @RT:2.901	level3f101	604.3551	2.9	6.47E+07	6.18E+07	5.59E+07	6.06E+07	4.95E+07	5.85E+07	5.97E+06	10.21	plasma	LDA
NP-012766 @RT:2.901	level3f101	604.3551	2.9	1.77E+07	1.73E+07	1.59E+07	1.89E+07	1.57E+07	1.71E+07	1.31E+06	7.66	plasma	CD
NP-012766 @RT:2.901	level3f101	604.3555	2.9	1.51E+07	1.41E+07	1.34E+07	1.45E+07	1.23E+07	1.39E+07	1.08E+06	7.75	plasma	XCMS
NP-012766 @RT:3.301	level3f102	604.3556	3.3	9.50E+07	8.03E+07	7.90E+07	8.56E+07	7.64E+07	8.32E+07	7.37E+06	8.86	plasma	LDA
NP-012766 @RT:3.301	level3f102	604.3553	3.3	2.77E+07	2.39E+07	2.55E+07	1.99E+07	2.15E+07	2.37E+07	3.09E+06	13.06	plasma	CD
NP-012766 @RT:3.301	level3f102	604.3553	3.3	2.31E+07	2.04E+07	1.86E+07	1.90E+07	1.76E+07	1.97E+07	2.12E+06	10.76	plasma	XCMS
NP-012767	level3f103	560.3293	2.9	7.76E+07	8.56E+07	6.62E+07	7.31E+07	6.19E+07	7.29E+07	9.33E+06	12.80	plasma	LDA
NP-012767	level3f103	560.3289	2.9	2.20E+07	1.80E+07	4.96E+06	1.54E+07	1.45E+07	1.50E+07	6.30E+06	42.14	plasma	CD
NP-012767	level3f103	560.3290	2.9	1.24E+07	1.14E+07	1.13E+07	1.19E+07	1.01E+07	1.14E+07	8.60E+05	7.53	plasma	XCMS
NP-019848	level3f104	584.3583	5.4	1.98E+08	1.67E+08	5.38E+07	1.69E+08	1.46E+08	1.47E+08	5.52E+07	37.58	plasma	LDA
NP-019848	level3f104	584.3573	5.4	2.05E+07	1.79E+07	2.07E+07	1.65E+07	1.48E+07	1.81E+07	2.56E+06	14.15	plasma	XCMS
NP-019848	level3f104	584.3583	5.4	5.29E+07	3.53E+07	3.50E+07	4.06E+07	3.37E+07	3.95E+07	7.97E+06	20.19	plasma	CD
Unkown	level4f1	503.2945	4.7	3.37E+07	3.38E+07	3.30E+07	1.66E+07	2.11E+07	2.76E+07	8.17E+06	29.55	plasma	LDA
Unkown	level4f1	503.2941	4.7	4.81E+06	5.54E+06	4.81E+06	2.90E+06	3.74E+06	4.36E+06	1.04E+06	23.82	plasma	XCMS
Unkown	level4f1	503.2945	4.7	9.69E+06	4.67E+06	7.27E+06	5.26E+06	4.19E+06	6.22E+06	2.27E+06	36.49	plasma	CD
Unkown	level4f2	509.3128	3.1	3.68E+07	2.95E+07	3.04E+07	3.25E+07	2.34E+07	3.05E+07	4.88E+06	15.99	plasma	LDA
Unkown	level4f2	509.3125	3.1	9.72E+06	7.57E+06	7.94E+06	8.44E+06	6.40E+06	8.01E+06	1.22E+06	15.17	plasma	XCMS
Unkown	level4f2	509.3128	3.1	1.03E+07	1.01E+07	1.02E+07	1.15E+07	8.25E+06	1.01E+07	1.17E+06	11.57	plasma	CD
Unkown	level4f3	509.3125	3.6	6.23E+07	5.96E+07	5.49E+07	5.37E+07	4.54E+07	5.52E+07	6.49E+06	11.76	plasma	LDA
Unkown	level4f3	509.3126	3.6	1.30E+07	1.53E+07	1.61E+07	2.83E+07	1.16E+07	1.68E+07	6.64E+06	39.44	plasma	XCMS

Unkown	level4f3	509.3125	3.6	1.96E+07	2.16E+07	2.23E+07	2.21E+07	1.70E+07	2.05E+07	2.23E+06	10.88	plasma	CD
Unkown	level4f4	509.3133	2.8	7.99E+06	6.39E+06	5.18E+06	5.70E+06	5.65E+06	6.18E+06	1.10E+06	17.77	plasma	LDA
Unkown	level4f4	509.3125	3.1	9.72E+06	7.57E+06	7.94E+06	8.44E+06	6.40E+06	8.01E+06	1.22E+06	15.17	plasma	XCMS
Unkown	level4f4	509.3133	2.8	3.34E+06	1.62E+06	2.27E+06	1.15E+07	8.25E+06	5.40E+06	4.30E+06	79.56	plasma	CD
Unkown	level4f5	120.0811	10.2	1.07E+08	8.70E+07	8.93E+07	9.37E+07	1.02E+08	9.59E+07	8.63E+06	9.00	plasma	LDA
Unkown	level4f5	120.0810	10.1	1.69E+07	2.00E+07	1.29E+07	2.05E+07	2.24E+05	1.41E+07	8.33E+06	59.05	plasma	XCMS
Unkown	level4f5	120.0811	10.1	4.22E+05	3.46E+05	3.43E+05	3.56E+05	4.18E+05	3.77E+05	3.96E+04	10.50	plasma	CD
Unkown	level4f6	511.3275	3.5	3.18E+06	2.94E+06	2.35E+06	2.89E+06	2.54E+06	2.78E+06	3.30E+05	11.85	plasma	LDA
Unkown	level4f6	511.3272	3.5	7.56E+05	6.20E+05	5.80E+05	7.09E+05	6.00E+05	6.53E+05	7.58E+04	11.60	plasma	XCMS
Unkown	level4f6	511.3275	3.5	6.06E+05	6.64E+05	4.53E+05	6.52E+05	6.80E+05	6.11E+05	9.24E+04	15.13	plasma	CD
Unkown	level4f7	511.3727	2.0	4.80E+06	4.03E+06	3.84E+06	6.27E+06	6.36E+06	5.06E+06	1.20E+06	23.76	plasma	LDA
Unkown	level4f7	511.3727	2.0	1.13E+06	1.13E+06	9.03E+05	2.27E+06	2.14E+06	1.51E+06	6.39E+05	42.26	plasma	CD
Unkown	level4f8	518.3192	5.3	1.32E+08	1.29E+08	1.12E+08	1.17E+08	4.41E+07	1.07E+08	3.61E+07	33.79	plasma	LDA
Unkown	level4f8	518.3177	5.3	1.90E+07	1.59E+07	1.31E+07	1.65E+07	8.77E+06	1.46E+07	3.90E+06	26.65	plasma	XCMS
Unkown	level4f8	518.3192	5.3	9.75E+06	2.69E+07	1.55E+07	2.57E+07	9.78E+06	1.75E+07	8.36E+06	47.70	plasma	CD
Unkown	level4f9	525.3063	5.1	1.20E+07	9.68E+06	1.44E+07	1.03E+07	6.03E+06	1.05E+07	3.09E+06	29.45	plasma	LDA
Unkown	level4f9	525.3071	4.7	3.35E+06	3.71E+06	4.16E+06	2.72E+06	2.91E+06	3.37E+06	5.88E+05	17.46	plasma	XCMS
Unkown	level4f9	525.3063	5.1	4.28E+06	2.65E+06	5.21E+06	5.63E+06	2.97E+06	4.15E+06	1.32E+06	31.89	plasma	CD
Unkown	level4f10	525.3072	4.8	1.26E+07	9.91E+06	1.63E+07	1.90E+07	4.75E+06	1.25E+07	5.57E+06	44.51	plasma	LDA
Unkown	level4f10	525.3071	4.7	3.35E+06	3.71E+06	4.16E+06	2.72E+06	2.91E+06	3.37E+06	5.88E+05	17.46	plasma	XCMS
Unkown	level4f10	525.3072	4.8	5.96E+06	5.35E+06	6.53E+06	5.63E+06	3.64E+06	5.42E+06	1.09E+06	20.11	plasma	CD
Unkown	level4f11	531.3272	2.6	2.86E+06	1.96E+06	2.38E+06	2.67E+06	1.86E+06	2.34E+06	4.37E+05	18.64	plasma	LDA
Unkown	level4f11	531.3272	2.6	1.19E+06	1.30E+06	9.26E+05	7.89E+05	1.15E+06	1.07E+06	2.06E+05	19.31	plasma	CD
Unkown	level4f12	531.3272	3.7	3.50E+07	3.24E+07	2.91E+07	2.58E+07	2.40E+07	2.93E+07	4.55E+06	15.56	plasma	LDA
Unkown	level4f12	531.3272	3.7	1.03E+07	1.22E+07	1.21E+07	9.98E+06	8.13E+06	1.05E+07	1.69E+06	16.04	plasma	CD
Unkown	level4f12	531.3257	3.7	7.90E+06	8.12E+06	7.48E+06	6.89E+06	6.52E+06	7.38E+06	6.71E+05	9.08	plasma	XCMS
Unkown	level4f13	531.3272	3.2	1.75E+07	1.42E+07	1.20E+07	1.14E+07	1.01E+07	1.30E+07	2.91E+06	22.31	plasma	LDA
Unkown	level4f13	531.3256	3.2	6.15E+06	3.62E+06	3.42E+06	3.30E+06	2.65E+06	3.83E+06	1.35E+06	35.17	plasma	XCMS
Unkown	level4f13	531.3272	3.2	6.42E+06	5.34E+06	3.37E+06	3.51E+06	1.77E+06	4.08E+06	1.82E+06	44.60	plasma	CD
Unkown	level4f14	531.3700	2.0	5.36E+06	3.35E+06	3.69E+06	6.77E+06	6.80E+06	5.19E+06	1.64E+06	31.62	plasma	LDA

Unkown	level4f14	531.3700	2.0	2.05E+06	1.23E+06	2.08E+06	2.22E+06	2.79E+06	2.07E+06	5.60E+05	27.01	plasma	CD
Unkown	level4f15	531.8267	3.3	4.32E+06	3.44E+06	3.06E+06	1.25E+07	3.80E+06	5.43E+06	4.01E+06	73.74	plasma	LDA
Unkown	level4f15	531.8268	3.2	1.29E+06	1.34E+06	1.06E+06	1.15E+06	1.11E+06	1.19E+06	1.20E+05	10.12	plasma	XCMS
Unkown	level4f15	531.8267	3.3	7.69E+05	2.30E+06	4.12E+05	2.01E+06	2.35E+05	1.14E+06	9.46E+05	82.68	plasma	CD
Unkown	level4f16	533.3855	2.0	4.73E+06	3.98E+06	4.33E+06	6.65E+06	1.02E+07	5.98E+06	2.58E+06	43.15	plasma	LDA
Unkown	level4f16	533.3855	2.0	1.52E+06	1.40E+06	1.53E+06	2.57E+06	3.27E+06	2.06E+06	8.24E+05	40.07	plasma	CD
Unkown	level4f17	540.3307	5.3	1.17E+08	1.31E+08	1.23E+08	1.24E+08	4.89E+07	1.09E+08	3.38E+07	31.07	plasma	LDA
Unkown	level4f17	540.3311	5.3	1.18E+07	3.38E+07	1.34E+07	1.93E+07	1.50E+07	1.87E+07	8.92E+06	47.78	plasma	XCMS
Unkown	level4f17	540.3307	5.3	5.17E+07	2.74E+07	2.09E+07	4.64E+07	8.30E+06	3.09E+07	1.80E+07	58.14	plasma	CD
Unkown	level4f18	553.3389	3.7	1.48E+07	1.43E+07	1.34E+07	1.23E+07	1.09E+07	1.31E+07	1.55E+06	11.83	plasma	LDA
Unkown	level4f18	553.3385	3.7	2.57E+06	3.77E+06	3.57E+06	3.35E+06	2.91E+06	3.24E+06	4.90E+05	15.13	plasma	XCMS
Unkown	level4f18	553.3389	3.7	4.14E+06	5.34E+06	4.84E+06	5.60E+06	3.07E+06	4.60E+06	1.02E+06	22.21	plasma	CD
Unkown	level4f19	553.3387	3.2	6.26E+06	5.76E+06	6.18E+06	5.79E+06	4.32E+06	5.66E+06	7.83E+05	13.84	plasma	LDA
Unkown	level4f19	553.3382	3.2	2.25E+06	1.28E+06	2.10E+06	1.61E+06	1.18E+06	1.69E+06	4.81E+05	28.51	plasma	XCMS
Unkown	level4f19	553.3387	3.2	2.77E+06	1.64E+06	1.56E+06	1.96E+06	1.11E+06	1.81E+06	6.17E+05	34.11	plasma	CD
Unkown	level4f20	553.3828	2.0	7.35E+06	5.38E+06	4.40E+06	8.10E+06	1.03E+07	7.11E+06	2.34E+06	32.83	plasma	LDA
Unkown	level4f20	553.3829	2.0	1.78E+06	1.31E+06	9.50E+05	2.00E+06	2.33E+06	1.67E+06	5.48E+05	32.75	plasma	XCMS
Unkown	level4f20	553.3828	2.0	3.17E+06	1.91E+06	1.93E+06	2.84E+06	5.16E+06	3.00E+06	1.33E+06	44.13	plasma	CD
Unkown	level4f21	555.3969	2.0	3.79E+06	3.67E+06	2.76E+06	4.17E+06	4.85E+06	3.85E+06	7.63E+05	19.82	plasma	LDA
Unkown	level4f21	555.3969	2.0	1.48E+06	1.30E+06	8.76E+05	1.44E+06	1.44E+06	1.31E+06	2.50E+05	19.14	plasma	CD
Unkown	level4f22	562.3431	5.3	1.25E+08	1.44E+08	1.42E+08	1.30E+08	1.14E+08	1.31E+08	1.24E+07	9.47	plasma	LDA
Unkown	level4f22	562.3437	5.0	6.10E+06	1.25E+07	1.14E+07	1.36E+07	1.17E+07	1.11E+07	2.90E+06	26.22	plasma	XCMS
Unkown	level4f22	562.3431	5.3	1.50E+07	1.05E+07	1.49E+07	1.42E+07	1.21E+07	1.33E+07	1.96E+06	14.67	plasma	CD
Unkown	level4f23	562.3448	5.4	1.58E+08	1.37E+08	1.38E+08	1.34E+08	1.26E+08	1.38E+08	1.20E+07	8.67	plasma	LDA
Unkown	level4f23	562.3440	5.4	1.16E+07	1.22E+07	1.80E+07	2.07E+07	1.81E+07	1.61E+07	4.04E+06	25.05	plasma	XCMS
Unkown	level4f23	562.3448	5.4	6.26E+07	3.31E+07	4.49E+07	2.87E+07	3.33E+07	4.05E+07	1.37E+07	33.82	plasma	CD
Unkown	level4f24	569.3325	4.8	3.27E+07	2.30E+07	2.91E+07	2.99E+07	1.43E+07	2.58E+07	7.36E+06	28.51	plasma	LDA
Unkown	level4f24	569.3331	4.8	3.65E+06	4.30E+06	3.14E+06	3.57E+06	3.76E+06	3.68E+06	4.20E+05	11.40	plasma	XCMS
Unkown	level4f24	569.3325	4.8	7.53E+06	8.60E+06	6.32E+06	5.38E+06	4.52E+06	6.47E+06	1.63E+06	25.26	plasma	CD
Unkown	level4f25	575.3500	3.2	3.58E+06	2.97E+06	2.90E+06	3.28E+06	2.83E+06	3.11E+06	3.12E+05	10.03	plasma	LDA

Unkown	level4f25	575.3500	3.2	5.76E+05	7.42E+05	8.92E+05	5.44E+05	5.70E+05	6.65E+05	1.49E+05	22.43	plasma	CD
Unkown	level4f26	575.3511	3.8	7.30E+06	5.95E+06	5.42E+06	5.83E+06	4.83E+06	5.86E+06	9.13E+05	15.57	plasma	LDA
Unkown	level4f26	575.3510	3.8	1.93E+06	1.64E+06	1.46E+06	1.57E+06	1.27E+06	1.57E+06	2.40E+05	15.25	plasma	XCMS
Unkown	level4f26	575.3511	3.8	2.15E+06	2.67E+06	2.07E+06	1.87E+06	1.29E+06	2.01E+06	4.98E+05	24.79	plasma	CD
Unkown	level4f27	575.3967	2.0	1.22E+07	7.11E+06	5.86E+06	1.08E+07	1.59E+07	1.04E+07	4.02E+06	38.79	plasma	LDA
Unkown	level4f27	575.3967	2.0	4.29E+06	1.88E+06	2.35E+06	4.37E+06	6.39E+06	3.86E+06	1.81E+06	46.83	plasma	CD
Unkown	level4f28	575.8444	5.4	1.97E+07	8.76E+06	2.11E+07	1.88E+07	2.16E+07	1.80E+07	5.29E+06	29.38	plasma	LDA
Unkown	level4f28	575.8436	5.4	1.99E+06	2.26E+06	2.72E+06	3.73E+04	3.12E+06	2.03E+06	1.19E+06	58.84	plasma	XCMS
Unkown	level4f28	575.8444	5.4	5.66E+06	3.03E+06	5.48E+06	4.49E+06	6.40E+06	5.01E+06	1.30E+06	26.00	plasma	CD
Unkown	level4f29	575.8974	2.1	4.73E+06	4.37E+06	3.22E+06	4.10E+06	6.34E+06	4.55E+06	1.14E+06	25.07	plasma	LDA
Unkown	level4f29	575.8974	2.1	1.52E+06	1.14E+06	8.63E+05	1.34E+06	1.99E+06	1.37E+06	4.24E+05	30.91	plasma	CD
Unkown	level4f30	577.4111	2.0	2.52E+06	2.13E+06	2.40E+06	3.18E+06	4.02E+06	2.85E+06	7.59E+05	26.63	plasma	LDA
Unkown	level4f30	577.4111	2.0	9.29E+05	6.52E+05	8.12E+05	1.17E+06	1.53E+06	1.02E+06	3.43E+05	33.64	plasma	CD
Unkown	level4f31	583.3918	2.2	2.02E+06	1.02E+06	1.44E+06	1.43E+06	1.98E+06	1.58E+06	4.22E+05	26.71	plasma	LDA
Unkown	level4f31	583.3918	2.2	5.67E+05	1.59E+05	4.18E+05	5.41E+05	6.05E+05	4.58E+05	1.81E+05	39.57	plasma	CD
Unkown	level4f32	584.4011	2.3	1.16E+06	2.70E+06	3.08E+06	3.15E+06	4.16E+06	2.85E+06	1.09E+06	38.12	plasma	LDA
Unkown	level4f32	584.4001	2.4	7.05E+04	6.60E+05	3.89E+05	7.34E+05	9.99E+05	5.71E+05	3.54E+05	62.03	plasma	XCMS
Unkown	level4f32	584.4011	2.3	7.17E+05	1.01E+06	9.49E+05	1.25E+06	1.91E+06	1.17E+06	4.58E+05	39.19	plasma	CD
Unkown	level4f33	591.3458	4.8	1.01E+07	1.77E+07	2.36E+07	1.81E+07	1.57E+07	1.70E+07	4.85E+06	28.47	plasma	LDA
Unkown	level4f33	591.3458	4.8	9.19E+06	1.52E+07	1.21E+07	1.09E+07	3.82E+06	1.02E+07	4.20E+06	41.04	plasma	CD
Unkown	level4f34	120.0806	11.7	3.39E+07	1.36E+07	1.53E+07	1.81E+07	1.88E+07	1.99E+07	8.07E+06	40.47	plasma	LDA
Unkown	level4f34	120.0810	11.7	8.10E+06	3.73E+06	4.19E+06	5.46E+06	5.80E+06	5.46E+06	1.71E+06	31.35	plasma	CD
Unkown	level4f34	120.0809	11.7	7.51E+06	3.49E+06	3.85E+06	4.64E+06	4.76E+06	4.85E+06	1.58E+06	32.54	plasma	XCMS
Unkown	level4f35	597.4101	2.1	9.61E+06	6.23E+06	5.65E+06	1.04E+07	1.70E+07	9.79E+06	4.54E+06	46.44	plasma	LDA
Unkown	level4f35	597.4101	2.1	4.08E+06	2.31E+06	2.66E+06	2.81E+06	6.77E+06	3.73E+06	1.83E+06	49.07	plasma	CD
Unkown	level4f36	597.8564	5.3	3.19E+07	3.41E+07	9.26E+06	2.93E+07	1.12E+07	2.32E+07	1.19E+07	51.56	plasma	LDA
Unkown	level4f36	597.8566	5.5	3.43E+06	3.92E+06	3.80E+06	3.55E+06	3.59E+06	3.66E+06	1.99E+05	5.44	plasma	XCMS
Unkown	level4f36	597.8564	5.3	5.53E+06	6.07E+06	6.09E+06	5.06E+06	4.20E+06	5.39E+06	7.91E+05	14.68	plasma	CD
Unkown	level4f37	606.3699	5.3	7.95E+07	5.43E+07	4.74E+07	4.30E+07	3.45E+07	5.17E+07	1.71E+07	33.07	plasma	LDA
Unkown	level4f37	606.3704	5.1	2.76E+07	2.06E+07	1.89E+07	1.69E+07	1.01E+07	1.88E+07	6.33E+06	33.66	plasma	XCMS

Unkown	level4f37	606.3699	5.3	3.90E+07	2.35E+07	2.68E+07	2.53E+07	2.81E+07	2.85E+07	6.08E+06	21.32	plasma	CD
Unkown	level4f38	606.3715	5.5	7.29E+07	8.02E+07	1.03E+08	8.49E+07	8.72E+07	8.57E+07	1.13E+07	13.16	plasma	LDA
Unkown	level4f38	606.3704	5.1	2.76E+07	2.06E+07	1.89E+07	1.69E+07	1.01E+07	1.88E+07	6.33E+06	33.66	plasma	XCMS
Unkown	level4f38	606.3715	5.5	5.92E+07	4.21E+07	4.53E+07	4.61E+07	4.24E+07	4.70E+07	7.02E+06	14.92	plasma	CD
Unkown	level4f39	606.4139	2.4	4.40E+06	4.55E+06	3.59E+06	6.07E+06	7.42E+06	5.21E+06	1.53E+06	29.31	plasma	LDA
Unkown	level4f39	606.4136	2.4	1.06E+06	1.05E+06	8.52E+05	1.51E+06	1.92E+06	1.28E+06	4.33E+05	33.86	plasma	XCMS
Unkown	level4f39	606.4139	2.4	1.38E+06	2.11E+06	1.55E+06	2.53E+06	3.72E+06	2.26E+06	9.38E+05	41.55	plasma	CD
Unkown	level4f40	613.3593	4.9	3.66E+07	3.69E+07	2.45E+07	2.93E+07	2.74E+07	3.09E+07	5.55E+06	17.94	plasma	LDA
Unkown	level4f40	613.3593	5.4	1.93E+06	3.34E+06	3.45E+06	5.15E+06	2.83E+06	3.34E+06	1.18E+06	35.29	plasma	XCMS
Unkown	level4f40	613.3593	4.9	1.14E+07	7.42E+06	1.06E+07	7.24E+06	7.63E+06	8.86E+06	1.99E+06	22.43	plasma	CD
Unkown	level4f41	613.4027	2.4	3.27E+06	3.61E+06	2.75E+06	3.40E+06	4.02E+06	3.41E+06	4.65E+05	13.63	plasma	LDA
Unkown	level4f41	613.4027	2.4	1.41E+06	1.17E+06	1.03E+06	1.38E+06	1.19E+06	1.24E+06	1.61E+05	13.01	plasma	CD
Unkown	level4f42	619.8705	5.4	1.15E+07	3.09E+07	7.97E+06	2.49E+07	2.13E+07	1.93E+07	9.47E+06	49.03	plasma	LDA
Unkown	level4f42	619.8695	5.6	3.46E+06	3.02E+06	3.63E+06	2.71E+06	1.43E+06	2.85E+06	8.74E+05	30.66	plasma	XCMS
Unkown	level4f42	619.8705	5.4	1.43E+06	3.11E+06	3.18E+06	4.98E+06	2.63E+06	3.07E+06	1.28E+06	41.74	plasma	CD
Unkown	level4f43	628.3843	5.6	1.35E+08	1.04E+08	1.15E+08	1.32E+08	6.75E+07	1.11E+08	2.73E+07	24.64	plasma	LDA
Unkown	level4f43	628.3835	5.5	3.08E+07	2.47E+07	2.64E+07	2.82E+07	5.12E+07	3.22E+07	1.08E+07	33.57	plasma	XCMS
Unkown	level4f43	628.3843	5.6	6.09E+07	3.87E+07	5.36E+07	4.58E+07	6.08E+07	5.20E+07	9.69E+06	18.63	plasma	CD
Unkown	level4f44	628.3837	5.2	1.22E+08	8.80E+07	7.47E+07	6.97E+07	5.80E+07	8.25E+07	2.46E+07	29.79	plasma	LDA
Unkown	level4f44	628.3834	5.2	2.65E+07	1.79E+07	1.72E+07	1.86E+07	1.48E+07	1.90E+07	4.43E+06	23.32	plasma	XCMS
Unkown	level4f44	628.3837	5.2	3.12E+07	2.95E+07	5.36E+07	4.58E+07	3.28E+07	3.86E+07	1.06E+07	27.42	plasma	CD
Unkown	level4f45	628.4269	2.4	5.96E+06	7.29E+06	6.13E+06	9.47E+06	1.27E+07	8.32E+06	2.84E+06	34.16	plasma	LDA
Unkown	level4f45	628.4264	2.4	5.26E+04	1.81E+06	1.61E+06	2.39E+06	3.15E+06	1.80E+06	1.15E+06	63.69	plasma	XCMS
Unkown	level4f45	628.4269	2.4	2.42E+06	2.74E+06	2.78E+06	4.03E+06	4.52E+06	3.30E+06	9.16E+05	27.77	plasma	CD
Unkown	level4f46	628.9288	2.4	4.06E+06	4.35E+06	3.63E+06	5.19E+06	5.31E+06	4.50E+06	7.26E+05	16.12	plasma	LDA
Unkown	level4f46	628.9288	2.4	1.28E+06	1.27E+06	1.11E+06	1.39E+06	1.68E+06	1.35E+06	2.13E+05	15.81	plasma	CD
Unkown	level4f47	635.3719	4.9	8.12E+06	2.98E+07	3.25E+07	3.25E+07	2.15E+07	2.49E+07	1.04E+07	41.75	plasma	LDA
Unkown	level4f47	635.3719	4.9	3.12E+06	5.58E+06	4.41E+06	3.54E+06	2.92E+06	3.91E+06	1.09E+06	27.90	plasma	CD
Unkown	level4f48	635.4161	2.4	4.52E+06	4.31E+06	3.87E+06	4.19E+06	5.44E+06	4.47E+06	5.94E+05	13.30	plasma	LDA
Unkown	level4f48	635.4163	2.4	9.82E+05	9.61E+05	8.41E+05	4.35E+04	1.14E+06	7.94E+05	4.33E+05	54.52	plasma	XCMS

Unkown	level4f48	635.4161	2.4	2.13E+06	1.48E+06	1.80E+06	9.79E+05	2.41E+06	1.76E+06	5.57E+05	31.64	plasma	CD
Unkown	level4f49	641.4361	2.1	3.57E+06	2.49E+06	2.56E+06	3.43E+06	7.20E+06	3.85E+06	1.94E+06	50.31	plasma	LDA
Unkown	level4f49	641.4361	2.1	1.27E+06	1.22E+06	1.04E+06	1.38E+06	2.69E+06	1.52E+06	6.65E+05	43.78	plasma	CD
Unkown	level4f50	641.8824	5.5	7.68E+06	1.43E+07	1.50E+07	1.86E+07	1.39E+07	1.39E+07	3.93E+06	28.28	plasma	LDA
Unkown	level4f50	641.8824	5.6	2.66E+06	1.76E+06	1.07E+06	2.35E+06	9.70E+05	1.76E+06	7.50E+05	42.56	plasma	XCMS
Unkown	level4f50	641.8824	5.5	4.09E+06	4.79E+06	4.21E+06	3.07E+06	3.61E+06	3.95E+06	6.47E+05	16.38	plasma	CD
Unkown	level4f51	643.9964	1.7	3.20E+05	4.03E+05	4.41E+05	1.18E+06	2.48E+06	9.65E+05	9.15E+05	94.76	plasma	LDA
Unkown	level4f51	643.9964	1.7	1.22E+05	8.37E+04	1.45E+05	4.09E+05	8.62E+05	3.24E+05	3.27E+05	100.80	plasma	CD
Unkown	level4f52	650.3958	5.6	1.97E+08	2.28E+08	1.99E+08	2.01E+08	1.98E+08	2.05E+08	1.33E+07	6.48	plasma	LDA
Unkown	level4f52	650.3968	5.6	3.19E+07	3.05E+07	1.60E+07	2.56E+07	5.24E+07	3.13E+07	1.34E+07	42.73	plasma	XCMS
Unkown	level4f52	650.3958	5.6	3.96E+07	3.44E+07	1.78E+07	1.58E+07	1.02E+07	2.36E+07	1.27E+07	53.88	plasma	CD
Unkown	level4f53	650.3964	5.7	2.69E+08	2.40E+08	2.11E+08	2.11E+08	1.80E+08	2.22E+08	3.38E+07	15.21	plasma	LDA
Unkown	level4f53	650.3968	5.6	3.19E+07	3.05E+07	1.60E+07	2.56E+07	5.24E+07	3.13E+07	1.34E+07	42.73	plasma	XCMS
Unkown	level4f53	650.3964	5.7	6.34E+07	3.44E+07	6.59E+07	6.00E+07	4.94E+07	5.46E+07	1.29E+07	23.71	plasma	CD
Unkown	level4f54	650.4401	2.4	7.55E+06	8.90E+06	7.89E+06	1.13E+07	1.53E+07	1.02E+07	3.20E+06	31.46	plasma	LDA
Unkown	level4f54	650.4400	2.4	1.76E+06	2.16E+06	2.07E+06	2.59E+06	3.93E+06	2.50E+06	8.52E+05	34.02	plasma	XCMS
Unkown	level4f54	650.4401	2.4	3.03E+06	3.67E+06	3.49E+06	3.88E+06	6.26E+06	4.07E+06	1.27E+06	31.16	plasma	CD
Unkown	level4f55	131.0532	20.8	5.77E+06	7.48E+06	7.25E+06	7.80E+06	6.99E+06	7.06E+06	7.77E+05	11.01	plasma	LDA
Unkown	level4f55	131.0532	20.4	1.83E+06	2.32E+06	2.04E+06	1.98E+06	1.52E+06	1.94E+06	2.91E+05	15.02	plasma	CD
Unkown	level4f55	131.0534	20.3	1.63E+06	2.08E+06	1.86E+06	1.83E+06	9.58E+05	1.67E+06	4.29E+05	25.69	plasma	XCMS
Unkown	level4f56	657.4294	2.4	7.66E+05	5.08E+06	3.91E+06	4.42E+06	5.63E+06	3.96E+06	1.90E+06	47.99	plasma	LDA
Unkown	level4f56	657.4293	2.5	9.37E+05	1.08E+06	8.87E+05	9.20E+05	9.81E+05	9.61E+05	7.47E+04	7.78	plasma	XCMS
Unkown	level4f56	657.4294	2.4	1.68E+06	1.87E+06	1.29E+06	1.75E+06	2.32E+06	1.78E+06	3.69E+05	20.69	plasma	CD
Unkown	level4f57	134.0190	16.3	6.91E+06	1.02E+07	8.06E+06	5.62E+06	5.39E+06	7.24E+06	1.99E+06	27.48	plasma	LDA
Unkown	level4f57	134.0190	16.2	1.76E+06	2.67E+06	2.09E+06	1.46E+06	1.38E+06	1.87E+06	5.27E+05	28.16	plasma	CD
Unkown	level4f57	134.0190	16.2	1.64E+06	2.49E+06	1.94E+06	1.36E+06	1.24E+06	1.73E+06	5.00E+05	28.86	plasma	XCMS
Unkown	level4f58	672.4095	5.7	1.16E+08	1.08E+08	8.91E+07	1.12E+08	7.26E+07	9.97E+07	1.84E+07	18.44	plasma	LDA
Unkown	level4f58	672.4095	5.7	2.19E+07	2.10E+07	2.59E+07	1.89E+07	2.21E+07	2.19E+07	2.54E+06	11.59	plasma	CD
Unkown	level4f58	672.4097	5.7	2.29E+07	2.21E+07	4.96E+07	4.82E+07	2.01E+07	3.26E+07	1.50E+07	45.94	plasma	XCMS
Unkown	level4f59	672.4095	5.3	7.07E+07	7.38E+07	6.52E+07	6.73E+07	6.40E+07	6.82E+07	4.04E+06	5.92	plasma	LDA

Unkown	level4f59	672.4096	5.2	1.73E+07	1.17E+07	1.25E+07	1.40E+07	1.13E+07	1.34E+07	2.45E+06	18.32	plasma	XCMS
Unkown	level4f59	672.4095	5.3	6.89E+07	5.20E+07	2.59E+07	4.54E+07	4.03E+07	4.65E+07	1.58E+07	33.91	plasma	CD
Unkown	level4f60	672.4530	2.4	7.59E+06	9.78E+06	9.73E+06	1.09E+07	1.49E+07	1.06E+07	2.69E+06	25.45	plasma	LDA
Unkown	level4f60	672.4531	2.5	1.75E+06	1.66E+06	2.40E+06	2.86E+06	3.75E+06	2.49E+06	8.59E+05	34.57	plasma	XCMS
Unkown	level4f60	672.4530	2.4	3.21E+06	3.91E+06	3.25E+06	4.56E+06	1.15E+06	3.22E+06	1.28E+06	39.85	plasma	CD
Unkown	level4f61	679.4428	2.4	3.18E+05	4.09E+06	4.37E+06	5.74E+06	5.68E+06	4.04E+06	2.21E+06	54.71	plasma	LDA
Unkown	level4f61	679.4428	2.4	1.28E+06	1.82E+06	1.42E+06	2.08E+06	2.75E+06	1.87E+06	5.85E+05	31.29	plasma	CD
Unkown	level4f63	138.9066	11.5	2.81E+07	3.58E+07	3.21E+07	2.92E+07	3.06E+07	3.12E+07	3.00E+06	9.64	plasma	LDA
Unkown	level4f63	138.9067	11.5	9.71E+06	1.39E+07	1.19E+07	1.04E+07	1.17E+07	1.15E+07	1.63E+06	14.11	plasma	CD
Unkown	level4f63	138.9067	11.5	6.76E+06	8.63E+06	7.08E+06	6.31E+06	7.33E+06	7.22E+06	8.76E+05	12.12	plasma	XCMS
Unkown	level4f64	694.4235	5.8	1.29E+08	5.83E+07	4.82E+07	7.29E+07	6.84E+07	7.53E+07	3.13E+07	41.60	plasma	LDA
Unkown	level4f64	694.4235	5.8	5.47E+07	4.69E+07	2.50E+07	3.93E+07	3.27E+07	3.97E+07	1.16E+07	29.32	plasma	CD
Unkown	level4f64	694.4228	5.7	2.84E+07	1.55E+07	1.15E+07	1.65E+07	1.97E+07	1.83E+07	6.34E+06	34.61	plasma	XCMS
Unkown	level4f65	694.4235	5.4	4.44E+07	4.16E+07	3.42E+07	2.26E+07	2.46E+07	3.35E+07	9.78E+06	29.20	plasma	LDA
Unkown	level4f65	694.4226	5.2	9.65E+06	1.10E+07	8.19E+06	1.01E+07	7.34E+06	9.25E+06	1.46E+06	15.82	plasma	XCMS
Unkown	level4f65	694.4235	5.4	1.42E+07	1.53E+07	2.52E+07	2.00E+07	2.98E+07	2.09E+07	6.60E+06	31.53	plasma	CD
Unkown	level4f66	140.0686	12.2	1.53E+08	1.43E+08	1.51E+08	1.39E+08	1.37E+08	1.45E+08	7.17E+06	4.96	plasma	LDA
Unkown	level4f66	140.0684	12.2	3.87E+07	3.53E+07	3.77E+07	3.39E+07	3.41E+07	3.59E+07	2.15E+06	5.98	plasma	CD
Unkown	level4f66	140.0684	12.2	3.49E+07	3.27E+07	3.47E+07	2.61E+07	3.16E+07	3.20E+07	3.55E+06	11.10	plasma	XCMS
Unkown	level4f67	701.4560	2.5	3.34E+06	3.67E+06	4.13E+06	4.07E+06	5.85E+06	4.21E+06	9.67E+05	22.97	plasma	LDA
Unkown	level4f67	701.4545	2.5	7.63E+05	7.90E+05	2.81E+05	5.64E+05	8.60E+05	6.52E+05	2.34E+05	35.97	plasma	XCMS
Unkown	level4f67	701.4560	2.5	1.05E+06	9.80E+05	1.76E+06	1.82E+06	1.47E+06	1.42E+06	3.92E+05	27.66	plasma	CD
Unkown	level4f68	701.9556	2.5	2.86E+06	2.93E+06	3.28E+06	3.74E+06	4.02E+06	3.37E+06	5.04E+05	14.97	plasma	LDA
Unkown	level4f68	701.9556	2.5	6.66E+05	1.19E+06	1.21E+06	9.21E+05	1.28E+06	1.05E+06	2.56E+05	24.32	plasma	CD
Unkown	level4f69	144.0821	17.8	8.04E+06	7.83E+06	9.90E+06	9.22E+06	6.95E+06	8.39E+06	1.17E+06	13.96	plasma	LDA
Unkown	level4f69	144.0820	17.7	9.13E+05	1.90E+06	2.05E+06	1.85E+06	5.94E+05	1.46E+06	6.60E+05	45.15	plasma	CD
Unkown	level4f69	144.0819	17.7	1.61E+06	6.68E+05	1.98E+06	1.74E+06	9.26E+03	1.20E+06	8.30E+05	69.22	plasma	XCMS
Unkown	level4f70	716.4363	5.7	1.19E+08	1.07E+08	9.21E+07	3.31E+07	2.33E+07	7.50E+07	4.39E+07	58.55	plasma	LDA
Unkown	level4f70	716.4357	5.8	1.80E+07	1.31E+07	2.72E+07	2.77E+07	1.05E+07	1.93E+07	7.93E+06	41.07	plasma	XCMS
Unkown	level4f70	716.4363	5.7	3.39E+07	2.96E+07	2.51E+07	3.18E+07	2.52E+07	2.91E+07	3.91E+06	13.43	plasma	CD

Unkown	level4f71	716.4806	2.5	8.37E+06	8.51E+06	8.34E+06	1.05E+07	1.45E+07	1.00E+07	2.66E+06	26.49	plasma	LDA
Unkown	level4f71	716.4791	2.5	1.75E+06	2.04E+06	4.32E+05	2.52E+06	3.47E+06	2.04E+06	1.11E+06	54.45	plasma	XCMS
Unkown	level4f71	716.4806	2.5	3.89E+06	4.11E+06	3.44E+06	3.95E+06	6.31E+06	4.34E+06	1.13E+06	25.99	plasma	CD
Unkown	level4f72	716.9816	2.5	5.01E+06	5.14E+06	5.46E+06	7.98E+06	1.08E+07	6.88E+06	2.51E+06	36.51	plasma	LDA
Unkown	level4f72	716.9816	2.5	1.84E+06	1.73E+06	1.57E+06	2.24E+06	3.28E+06	2.13E+06	6.89E+05	32.27	plasma	CD
Unkown	level4f73	717.4373	6.2	7.59E+06	9.98E+06	6.28E+06	1.04E+07	8.07E+06	8.47E+06	1.72E+06	20.32	plasma	LDA
Unkown	level4f73	717.4378	5.8	1.44E+06	1.81E+06	4.00E+06	1.54E+06	1.62E+06	2.08E+06	1.08E+06	51.81	plasma	XCMS
Unkown	level4f73	717.4373	6.2	2.35E+06	2.04E+06	1.17E+06	2.36E+06	1.43E+06	1.87E+06	5.46E+05	29.16	plasma	CD
Unkown	level4f74	723.4690	2.5	3.19E+06	3.13E+06	3.53E+06	4.08E+06	4.70E+06	3.73E+06	6.61E+05	17.73	plasma	LDA
Unkown	level4f74	723.4690	2.5	1.15E+06	7.49E+05	1.18E+06	1.51E+06	1.50E+06	1.21E+06	3.11E+05	25.64	plasma	CD
Unkown	level4f75	148.0042	14.4	1.41E+07	1.40E+07	1.43E+07	1.41E+07	1.19E+07	1.37E+07	1.01E+06	7.34	plasma	LDA
Unkown	level4f75	148.0045	14.4	1.07E+07	1.16E+07	1.13E+07	1.09E+07	9.13E+06	1.07E+07	9.63E+05	8.96	plasma	CD
Unkown	level4f75	148.0041	14.4	2.74E+06	3.38E+06	3.41E+06	3.27E+06	2.86E+06	3.13E+06	3.13E+05	9.98	plasma	XCMS
Unkown	level4f76	148.0798	20.4	2.87E+07	3.14E+07	2.80E+07	2.88E+07	2.12E+07	2.76E+07	3.83E+06	13.88	plasma	LDA
Unkown	level4f76	148.0798	20.5	1.88E+06	8.06E+06	6.58E+06	6.93E+06	4.62E+06	5.61E+06	2.43E+06	43.27	plasma	CD
Unkown	level4f76	148.0799	20.3	5.96E+06	7.18E+06	6.40E+06	6.80E+06	3.31E+06	5.93E+06	1.53E+06	25.82	plasma	XCMS
Unkown	level4f77	148.9356	11.6	1.45E+07	1.76E+07	1.55E+07	1.46E+07	1.65E+07	1.57E+07	1.32E+06	8.42	plasma	LDA
Unkown	level4f77	148.9354	11.5	4.74E+06	4.41E+06	3.93E+06	3.93E+06	5.98E+06	4.60E+06	8.44E+05	18.36	plasma	CD
Unkown	level4f77	148.9354	11.5	3.59E+06	4.53E+06	3.82E+06	3.75E+06	4.32E+06	4.00E+06	4.04E+05	10.11	plasma	XCMS
Unkown	level4f78	738.4487	5.7	7.53E+07	7.03E+07	2.18E+07	6.36E+07	1.42E+07	4.90E+07	2.87E+07	58.60	plasma	LDA
Unkown	level4f78	738.4488	5.8	1.21E+07	7.42E+06	6.52E+06	7.77E+06	7.93E+06	8.35E+06	2.17E+06	26.02	plasma	XCMS
Unkown	level4f78	738.4487	5.7	2.37E+07	1.48E+07	1.28E+07	1.77E+07	1.53E+07	1.68E+07	4.20E+06	24.96	plasma	CD
Unkown	level4f79	738.4934	2.5	7.31E+06	8.87E+06	7.48E+06	1.09E+07	1.47E+07	9.84E+06	3.06E+06	31.07	plasma	LDA
Unkown	level4f79	738.4934	2.5	3.58E+06	3.50E+06	3.28E+06	4.45E+06	6.43E+06	4.25E+06	1.30E+06	30.56	plasma	CD
Unkown	level4f80	745.4833	2.5	2.71E+06	2.96E+06	2.47E+06	2.91E+06	3.49E+06	2.91E+06	3.78E+05	12.99	plasma	LDA
Unkown	level4f80	745.4833	2.5	2.37E+07	1.48E+07	1.28E+07	1.77E+07	1.53E+07	1.68E+07	4.20E+06	24.96	plasma	CD
Unkown	level4f81	151.0970	3.0	6.24E+07	5.18E+07	5.03E+07	5.39E+07	5.79E+07	5.53E+07	4.92E+06	8.91	plasma	LDA
Unkown	level4f81	151.0969	2.9	1.15E+07	1.81E+07	1.53E+07	1.14E+07	2.07E+07	1.54E+07	4.08E+06	26.48	plasma	CD
Unkown	level4f81	151.0967	2.9	1.02E+07	6.83E+06	6.12E+06	8.13E+06	7.98E+06	7.86E+06	1.57E+06	20.00	plasma	XCMS
Unkown	level4f82	151.1448	7.4	3.92E+07	2.71E+07	3.19E+07	3.23E+07	2.72E+07	3.16E+07	4.94E+06	15.64	plasma	LDA

Unkown	level4f82	151.1446	7.4	9.14E+06	6.62E+06	7.51E+06	7.67E+06	6.35E+06	7.46E+06	1.10E+06	14.69	plasma	CD
Unkown	level4f82	151.1444	7.3	6.09E+04	8.07E+04	6.35E+04	7.82E+06	6.58E+06	2.92E+06	3.93E+06	134.57	plasma	XCMS
Unkown	level4f83	760.4615	5.9	3.41E+07	3.53E+07	9.41E+06	3.56E+07	3.13E+07	2.91E+07	1.12E+07	38.29	plasma	LDA
Unkown	level4f83	760.4613	5.9	4.65E+06	3.39E+06	3.75E+06	3.73E+06	3.83E+06	3.87E+06	4.67E+05	12.06	plasma	XCMS
Unkown	level4f83	760.4615	5.9	1.01E+07	9.55E+06	7.23E+06	9.33E+06	8.52E+06	8.95E+06	1.12E+06	12.47	plasma	CD
Unkown	level4f84	760.5067	2.5	5.67E+06	6.93E+06	8.80E+06	9.94E+06	1.15E+07	8.56E+06	2.32E+06	27.07	plasma	LDA
Unkown	level4f84	760.5067	2.5	2.51E+06	2.83E+06	3.54E+06	3.87E+06	5.46E+06	3.64E+06	1.15E+06	31.68	plasma	CD
Unkown	level4f85	767.4955	2.5	1.76E+06	1.64E+06	2.18E+06	3.07E+06	2.75E+06	2.28E+06	6.21E+05	27.23	plasma	LDA
Unkown	level4f85	767.4955	2.5	8.80E+05	6.91E+05	9.93E+05	1.35E+06	1.28E+06	1.04E+06	2.74E+05	26.38	plasma	CD
Unkown	level4f86	155.1544	4.8	1.18E+08	8.39E+07	8.03E+07	1.08E+08	1.23E+08	1.03E+08	1.95E+07	19.01	plasma	LDA
Unkown	level4f86	155.1545	4.8	2.91E+07	2.09E+07	2.00E+07	2.77E+07	3.27E+07	2.61E+07	5.49E+06	21.05	plasma	CD
Unkown	level4f86	155.1544	4.8	2.83E+07	2.03E+07	1.96E+07	2.58E+07	2.98E+07	2.48E+07	4.66E+06	18.81	plasma	XCMS
Unkown	level4f87	156.0683	10.3	3.19E+06	2.29E+06	2.04E+06	2.00E+06	3.08E+06	2.52E+06	5.71E+05	22.66	plasma	LDA
Unkown	level4f87	156.0683	10.3	8.00E+05	5.92E+05	5.76E+05	5.36E+05	7.74E+05	6.56E+05	1.22E+05	18.57	plasma	CD
Unkown	level4f87	156.0685	10.2	8.02E+05	5.51E+05	5.20E+05	5.03E+05	7.66E+05	6.28E+05	1.44E+05	22.88	plasma	XCMS
Unkown	level4f88	782.4736	6.0	2.01E+07	1.49E+07	1.35E+07	9.89E+06	1.18E+07	1.40E+07	3.86E+06	27.52	plasma	LDA
Unkown	level4f88	782.4736	6.0	4.60E+06	3.39E+06	4.25E+06	4.62E+06	5.59E+06	4.49E+06	7.91E+05	17.60	plasma	CD
Unkown	level4f89	782.5193	2.5	4.13E+06	4.23E+06	5.07E+06	6.43E+06	9.10E+06	5.79E+06	2.06E+06	35.66	plasma	LDA
Unkown	level4f89	782.5193	2.5	1.68E+06	1.70E+06	2.16E+06	2.74E+06	3.67E+06	2.39E+06	8.36E+05	34.96	plasma	CD
Unkown	level4f90	158.9645	13.5	5.18E+08	5.96E+08	6.56E+08	4.77E+08	5.01E+08	5.50E+08	7.41E+07	13.48	plasma	LDA
Unkown	level4f90	158.9644	13.6	3.11E+07	8.07E+06	2.18E+07	4.26E+07	5.05E+07	3.08E+07	1.68E+07	54.44	plasma	CD
Unkown	level4f90	158.9641	13.1	3.70E+07	3.48E+07	2.21E+07	3.15E+07	4.66E+07	3.44E+07	8.85E+06	25.73	plasma	XCMS
Unkown	level4f91	789.5076	2.5	1.07E+06	1.37E+06	1.20E+06	1.92E+06	1.55E+06	1.42E+06	3.32E+05	23.32	plasma	LDA
Unkown	level4f91	789.5076	2.5	3.88E+05	4.29E+05	4.71E+05	5.83E+05	7.50E+05	5.24E+05	1.46E+05	27.84	plasma	CD
Unkown	level4f92	160.0349	14.1	6.45E+07	5.61E+07	6.29E+07	5.58E+07	5.32E+07	5.85E+07	4.93E+06	8.43	plasma	LDA
Unkown	level4f92	160.0348	14.0	1.57E+07	1.11E+07	1.54E+07	1.28E+07	5.54E+06	1.21E+07	4.14E+06	34.20	plasma	CD
Unkown	level4f92	160.0347	14.0	1.49E+07	8.68E+04	1.45E+07	1.33E+07	9.64E+06	1.05E+07	6.18E+06	58.87	plasma	XCMS
Unkown	level4f93	160.0767	18.4	2.73E+06	5.25E+06	2.14E+06	6.69E+05	1.70E+05	2.19E+06	2.00E+06	91.37	plasma	LDA
Unkown	level4f93	160.0767	18.3	6.69E+05	1.22E+06	5.19E+05	1.72E+05	4.42E+04	5.25E+05	4.65E+05	88.44	plasma	CD
Unkown	level4f93	160.0768	18.3	6.70E+05	1.02E+06	5.11E+05	1.64E+05	4.52E+04	4.82E+05	3.93E+05	81.56	plasma	XCMS

Unkown	level4f94	162.0500	13.6	2.55E+07	2.45E+07	2.44E+07	2.35E+07	2.18E+07	2.39E+07	1.42E+06	5.93	plasma	LDA
Unkown	level4f94	162.0500	13.6	6.83E+06	6.03E+06	6.33E+06	5.84E+06	5.00E+06	6.01E+06	6.74E+05	11.21	plasma	CD
Unkown	level4f94	162.0499	13.5	5.74E+04	5.68E+06	5.72E+06	5.60E+06	4.76E+06	4.37E+06	2.44E+06	55.90	plasma	XCMS
Unkown	level4f95	162.1099	8.8	1.05E+09	1.14E+09	1.07E+09	9.83E+08	9.89E+08	1.05E+09	6.24E+07	5.97	plasma	LDA
Unkown	level4f95	162.1097	8.7	2.40E+06	2.84E+06	2.28E+06	2.19E+06	2.17E+06	2.38E+06	2.75E+05	11.58	plasma	CD
Unkown	level4f95	162.1000	8.7	2.65E+05	1.94E+05	2.88E+05	1.87E+05	1.83E+05	2.23E+05	4.94E+04	22.12	plasma	XCMS
Unkown	level4f96	163.0562	17.8	1.58E+07	1.34E+07	1.25E+07	1.52E+07	1.36E+07	1.41E+07	1.39E+06	9.83	plasma	LDA
Unkown	level4f96	163.0565	17.7	4.02E+06	2.29E+06	2.69E+06	2.49E+06	3.27E+06	2.95E+06	7.03E+05	23.83	plasma	CD
Unkown	level4f96	163.0566	17.7	3.72E+06	1.96E+06	2.06E+06	4.58E+03	1.58E+06	1.86E+06	1.33E+06	71.14	plasma	XCMS
Unkown	level4f97	165.0700	2.1	7.50E+06	8.35E+06	5.45E+06	9.44E+06	8.25E+06	7.80E+06	1.48E+06	19.02	plasma	LDA
Unkown	level4f97	165.0700	2.5	4.18E+06	3.79E+06	4.41E+06	4.20E+06	4.05E+06	4.13E+06	2.28E+05	5.51	plasma	CD
Unkown	level4f98	169.0586	20.5	1.72E+07	1.93E+07	1.90E+07	1.76E+07	1.69E+07	1.80E+07	1.09E+06	6.04	plasma	LDA
Unkown	level4f98	169.0585	20.4	4.05E+06	5.22E+06	1.89E+06	4.58E+06	3.59E+06	3.87E+06	1.26E+06	32.55	plasma	CD
Unkown	level4f98	169.0585	20.4	3.92E+06	4.37E+06	4.63E+06	3.06E+06	1.64E+06	3.52E+06	1.21E+06	34.30	plasma	XCMS
Unkown	level4f99	169.9860	14.5	3.14E+07	3.22E+07	3.00E+07	2.97E+07	3.19E+07	3.10E+07	1.13E+06	3.63	plasma	LDA
Unkown	level4f99	169.9861	14.4	6.58E+06	7.45E+06	7.06E+06	6.86E+06	6.17E+06	6.82E+06	4.85E+05	7.10	plasma	CD
Unkown	level4f99	169.9862	14.4	5.32E+04	7.16E+06	6.99E+06	6.87E+06	6.31E+06	5.48E+06	3.05E+06	55.66	plasma	XCMS
Unkown	level4f100	171.0996	13.5	9.54E+07	5.71E+07	5.39E+07	7.17E+07	7.40E+07	7.04E+07	1.65E+07	23.46	plasma	LDA
Unkown	level4f100	171.0996	13.6	3.48E+06	2.34E+06	6.35E+06	3.62E+06	7.11E+06	4.58E+06	2.04E+06	44.54	plasma	CD
Unkown	level4f101	173.0300	14.8	4.50E+06	3.85E+06	3.88E+06	2.99E+06	2.44E+06	3.53E+06	8.14E+05	23.05	plasma	LDA
Unkown	level4f101	173.0300	14.7	1.11E+06	9.35E+05	9.75E+05	7.52E+05	5.99E+05	8.74E+05	1.99E+05	22.79	plasma	CD
Unkown	level4f101	173.0300	14.7	1.07E+06	9.21E+05	9.15E+05	7.15E+05	5.86E+05	8.42E+05	1.91E+05	22.69	plasma	XCMS
Unkown	level4f102	173.0710	10.3	7.54E+06	6.63E+06	8.20E+06	8.02E+06	9.58E+06	7.99E+06	1.08E+06	13.45	plasma	LDA
Unkown	level4f102	173.0716	10.3	1.25E+07	1.58E+07	1.50E+07	1.32E+07	1.47E+07	1.42E+07	1.37E+06	9.64	plasma	CD
Unkown	level4f102	173.0712	10.3	1.99E+06	1.67E+06	2.11E+06	1.42E+04	2.03E+06	1.56E+06	8.82E+05	56.43	plasma	XCMS
Unkown	level4f103	176.1223	31.0	9.29E+06	8.94E+06	1.06E+07	9.50E+06	9.63E+06	9.60E+06	6.31E+05	6.58	plasma	LDA
Unkown	level4f103	176.1223	30.5	1.02E+06	2.77E+06	2.35E+06	2.30E+06	2.15E+06	2.12E+06	6.55E+05	30.95	plasma	CD
Unkown	level4f104	180.0813	2.2	2.65E+07	2.47E+07	2.60E+07	2.77E+07	2.85E+07	2.67E+07	1.51E+06	5.64	plasma	LDA
Unkown	level4f104	180.0813	2.3	6.29E+06	5.75E+06	6.94E+06	5.86E+06	7.79E+06	6.52E+06	8.48E+05	13.00	plasma	CD
Unkown	level4f104	180.0811	2.3	6.87E+06	6.25E+06	6.39E+06	7.09E+06	7.40E+06	6.80E+06	4.79E+05	7.05	plasma	XCMS

Unkown	level4f105	185.1146	13.4	1.88E+07	4.55E+07	2.73E+07	2.33E+07	3.01E+07	2.90E+07	1.02E+07	35.07	plasma	LDA
Unkown	level4f105	185.1146	13.4	2.57E+06	3.04E+06	2.13E+06	1.36E+05	2.19E+06	2.01E+06	1.11E+06	55.17	plasma	CD
Unkown	level4f106	196.8649	11.5	2.38E+07	3.42E+07	3.24E+07	3.05E+07	2.99E+07	3.02E+07	3.96E+06	13.11	plasma	LDA
Unkown	level4f106	196.8653	11.5	6.26E+06	7.54E+06	8.50E+06	1.43E+07	1.49E+07	1.03E+07	4.02E+06	38.99	plasma	CD
Unkown	level4f106	196.8654	11.5	5.77E+06	8.36E+06	6.70E+06	6.50E+06	7.15E+06	6.90E+06	9.57E+05	13.87	plasma	XCMS
Unkown	level4f107	198.8624	11.5	2.34E+07	3.35E+07	3.17E+07	3.02E+07	2.94E+07	2.96E+07	3.84E+06	12.95	plasma	LDA
Unkown	level4f107	198.8624	11.5	5.64E+06	6.00E+06	7.82E+06	7.98E+06	7.55E+06	7.00E+06	1.09E+06	15.63	plasma	CD
Unkown	level4f107	198.8624	11.5	5.72E+06	8.18E+06	6.91E+06	6.48E+06	7.03E+06	6.86E+06	8.95E+05	13.04	plasma	XCMS
Unkown	level4f108	203.0529	14.2	1.49E+07	1.77E+07	4.77E+07	1.90E+07	1.60E+07	2.31E+07	1.39E+07	60.18	plasma	LDA
Unkown	level4f108	203.0528	14.6	6.41E+06	6.32E+06	6.63E+06	5.51E+06	5.79E+06	6.13E+06	4.63E+05	7.55	plasma	CD
Unkown	level4f108	203.0529	14.6	5.97E+06	6.60E+06	6.70E+06	5.83E+06	4.83E+06	5.99E+06	7.50E+05	12.52	plasma	XCMS
Unkown	level4f109	204.0817	7.1	4.62E+06	6.12E+06	5.83E+06	6.75E+06	2.97E+06	5.26E+06	1.49E+06	28.41	plasma	LDA
Unkown	level4f109	204.0817	7.1	3.54E+05	1.00E+06	7.95E+05	9.55E+05	1.84E+05	6.58E+05	3.68E+05	55.92	plasma	CD
Unkown	level4f109	204.0816	7.0	3.32E+05	8.87E+05	7.56E+05	8.62E+05	1.94E+05	6.06E+05	3.21E+05	52.95	plasma	XCMS
Unkown	level4f110	204.1656	7.1	4.49E+06	6.09E+06	5.44E+06	6.79E+06	3.24E+06	5.21E+06	1.39E+06	26.72	plasma	LDA
Unkown	level4f110	204.1656	7.1	3.54E+05	9.84E+05	8.33E+05	9.32E+05	1.91E+05	6.59E+05	3.62E+05	54.88	plasma	CD
Unkown	level4f110	204.1655	7.0	3.41E+05	8.62E+05	7.63E+05	8.53E+05	1.95E+05	6.03E+05	3.12E+05	51.81	plasma	XCMS
Unkown	level4f111	206.0484	1.6	3.21E+06	2.73E+06	2.84E+06	2.65E+06	2.04E+06	2.69E+06	4.23E+05	15.71	plasma	LDA
Unkown	level4f111	206.0483	1.6	2.04E+06	1.64E+06	1.66E+06	1.58E+06	1.49E+06	1.68E+06	2.09E+05	12.44	plasma	CD
Unkown	level4f112	206.8936	11.5	2.65E+07	3.74E+07	3.66E+07	3.53E+07	3.40E+07	3.40E+07	4.33E+06	12.75	plasma	LDA
Unkown	level4f112	206.8942	11.5	1.09E+07	1.72E+07	1.24E+07	1.32E+07	1.50E+07	1.37E+07	2.44E+06	17.73	plasma	CD
Unkown	level4f112	206.8942	11.5	6.48E+06	9.22E+06	8.62E+06	8.23E+06	8.49E+06	8.21E+06	1.03E+06	12.55	plasma	XCMS
Unkown	level4f113	210.0498	11.7	1.57E+07	3.81E+06	5.00E+06	7.24E+06	6.68E+06	7.69E+06	4.68E+06	60.89	plasma	LDA
Unkown	level4f113	210.0499	11.7	3.98E+06	1.05E+06	1.30E+06	1.95E+06	1.75E+06	2.01E+06	1.16E+06	57.95	plasma	CD
Unkown	level4f113	210.0504	11.7	3.70E+06	9.68E+05	1.21E+06	1.77E+06	1.63E+06	1.85E+06	1.08E+06	58.13	plasma	XCMS
Unkown	level4f114	212.9993	13.6	1.44E+07	1.36E+07	1.50E+07	1.42E+07	1.32E+07	1.41E+07	7.15E+05	5.07	plasma	LDA
Unkown	level4f114	212.9994	13.5	8.10E+06	7.97E+06	8.14E+06	8.54E+06	6.60E+06	7.87E+06	7.44E+05	9.45	plasma	CD
Unkown	level4f114	212.9994	13.6	3.60E+06	3.29E+06	3.61E+06	3.67E+06	3.14E+06	3.46E+06	2.33E+05	6.73	plasma	XCMS
Unkown	level4f115	219.0801	3.0	1.28E+08	1.40E+08	1.05E+08	1.05E+08	1.01E+08	1.16E+08	1.73E+07	14.87	plasma	LDA
Unkown	level4f115	219.0804	2.9	7.80E+06	2.73E+04	2.15E+07	1.83E+07	1.95E+07	1.34E+07	9.18E+06	68.37	plasma	CD

Unkown	level4f115	219.0802	3.0	1.28E+07	1.70E+07	1.69E+07	1.24E+07	1.13E+07	1.41E+07	2.68E+06	19.01	plasma	XCMS
Unkown	level4f116	226.9514	13.6	6.92E+08	1.11E+09	9.83E+08	8.70E+08	9.63E+08	9.23E+08	1.54E+08	16.72	plasma	LDA
Unkown	level4f116	226.9517	13.5	1.68E+08	1.22E+08	9.83E+07	6.93E+07	1.21E+08	1.16E+08	3.63E+07	31.40	plasma	CD
Unkown	level4f117	228.0224	14.1	8.02E+06	6.90E+06	8.06E+06	7.43E+06	6.21E+06	7.33E+06	7.86E+05	10.74	plasma	LDA
Unkown	level4f117	228.0224	14.0	2.03E+06	1.09E+06	1.97E+06	1.88E+06	1.73E+06	1.74E+06	3.82E+05	21.99	plasma	CD
Unkown	level4f117	228.0221	14.0	1.96E+06	1.29E+06	1.97E+06	1.82E+06	1.79E+04	1.41E+06	8.28E+05	58.65	plasma	XCMS
Unkown	level4f118	237.0432	10.3	4.80E+06	3.95E+06	3.76E+06	3.40E+06	5.10E+06	4.20E+06	7.18E+05	17.09	plasma	LDA
Unkown	level4f118	237.0429	10.2	1.30E+06	1.10E+06	1.15E+06	9.72E+05	1.36E+06	1.18E+06	1.56E+05	13.25	plasma	CD
Unkown	level4f119	237.9735	14.4	6.17E+06	7.11E+06	6.99E+06	6.82E+06	5.82E+06	6.58E+06	5.63E+05	8.55	plasma	LDA
Unkown	level4f119	237.9735	14.4	1.48E+06	1.69E+06	1.72E+06	1.61E+06	1.38E+06	1.58E+06	1.40E+05	8.92	plasma	CD
Unkown	level4f119	237.9738	14.4	9.84E+03	1.51E+06	1.69E+06	1.66E+06	1.41E+06	1.26E+06	7.07E+05	56.21	plasma	XCMS
Unkown	level4f120	239.1492	2.6	1.40E+08	1.35E+08	1.26E+08	1.33E+08	1.32E+08	1.33E+08	5.19E+06	3.89	plasma	LDA
Unkown	level4f120	239.1491	2.7	1.35E+07	1.94E+07	3.16E+07	2.12E+07	3.51E+07	2.41E+07	8.95E+06	37.08	plasma	CD
Unkown	level4f120	239.1496	2.6	1.48E+07	1.63E+07	1.36E+07	1.79E+07	1.46E+07	1.54E+07	1.69E+06	10.96	plasma	XCMS
Unkown	level4f121	240.1023	8.7	2.55E+06	4.66E+06	3.61E+06	2.82E+06	2.95E+06	3.32E+06	8.46E+05	25.50	plasma	LDA
Unkown	level4f121	240.1023	8.6	6.35E+05	1.15E+06	9.40E+05	7.20E+05	6.92E+05	8.27E+05	2.13E+05	25.80	plasma	CD
Unkown	level4f122	240.1711	6.4	2.91E+07	3.09E+07	2.99E+07	3.99E+07	3.68E+07	3.33E+07	4.76E+06	14.29	plasma	LDA
Unkown	level4f122	240.1711	6.5	7.38E+06	8.24E+06	8.09E+06	1.02E+07	9.68E+06	8.72E+06	1.18E+06	13.54	plasma	CD
Unkown	level4f122	240.1710	6.4	7.46E+06	7.69E+06	7.75E+06	9.93E+06	7.26E+06	8.02E+06	1.09E+06	13.54	plasma	XCMS
Unkown	level4f123	245.2266	1.9	1.03E+07	1.12E+07	1.07E+07	1.31E+07	1.50E+07	1.21E+07	1.97E+06	16.36	plasma	LDA
Unkown	level4f123	245.2262	2.0	1.14E+06	1.37E+06	1.32E+06	1.94E+06	8.55E+05	1.32E+06	3.98E+05	30.05	plasma	CD
Unkown	level4f124	247.1447	7.5	8.17E+06	1.45E+07	1.11E+07	9.73E+06	6.66E+06	1.00E+07	3.01E+06	30.04	plasma	LDA
Unkown	level4f124	247.1447	7.6	2.08E+06	3.66E+06	2.77E+06	2.45E+06	1.82E+06	2.56E+06	7.13E+05	27.87	plasma	CD
Unkown	level4f124	247.1447	7.5	1.95E+06	7.02E+04	2.81E+06	2.42E+06	1.69E+06	1.79E+06	1.05E+06	58.85	plasma	XCMS
Unkown	level4f125	248.1097	17.8	3.09E+06	2.77E+06	3.81E+06	3.31E+06	1.97E+06	2.99E+06	6.86E+05	22.96	plasma	LDA
Unkown	level4f125	248.1097	17.7	7.43E+05	6.72E+05	1.00E+06	9.08E+05	5.28E+05	7.71E+05	1.89E+05	24.48	plasma	CD
Unkown	level4f125	248.1095	17.7	6.86E+05	5.80E+03	8.06E+05	6.57E+05	2.66E+05	4.84E+05	3.36E+05	69.33	plasma	XCMS
Unkown	level4f126	249.0618	12.0	1.45E+07	1.40E+07	1.37E+07	1.30E+07	1.43E+07	1.39E+07	6.12E+05	4.40	plasma	LDA
Unkown	level4f126	249.0615	12.0	3.69E+06	3.65E+06	3.59E+06	3.30E+06	3.46E+06	3.54E+06	1.61E+05	4.55	plasma	CD
Unkown	level4f126	249.0616	12.0	3.59E+06	3.46E+06	3.33E+06	3.07E+06	3.38E+06	3.37E+06	1.92E+05	5.69	plasma	XCMS

Unkown	level4f127	249.1357	18.1	1.69E+06	2.50E+06	1.44E+06	9.15E+05	2.66E+05	1.36E+06	8.37E+05	61.50	plasma	LDA
Unkown	level4f127	249.1356	18.1	2.10E+06	2.83E+06	1.94E+06	1.38E+06	5.85E+05	1.77E+06	8.38E+05	47.44	plasma	CD
Unkown	level4f128	250.1362	18.1	7.20E+06	9.60E+06	6.50E+06	4.63E+06	2.08E+06	6.00E+06	2.82E+06	46.99	plasma	LDA
Unkown	level4f128	250.1362	18.1	8.17E+05	2.23E+06	1.58E+06	1.15E+06	5.22E+05	1.26E+06	6.71E+05	53.19	plasma	CD
Unkown	level4f128	250.1368	18.1	1.69E+06	3.06E+04	9.86E+03	8.01E+03	5.12E+05	4.50E+05	7.26E+05	161.23	plasma	XCMS
Unkown	level4f129	253.1289	2.6	1.06E+07	3.22E+06	2.43E+06	2.36E+06	2.18E+06	4.15E+06	3.61E+06	86.92	plasma	LDA
Unkown	level4f129	253.1288	3.0	2.88E+06	2.78E+06	2.34E+06	2.42E+06	2.76E+06	2.64E+06	2.39E+05	9.06	plasma	CD
Unkown	level4f129	253.1289	3.0	1.86E+06	1.70E+06	1.69E+06	1.83E+06	1.65E+06	1.75E+06	9.33E+04	5.34	plasma	XCMS
Unkown	level4f130	254.8242	11.5	1.29E+07	1.84E+07	1.70E+07	1.63E+07	1.72E+07	1.64E+07	2.07E+06	12.63	plasma	LDA
Unkown	level4f130	254.8244	11.5	6.29E+06	1.14E+07	1.02E+07	5.83E+06	9.26E+06	8.60E+06	2.44E+06	28.44	plasma	CD
Unkown	level4f130	254.8242	11.5	3.18E+06	2.03E+06	3.68E+06	3.39E+06	4.18E+06	3.29E+06	8.00E+05	24.28	plasma	XCMS
Unkown	level4f131	255.9821	10.2	2.72E+06	3.39E+05	6.09E+05	6.59E+05	2.98E+05	9.24E+05	1.01E+06	109.75	plasma	LDA
Unkown	level4f131	255.9821	10.2	7.03E+05	1.02E+05	1.64E+05	1.86E+05	5.52E+04	2.42E+05	2.63E+05	108.63	plasma	CD
Unkown	level4f131	255.9826	10.1	5.74E+05	8.68E+04	1.00E+05	1.66E+05	5.11E+04	1.96E+05	2.16E+05	110.22	plasma	XCMS
Unkown	level4f132	256.0161	6.9	2.48E+08	2.14E+08	2.36E+08	2.86E+08	2.80E+08	2.53E+08	3.04E+07	12.01	plasma	LDA
Unkown	level4f132	256.0140	6.9	4.11E+07	3.95E+07	4.02E+07	4.97E+07	4.93E+07	4.39E+07	5.08E+06	11.55	plasma	CD
Unkown	level4f132	256.0158	6.8	5.82E+07	4.96E+07	5.54E+07	6.70E+07	6.77E+07	5.96E+07	7.75E+06	13.00	plasma	XCMS
Unkown	level4f133	256.1758	2.8	1.24E+07	6.54E+06	1.89E+07	2.05E+07	2.27E+07	1.62E+07	6.63E+06	40.93	plasma	LDA
Unkown	level4f133	256.1758	2.9	2.22E+06	3.32E+06	1.22E+06	3.15E+06	4.08E+06	2.80E+06	1.10E+06	39.31	plasma	CD
Unkown	level4f133	256.1761	2.9	4.13E+06	3.45E+06	3.48E+06	3.54E+06	5.11E+06	3.94E+06	7.11E+05	18.03	plasma	XCMS
Unkown	level4f134	256.8205	11.5	1.78E+07	2.59E+07	2.43E+07	2.33E+07	2.42E+07	2.31E+07	3.08E+06	13.34	plasma	LDA
Unkown	level4f134	256.8205	11.5	4.31E+06	6.83E+06	6.11E+06	5.17E+06	6.32E+06	5.75E+06	1.00E+06	17.46	plasma	CD
Unkown	level4f134	256.8212	11.5	4.37E+06	6.31E+06	5.24E+06	4.86E+06	5.85E+06	5.32E+06	7.70E+05	14.47	plasma	XCMS
Unkown	level4f135	257.1480	12.2	3.99E+07	3.94E+07	4.02E+07	3.42E+07	3.71E+07	3.82E+07	2.50E+06	6.56	plasma	LDA
Unkown	level4f135	257.1475	12.2	1.00E+07	9.61E+06	1.06E+07	8.56E+06	9.29E+06	9.62E+06	7.81E+05	8.12	plasma	CD
Unkown	level4f135	257.1477	12.2	9.64E+06	9.63E+06	9.86E+06	8.07E+06	8.97E+06	9.24E+06	7.30E+05	7.90	plasma	XCMS
Unkown	level4f136	258.8183	11.5	7.13E+06	1.06E+07	9.76E+06	9.39E+06	9.64E+06	9.30E+06	1.29E+06	13.89	plasma	LDA
Unkown	level4f136	258.8184	11.4	1.46E+06	2.78E+06	2.64E+06	2.67E+06	2.25E+06	2.36E+06	5.45E+05	23.06	plasma	CD
Unkown	level4f136	258.8182	11.5	1.77E+06	1.12E+06	2.15E+06	1.97E+06	2.40E+06	1.88E+06	4.82E+05	25.65	plasma	XCMS
Unkown	level4f137	260.7620	9.5	6.46E+06	9.36E+06	8.66E+06	8.94E+06	8.82E+06	8.45E+06	1.14E+06	13.53	plasma	LDA

Unkown	level4f137	260.7621	9.5	4.25E+06	4.36E+06	6.17E+06	5.27E+06	3.45E+06	4.70E+06	1.04E+06	22.20	plasma	CD
Unkown	level4f138	264.7556	9.5	3.05E+06	4.23E+06	4.51E+06	4.43E+06	4.51E+06	4.15E+06	6.22E+05	15.00	plasma	LDA
Unkown	level4f138	264.7556	9.5	7.48E+05	1.10E+06	1.20E+06	1.16E+06	1.10E+06	1.06E+06	1.81E+05	17.04	plasma	CD
Unkown	level4f139	271.0409	14.5	2.31E+07	2.21E+07	2.31E+07	2.41E+07	2.09E+07	2.27E+07	1.21E+06	5.35	plasma	LDA
Unkown	level4f139	271.0409	14.5	4.46E+06	8.49E+06	7.11E+06	5.56E+06	4.39E+06	6.00E+06	1.78E+06	29.59	plasma	CD
Unkown	level4f139	271.0407	14.5	3.49E+06	2.95E+06	2.60E+06	2.42E+06	2.22E+06	2.74E+06	5.00E+05	18.29	plasma	XCMS
Unkown	level4f140	271.0408	13.9	1.72E+07	1.80E+07	1.48E+07	1.39E+07	1.46E+07	1.57E+07	1.79E+06	11.40	plasma	LDA
Unkown	level4f140	271.0410	13.8	4.56E+06	3.16E+06	2.57E+06	3.64E+06	3.56E+06	3.50E+06	7.28E+05	20.82	plasma	CD
Unkown	level4f140	271.0408	13.9	4.84E+06	2.70E+06	3.57E+06	3.37E+06	3.11E+06	3.52E+06	8.07E+05	22.93	plasma	XCMS
Unkown	level4f141	277.2163	2.0	5.84E+06	5.59E+06	4.84E+06	5.02E+06	4.76E+06	5.21E+06	4.78E+05	9.19	plasma	LDA
Unkown	level4f141	277.2164	2.1	2.57E+06	2.66E+06	2.04E+06	2.66E+06	2.81E+06	2.55E+06	2.96E+05	11.62	plasma	CD
Unkown	level4f141	277.2169	2.0	9.56E+05	7.16E+05	8.10E+05	3.98E+05	4.51E+05	6.66E+05	2.37E+05	35.62	plasma	XCMS
Unkown	level4f142	278.2491	2.0	2.44E+07	2.42E+07	2.35E+07	2.81E+07	3.36E+07	2.68E+07	4.26E+06	15.93	plasma	LDA
Unkown	level4f142	278.2489	2.0	7.41E+06	6.31E+06	7.10E+06	7.42E+06	7.63E+06	7.17E+06	5.19E+05	7.23	plasma	CD
Unkown	level4f143	280.9868	13.6	5.65E+06	5.59E+06	5.97E+06	5.91E+06	5.41E+06	5.71E+06	2.34E+05	4.11	plasma	LDA
Unkown	level4f143	280.9869	13.5	3.60E+06	3.39E+06	3.53E+06	3.83E+06	2.94E+06	3.46E+06	3.29E+05	9.52	plasma	CD
Unkown	level4f143	280.9868	13.6	1.49E+06	1.40E+06	1.51E+06	1.52E+06	1.42E+06	1.47E+06	5.46E+04	3.72	plasma	XCMS
Unkown	level4f144	283.1633	12.2	1.01E+07	1.06E+07	1.08E+07	7.89E+06	9.30E+06	9.75E+06	1.20E+06	12.26	plasma	LDA
Unkown	level4f144	283.1633	12.1	2.62E+06	3.05E+06	2.78E+06	2.13E+06	1.96E+06	2.51E+06	4.54E+05	18.12	plasma	CD
Unkown	level4f144	283.1632	12.2	2.48E+06	2.61E+06	2.65E+06	1.95E+06	2.23E+06	2.38E+06	2.94E+05	12.33	plasma	XCMS
Unkown	level4f145	283.1756	2.6	1.10E+08	1.26E+08	1.09E+08	1.50E+08	1.19E+08	1.23E+08	1.66E+07	13.53	plasma	LDA
Unkown	level4f145	283.1760	2.6	5.99E+07	5.67E+07	3.95E+07	5.71E+07	4.98E+07	5.26E+07	8.18E+06	15.56	plasma	CD
Unkown	level4f145	283.1761	2.6	2.87E+07	3.50E+07	2.66E+07	2.82E+07	2.33E+07	2.84E+07	4.25E+06	14.97	plasma	XCMS
Unkown	level4f146	283.1761	2.7	7.81E+07	1.21E+08	1.30E+08	9.75E+07	1.08E+08	1.07E+08	2.02E+07	18.91	plasma	LDA
Unkown	level4f146	283.1760	2.6	5.99E+07	5.67E+07	3.95E+07	5.71E+07	4.98E+07	5.26E+07	8.18E+06	15.56	plasma	CD
Unkown	level4f146	283.1761	2.6	2.87E+07	3.50E+07	2.66E+07	2.82E+07	2.33E+07	2.84E+07	4.25E+06	14.97	plasma	XCMS
Unkown	level4f147	285.0476	10.7	8.98E+06	6.60E+06	8.68E+06	9.04E+06	8.61E+06	8.38E+06	1.02E+06	12.12	plasma	LDA
Unkown	level4f147	285.0476	10.6	2.98E+06	2.49E+06	2.53E+06	2.96E+06	3.07E+06	2.80E+06	2.72E+05	9.69	plasma	CD
Unkown	level4f147	285.0475	10.7	2.06E+06	1.63E+06	1.67E+06	1.81E+06	1.12E+06	1.66E+06	3.44E+05	20.73	plasma	XCMS
Unkown	level4f148	290.1598	7.8	9.52E+06	3.46E+06	4.93E+06	6.27E+06	4.00E+06	5.64E+06	2.42E+06	42.89	plasma	LDA

Unkown	level4f148	290.1598	7.8	2.36E+06	8.34E+05	1.15E+06	1.54E+06	1.03E+06	1.38E+06	6.03E+05	43.60	plasma	CD
Unkown	level4f149	294.2435	2.0	8.49E+06	1.50E+07	1.48E+07	1.75E+07	1.68E+07	1.45E+07	3.57E+06	24.56	plasma	LDA
Unkown	level4f149	294.2435	2.1	2.09E+06	2.26E+06	1.88E+06	2.53E+06	2.61E+06	2.27E+06	3.05E+05	13.40	plasma	CD
Unkown	level4f150	294.9384	13.6	1.67E+08	1.62E+08	1.76E+08	1.86E+08	1.79E+08	1.74E+08	9.55E+06	5.49	plasma	LDA
Unkown	level4f150	294.9384	13.7	5.62E+06	8.01E+06	7.63E+06	8.68E+06	6.55E+06	7.30E+06	1.21E+06	16.65	plasma	CD
Unkown	level4f150	294.9385	13.6	2.27E+05	5.65E+04	1.64E+07	7.65E+06	1.97E+07	8.81E+06	9.05E+06	102.78	plasma	XCMS
Unkown	level4f151	295.2259	1.9	1.08E+07	9.53E+06	5.75E+06	7.30E+06	7.17E+06	8.11E+06	2.03E+06	25.00	plasma	LDA
Unkown	level4f151	295.2259	1.9	1.41E+06	1.07E+06	1.03E+06	2.56E+05	6.92E+05	8.91E+05	4.36E+05	48.92	plasma	CD
Unkown	level4f151	295.2269	1.9	1.21E+06	1.70E+06	9.02E+05	1.01E+06	7.10E+05	1.11E+06	3.76E+05	33.97	plasma	XCMS
Unkown	level4f152	296.0096	14.1	2.81E+07	2.68E+07	2.96E+07	2.74E+07	2.40E+07	2.72E+07	2.05E+06	7.55	plasma	LDA
Unkown	level4f152	296.0099	14.0	7.10E+06	5.65E+06	7.36E+06	6.70E+06	5.76E+06	6.51E+06	7.76E+05	11.92	plasma	CD
Unkown	level4f152	296.0101	14.0	6.64E+06	4.88E+06	7.14E+06	6.62E+06	2.65E+04	5.06E+06	2.94E+06	58.14	plasma	XCMS
Unkown	level4f153	296.2595	1.9	1.32E+08	8.15E+07	8.78E+07	9.53E+07	1.71E+08	1.13E+08	3.74E+07	33.01	plasma	LDA
Unkown	level4f153	296.2592	1.9	1.98E+07	1.05E+07	1.17E+07	2.93E+07	3.00E+07	2.03E+07	9.29E+06	45.84	plasma	CD
Unkown	level4f153	296.2592	1.9	2.01E+07	2.12E+07	2.38E+07	2.57E+07	3.00E+07	2.41E+07	3.90E+06	16.17	plasma	XCMS
Unkown	level4f154	297.1550	2.5	9.19E+06	1.43E+07	1.12E+07	1.05E+07	6.69E+06	1.04E+07	2.80E+06	26.90	plasma	LDA
Unkown	level4f154	297.1551	3.0	6.92E+06	8.36E+06	7.76E+06	7.92E+06	7.35E+06	7.66E+06	5.48E+05	7.16	plasma	CD
Unkown	level4f154	297.1552	2.9	3.25E+06	3.48E+06	3.20E+06	3.41E+06	2.73E+06	3.21E+06	2.92E+05	9.10	plasma	XCMS
Unkown	level4f155	298.0248	13.6	1.55E+07	1.55E+07	1.52E+07	1.32E+07	1.26E+07	1.44E+07	1.39E+06	9.69	plasma	LDA
Unkown	level4f155	298.0249	13.5	4.34E+06	3.99E+06	3.99E+06	3.60E+06	3.28E+06	3.84E+06	4.09E+05	10.66	plasma	CD
Unkown	level4f155	298.0249	13.5	3.93E+06	3.61E+06	2.28E+06	3.28E+06	2.89E+06	3.20E+06	6.43E+05	20.11	plasma	XCMS
Unkown	level4f156	298.0534	32.9	6.59E+06	1.44E+07	1.16E+07	1.13E+07	1.05E+07	1.09E+07	2.82E+06	25.94	plasma	LDA
Unkown	level4f156	298.0531	32.9	1.20E+06	3.93E+06	2.72E+06	1.87E+06	2.48E+06	2.44E+06	1.02E+06	41.83	plasma	CD
Unkown	level4f156	298.0529	32.9	1.00E+06	2.96E+06	2.34E+06	2.45E+06	2.38E+06	2.23E+06	7.29E+05	32.74	plasma	XCMS
Unkown	level4f157	299.1395	1.8	1.99E+08	2.21E+08	1.94E+08	1.54E+08	1.14E+08	1.76E+08	4.21E+07	23.87	plasma	LDA
Unkown	level4f157	299.1395	1.8	4.40E+07	5.77E+07	4.95E+07	3.61E+07	2.78E+07	4.30E+07	1.16E+07	26.98	plasma	CD
Unkown	level4f157	299.1393	1.8	4.99E+07	5.42E+07	6.73E+05	3.96E+07	2.88E+07	3.46E+07	2.14E+07	61.74	plasma	XCMS
Unkown	level4f158	302.1971	7.7	2.83E+06	4.90E+05	4.60E+05	6.43E+05	5.16E+05	9.87E+05	1.03E+06	104.45	plasma	LDA
Unkown	level4f158	302.1971	7.7	7.73E+05	1.01E+05	1.60E+05	1.42E+05	1.16E+05	2.59E+05	2.89E+05	111.59	plasma	CD
Unkown	level4f159	304.3006	3.4	2.35E+07	2.65E+07	3.94E+07	5.98E+07	7.18E+07	4.42E+07	2.10E+07	47.61	plasma	LDA

Unkown	level4f159	304.3005	3.3	7.74E+05	1.74E+06	7.65E+06	1.40E+07	1.98E+07	8.79E+06	8.11E+06	92.30	plasma	CD
Unkown	level4f159	304.3004	3.4	5.58E+06	5.86E+06	8.00E+06	1.27E+07	1.56E+07	9.53E+06	4.40E+06	46.18	plasma	XCMS
Unkown	level4f160	304.8956	14.3	2.59E+06	1.26E+06	1.20E+06	1.20E+06	4.36E+06	2.12E+06	1.38E+06	65.16	plasma	LDA
Unkown	level4f160	304.8956	14.3	1.29E+05	1.05E+05	2.76E+05	7.50E+04	9.08E+05	2.99E+05	3.49E+05	116.94	plasma	CD
Unkown	level4f160	304.8947	14.3	7.51E+05	3.04E+05	3.21E+05	2.53E+05	1.16E+06	5.59E+05	3.93E+05	70.35	plasma	XCMS
Unkown	level4f161	305.9612	14.4	1.53E+07	1.88E+07	1.69E+07	1.69E+07	1.39E+07	1.64E+07	1.84E+06	11.26	plasma	LDA
Unkown	level4f161	305.9615	14.4	2.89E+06	4.43E+06	4.17E+06	4.00E+06	3.37E+06	3.77E+06	6.28E+05	16.65	plasma	CD
Unkown	level4f161	305.9615	14.4	3.80E+05	3.92E+06	4.10E+06	4.05E+06	3.40E+06	3.17E+06	1.58E+06	49.99	plasma	XCMS
Unkown	level4f162	312.2540	2.0	3.60E+06	3.01E+06	3.02E+06	3.90E+06	3.04E+06	3.31E+06	4.13E+05	12.47	plasma	LDA
Unkown	level4f162	312.2538	2.1	2.42E+06	2.74E+06	2.26E+06	3.21E+06	3.01E+06	2.73E+06	3.95E+05	14.46	plasma	CD
Unkown	level4f163	312.7821	11.5	8.17E+06	1.39E+07	1.32E+07	1.27E+07	1.19E+07	1.20E+07	2.24E+06	18.75	plasma	LDA
Unkown	level4f163	312.7829	11.5	7.10E+06	6.88E+06	1.09E+07	1.14E+07	2.73E+06	7.80E+06	3.52E+06	45.11	plasma	CD
Unkown	level4f163	312.7828	11.5	2.05E+06	3.43E+06	2.80E+06	2.73E+06	2.90E+06	2.78E+06	4.92E+05	17.68	plasma	XCMS
Unkown	level4f164	314.2335	4.6	6.24E+06	1.77E+06	5.60E+06	7.29E+06	1.07E+07	6.33E+06	3.23E+06	51.02	plasma	LDA
Unkown	level4f164	314.2332	4.6	1.02E+06	1.96E+05	4.11E+05	1.44E+06	2.40E+06	1.09E+06	8.83E+05	80.70	plasma	CD
Unkown	level4f164	314.2329	4.7	1.11E+06	5.93E+05	7.48E+05	1.27E+06	2.38E+06	1.22E+06	7.04E+05	57.69	plasma	XCMS
Unkown	level4f165	314.7801	11.5	1.64E+07	2.62E+07	2.45E+07	2.39E+07	2.32E+07	2.28E+07	3.77E+06	16.52	plasma	LDA
Unkown	level4f165	314.7801	11.5	6.55E+06	1.11E+07	1.04E+07	1.05E+07	8.13E+06	9.34E+06	1.93E+06	20.65	plasma	CD
Unkown	level4f165	314.7800	11.5	4.02E+06	6.39E+06	5.11E+06	5.18E+06	5.59E+06	5.26E+06	8.58E+05	16.32	plasma	XCMS
Unkown	level4f166	316.9829	14.4	2.54E+06	2.71E+06	2.74E+06	2.91E+06	2.22E+06	2.62E+06	2.64E+05	10.07	plasma	LDA
Unkown	level4f166	316.9829	14.4	6.52E+05	6.78E+05	6.78E+05	6.97E+05	5.48E+05	6.51E+05	5.95E+04	9.15	plasma	CD
Unkown	level4f167	320.2568	2.0	1.88E+06	2.10E+06	1.84E+06	3.25E+06	2.23E+06	2.26E+06	5.77E+05	25.51	plasma	LDA
Unkown	level4f167	320.2568	2.0	9.71E+04	4.24E+05	3.95E+05	5.16E+05	2.64E+05	3.39E+05	1.63E+05	47.93	plasma	CD
Unkown	level4f168	322.8121	11.5	4.43E+06	6.77E+06	7.39E+06	6.91E+06	7.08E+06	6.52E+06	1.19E+06	18.22	plasma	LDA
Unkown	level4f168	322.8115	11.5	3.37E+06	4.25E+06	4.69E+06	4.78E+06	4.36E+06	4.29E+06	5.59E+05	13.04	plasma	CD
Unkown	level4f168	322.8116	11.5	1.07E+06	8.58E+05	1.75E+06	1.67E+06	1.69E+06	1.41E+06	4.13E+05	29.29	plasma	XCMS
Unkown	level4f169	326.0857	4.5	7.20E+06	2.96E+06	8.70E+06	1.68E+07	1.88E+07	1.09E+07	6.68E+06	61.35	plasma	LDA
Unkown	level4f169	326.0860	4.4	2.24E+06	7.47E+05	2.06E+06	4.43E+06	6.09E+06	3.11E+06	2.13E+06	68.30	plasma	CD
Unkown	level4f169	326.0859	4.4	1.74E+06	6.59E+05	2.23E+06	4.21E+06	4.00E+06	2.57E+06	1.52E+06	59.01	plasma	XCMS
Unkown	level4f170	327.2021	2.9	5.39E+07	8.78E+07	7.21E+07	8.61E+07	8.06E+07	7.61E+07	1.38E+07	18.20	plasma	LDA

Unkown	level4f170	327.2023	3.0	4.65E+07	4.79E+07	4.54E+07	5.26E+07	4.94E+07	4.84E+07	2.82E+06	5.83	plasma	CD
Unkown	level4f170	327.2021	3.0	1.66E+07	2.53E+07	1.93E+07	2.28E+07	1.82E+05	1.68E+07	9.86E+06	58.68	plasma	XCMS
Unkown	level4f171	327.2021	2.6	7.64E+07	1.01E+08	8.28E+07	9.28E+07	9.28E+07	8.91E+07	9.56E+06	10.73	plasma	LDA
Unkown	level4f171	327.2023	2.6	8.22E+07	8.30E+07	6.85E+07	9.00E+07	6.38E+07	7.75E+07	1.09E+07	14.08	plasma	CD
Unkown	level4f171	327.2024	2.6	2.01E+07	2.26E+07	2.06E+07	2.20E+07	2.36E+07	2.18E+07	1.45E+06	6.64	plasma	XCMS
Unkown	level4f172	328.2335	2.9	3.39E+06	9.71E+06	8.88E+06	6.87E+06	4.75E+06	6.72E+06	2.67E+06	39.80	plasma	LDA
Unkown	level4f172	328.2335	3.0	1.14E+05	1.04E+06	9.91E+05	7.44E+05	6.57E+05	7.10E+05	3.71E+05	52.22	plasma	CD
Unkown	level4f172	328.2334	2.9	1.54E+06	2.78E+06	2.39E+06	1.81E+06	1.07E+06	1.92E+06	6.78E+05	35.34	plasma	XCMS
Unkown	level4f173	332.3319	3.1	2.73E+06	7.96E+05	3.24E+05	1.24E+06	1.40E+07	3.82E+06	5.76E+06	150.89	plasma	LDA
Unkown	level4f173	332.3318	3.2	5.19E+05	5.95E+05	1.05E+06	2.26E+06	3.35E+06	1.56E+06	1.22E+06	78.63	plasma	CD
Unkown	level4f173	332.3317	3.3	5.74E+05	7.39E+05	9.38E+05	2.13E+06	3.03E+06	1.48E+06	1.06E+06	71.47	plasma	XCMS
Unkown	level4f174	334.6952	9.5	5.63E+06	8.37E+06	8.28E+06	8.01E+06	8.44E+06	7.75E+06	1.19E+06	15.38	plasma	LDA
Unkown	level4f174	334.6949	9.5	3.89E+06	4.66E+06	2.15E+06	2.07E+06	5.98E+06	3.75E+06	1.67E+06	44.60	plasma	CD
Unkown	level4f175	336.6911	9.5	1.02E+07	1.51E+07	1.43E+07	1.44E+07	1.50E+07	1.38E+07	2.05E+06	14.84	plasma	LDA
Unkown	level4f175	336.6918	9.5	4.39E+06	6.47E+06	6.56E+06	6.00E+06	6.66E+06	6.01E+06	9.44E+05	15.69	plasma	CD
Unkown	level4f176	338.0510	10.8	2.25E+07	1.55E+07	2.38E+07	3.07E+07	2.11E+07	2.27E+07	5.45E+06	24.04	plasma	LDA
Unkown	level4f176	338.0510	10.6	9.62E+06	7.53E+06	1.13E+07	7.14E+06	9.41E+06	9.00E+06	1.70E+06	18.89	plasma	CD
Unkown	level4f176	338.0511	10.6	4.78E+06	4.64E+06	1.44E+04	4.97E+06	5.14E+06	3.91E+06	2.18E+06	55.90	plasma	XCMS
Unkown	level4f177	338.6897	9.5	6.47E+06	9.54E+06	9.77E+06	9.47E+06	9.59E+06	8.97E+06	1.40E+06	15.63	plasma	LDA
Unkown	level4f177	338.6897	9.5	1.85E+06	1.10E+06	2.85E+06	1.75E+06	3.01E+06	2.11E+06	8.02E+05	37.97	plasma	CD
Unkown	level4f178	340.0479	10.7	1.55E+07	1.72E+07	1.56E+07	1.71E+07	1.47E+07	1.60E+07	1.08E+06	6.72	plasma	LDA
Unkown	level4f178	340.0478	10.6	4.59E+06	4.72E+06	3.56E+06	4.20E+06	4.32E+06	4.28E+06	4.53E+05	10.58	plasma	CD
Unkown	level4f178	340.0480	10.6	2.49E+06	2.52E+06	2.50E+06	1.55E+04	3.32E+06	2.17E+06	1.25E+06	57.82	plasma	XCMS
Unkown	level4f179	341.1814	2.6	3.17E+06	8.71E+06	7.35E+06	5.68E+06	3.74E+06	5.73E+06	2.35E+06	40.94	plasma	LDA
Unkown	level4f179	341.1818	3.0	9.55E+06	1.13E+07	8.58E+06	1.03E+07	8.87E+06	9.72E+06	1.11E+06	11.46	plasma	CD
Unkown	level4f180	345.1302	9.2	1.17E+07	1.47E+07	1.46E+07	1.33E+07	1.11E+07	1.31E+07	1.61E+06	12.31	plasma	LDA
Unkown	level4f180	345.1300	9.1	2.86E+06	3.79E+06	3.79E+06	2.48E+06	2.96E+06	3.17E+06	5.87E+05	18.49	plasma	CD
Unkown	level4f180	345.1300	9.1	2.86E+06	3.60E+06	3.61E+06	3.34E+06	2.84E+06	3.25E+06	3.81E+05	11.73	plasma	XCMS
Unkown	level4f181	345.2318	2.9	5.37E+06	1.15E+07	8.47E+06	1.31E+07	1.35E+07	1.04E+07	3.42E+06	32.99	plasma	LDA
Unkown	level4f181	345.2318	3.0	1.35E+06	1.16E+06	9.33E+05	1.98E+06	1.21E+06	1.33E+06	3.96E+05	29.83	plasma	CD

Unkown	level4f181	345.2318	3.0	2.77E+06	2.34E+06	2.03E+06	3.49E+06	3.29E+06	2.79E+06	6.17E+05	22.15	plasma	XCMS
Unkown	level4f182	346.0259	11.8	3.55E+06	4.54E+05	5.59E+05	6.19E+05	7.64E+05	1.19E+06	1.32E+06	111.38	plasma	LDA
Unkown	level4f182	346.0259	11.7	9.72E+05	1.01E+05	1.86E+05	7.12E+04	2.06E+05	3.07E+05	3.76E+05	122.36	plasma	CD
Unkown	level4f182	346.0252	11.8	8.63E+05	8.86E+04	1.15E+05	1.18E+05	1.64E+05	2.70E+05	3.33E+05	123.40	plasma	XCMS
Unkown	level4f183	347.0663	20.4	1.96E+07	3.56E+06	2.43E+06	1.75E+07	1.18E+07	1.10E+07	7.85E+06	71.42	plasma	LDA
Unkown	level4f183	347.0657	20.3	5.24E+06	1.09E+06	5.42E+05	4.62E+06	3.10E+06	2.92E+06	2.08E+06	71.23	plasma	CD
Unkown	level4f183	347.0654	20.4	4.12E+06	8.46E+05	8.30E+05	4.22E+06	1.53E+06	2.31E+06	1.72E+06	74.59	plasma	XCMS
Unkown	level4f184	352.8986	11.8	1.00E+07	1.04E+07	1.17E+07	1.22E+07	1.15E+07	1.12E+07	9.14E+05	8.19	plasma	LDA
Unkown	level4f184	352.8983	11.7	3.45E+06	3.36E+06	3.95E+06	3.27E+06	3.31E+06	3.47E+06	2.78E+05	8.01	plasma	CD
Unkown	level4f184	352.8982	11.7	2.07E+06	2.71E+06	2.75E+06	2.57E+06	2.45E+06	2.51E+06	2.74E+05	10.91	plasma	XCMS
Unkown	level4f185	355.0713	1.8	8.73E+05	1.61E+06	2.03E+06	3.61E+06	4.97E+06	2.62E+06	1.65E+06	63.17	plasma	LDA
Unkown	level4f185	355.0711	1.8	2.13E+05	2.93E+05	5.20E+05	9.21E+05	1.46E+06	6.82E+05	5.15E+05	75.52	plasma	CD
Unkown	level4f186	356.2282	2.2	2.99E+06	3.57E+06	3.65E+06	3.09E+06	2.02E+06	3.06E+06	6.54E+05	21.33	plasma	LDA
Unkown	level4f186	356.2282	2.3	5.26E+05	9.01E+05	8.21E+05	6.08E+05	4.50E+05	6.61E+05	1.93E+05	29.20	plasma	CD
Unkown	level4f187	362.0215	14.6	1.36E+07	1.76E+07	1.52E+07	1.21E+07	9.50E+06	1.36E+07	3.08E+06	22.59	plasma	LDA
Unkown	level4f187	362.0212	14.6	3.33E+06	4.22E+06	3.41E+06	2.98E+06	2.41E+06	3.27E+06	6.63E+05	20.27	plasma	CD
Unkown	level4f187	362.0208	14.6	3.25E+06	4.33E+06	3.39E+06	7.58E+03	1.95E+06	2.59E+06	1.67E+06	64.61	plasma	XCMS
Unkown	level4f188	362.9279	13.2	1.87E+08	2.13E+08	3.21E+08	1.37E+08	2.11E+08	2.14E+08	6.72E+07	31.45	plasma	LDA
Unkown	level4f188	362.9279	13.0	1.72E+07	1.77E+07	1.97E+07	1.87E+07	7.34E+06	1.61E+07	5.00E+06	31.01	plasma	CD
Unkown	level4f188	362.9272	13.1	2.22E+07	3.48E+07	1.62E+07	1.41E+07	2.45E+07	2.23E+07	8.14E+06	36.44	plasma	XCMS
Unkown	level4f189	368.2806	4.3	1.05E+07	7.08E+06	6.80E+06	6.19E+06	6.02E+06	7.31E+06	1.82E+06	24.84	plasma	LDA
Unkown	level4f189	368.2799	4.3	2.90E+06	1.95E+06	1.81E+06	1.79E+06	1.60E+06	2.01E+06	5.13E+05	25.55	plasma	CD
Unkown	level4f189	368.2804	4.3	2.64E+06	1.16E+06	1.61E+06	1.47E+06	1.40E+06	1.66E+06	5.76E+05	34.77	plasma	XCMS
Unkown	level4f190	368.7207	2.8	2.06E+07	8.49E+06	1.30E+07	7.75E+06	4.18E+06	1.08E+07	6.33E+06	58.51	plasma	LDA
Unkown	level4f190	368.7205	2.7	2.71E+06	3.81E+06	2.08E+06	2.55E+06	1.26E+06	2.48E+06	9.33E+05	37.61	plasma	CD
Unkown	level4f190	368.7206	2.9	3.08E+06	3.38E+06	2.09E+06	2.92E+06	3.44E+06	2.98E+06	5.43E+05	18.20	plasma	XCMS
Unkown	level4f191	370.2962	4.1	3.20E+07	1.24E+06	1.85E+07	1.12E+06	2.16E+07	1.49E+07	1.35E+07	90.52	plasma	LDA
Unkown	level4f191	370.2960	4.2	8.53E+06	4.16E+06	4.55E+06	5.72E+06	5.99E+06	5.79E+06	1.71E+06	29.58	plasma	CD
Unkown	level4f191	370.2958	4.2	7.40E+06	1.65E+06	3.90E+06	5.27E+06	5.34E+06	4.71E+06	2.12E+06	44.96	plasma	XCMS
Unkown	level4f192	370.7416	11.5	5.49E+06	1.03E+07	1.03E+07	9.73E+06	9.55E+06	9.08E+06	2.04E+06	22.47	plasma	LDA

Unkown	level4f192	370.7419	11.5	6.78E+06	1.16E+07	1.15E+07	1.44E+07	1.22E+07	1.13E+07	2.77E+06	24.56	plasma	CD
Unkown	level4f192	370.7417	11.5	1.38E+06	2.53E+06	2.20E+06	2.09E+06	2.42E+06	2.12E+06	4.51E+05	21.26	plasma	XCMS
Unkown	level4f193	370.9565	13.6	7.08E+06	7.36E+06	7.96E+06	6.59E+06	6.19E+06	7.04E+06	6.84E+05	9.71	plasma	LDA
Unkown	level4f193	370.9563	13.5	1.85E+06	1.99E+06	1.81E+06	2.12E+06	1.74E+06	1.90E+06	1.52E+05	7.99	plasma	CD
Unkown	level4f193	370.9563	13.6	1.82E+06	1.86E+06	1.92E+06	1.63E+06	1.55E+06	1.76E+06	1.60E+05	9.12	plasma	XCMS
Unkown	level4f194	371.1027	1.8	1.07E+06	2.19E+06	3.34E+06	5.38E+06	6.74E+06	3.74E+06	2.31E+06	61.82	plasma	LDA
Unkown	level4f194	371.1027	1.8	1.05E+05	3.18E+05	9.03E+05	1.35E+06	1.85E+06	9.05E+05	7.19E+05	79.50	plasma	CD
Unkown	level4f195	372.7379	11.5	1.46E+07	2.53E+07	2.41E+07	2.35E+07	2.38E+07	2.23E+07	4.35E+06	19.51	plasma	LDA
Unkown	level4f195	372.7379	11.5	6.68E+06	1.26E+07	1.20E+07	9.68E+06	1.25E+07	1.07E+07	2.54E+06	23.74	plasma	CD
Unkown	level4f195	372.7389	11.5	3.59E+06	4.61E+06	5.12E+06	5.08E+06	5.81E+06	4.84E+06	8.21E+05	16.96	plasma	XCMS
Unkown	level4f196	373.9488	14.4	4.08E+06	5.46E+06	5.12E+06	5.16E+06	3.89E+06	4.74E+06	7.09E+05	14.94	plasma	LDA
Unkown	level4f196	373.9494	14.4	9.96E+05	1.41E+06	1.28E+06	1.20E+06	9.79E+05	1.17E+06	1.86E+05	15.83	plasma	CD
Unkown	level4f196	373.9491	14.4	4.31E+05	1.23E+06	1.28E+06	1.25E+06	9.92E+05	1.04E+06	3.57E+05	34.48	plasma	XCMS
Unkown	level4f197	380.7705	11.5	6.00E+06	1.15E+07	1.09E+07	1.02E+07	1.03E+07	9.78E+06	2.17E+06	22.22	plasma	LDA
Unkown	level4f197	380.7705	11.5	6.46E+06	9.90E+06	5.47E+06	6.01E+06	1.08E+07	7.73E+06	2.45E+06	31.70	plasma	CD
Unkown	level4f197	380.7705	11.5	1.47E+06	2.81E+06	2.61E+06	2.46E+06	2.45E+06	2.36E+06	5.19E+05	22.01	plasma	XCMS
Unkown	level4f198	382.7682	11.5	1.25E+07	2.15E+07	2.11E+07	2.02E+07	2.07E+07	1.92E+07	3.79E+06	19.72	plasma	LDA
Unkown	level4f198	382.7682	11.5	3.34E+06	5.96E+06	4.92E+06	5.60E+06	5.77E+06	5.12E+06	1.07E+06	20.87	plasma	CD
Unkown	level4f198	382.7677	11.5	2.97E+06	3.41E+06	5.07E+06	4.79E+06	5.00E+06	4.25E+06	9.86E+05	23.21	plasma	XCMS
Unkown	level4f199	383.1169	13.9	6.45E+06	7.19E+06	6.28E+06	6.23E+06	5.94E+06	6.42E+06	4.68E+05	7.29	plasma	LDA
Unkown	level4f199	383.1167	13.8	1.76E+06	1.88E+06	1.55E+06	1.05E+06	1.59E+06	1.57E+06	3.17E+05	20.25	plasma	CD
Unkown	level4f199	383.1172	13.9	1.65E+06	1.84E+06	1.61E+06	1.63E+06	1.45E+06	1.64E+06	1.38E+05	8.43	plasma	XCMS
Unkown	level4f200	383.1170	14.6	9.08E+06	8.93E+06	9.10E+06	8.12E+06	7.12E+06	8.47E+06	8.53E+05	10.07	plasma	LDA
Unkown	level4f200	383.1170	14.6	2.36E+06	1.62E+06	2.28E+06	2.00E+06	1.82E+06	2.02E+06	3.08E+05	15.26	plasma	CD
Unkown	level4f200	383.1171	14.6	2.26E+06	2.33E+06	2.30E+06	2.02E+06	1.40E+06	2.06E+06	3.89E+05	18.89	plasma	XCMS
Unkown	level4f201	385.0366	12.0	2.63E+06	2.68E+06	2.23E+06	2.59E+06	3.28E+06	2.68E+06	3.80E+05	14.17	plasma	LDA
Unkown	level4f201	385.0366	12.0	7.37E+05	6.57E+05	6.50E+05	6.79E+05	8.20E+05	7.08E+05	7.08E+04	9.99	plasma	CD
Unkown	level4f201	385.0362	12.0	6.33E+05	6.62E+05	5.96E+05	6.49E+05	7.84E+05	6.65E+05	7.08E+04	10.65	plasma	XCMS
Unkown	level4f202	388.2553	2.6	2.22E+08	2.48E+08	2.02E+08	2.23E+08	2.27E+08	2.24E+08	1.64E+07	7.32	plasma	LDA
Unkown	level4f202	388.2597	2.6	7.03E+05	6.16E+05	4.88E+05	5.08E+05	9.26E+05	6.48E+05	1.78E+05	27.42	plasma	CD

Unkown	level4f202	388.2557	2.6	5.80E+07	5.94E+07	4.96E+07	5.36E+07	5.09E+07	5.43E+07	4.30E+06	7.92	plasma	XCMS
Unkown	level4f203	388.2557	2.9	1.81E+08	1.93E+08	1.41E+08	1.82E+08	1.86E+08	1.77E+08	2.03E+07	11.50	plasma	LDA
Unkown	level4f203	388.2557	3.0	4.28E+07	4.24E+07	3.78E+07	4.30E+07	2.60E+07	3.84E+07	7.29E+06	18.98	plasma	CD
Unkown	level4f203	388.2554	3.0	6.39E+07	6.48E+07	5.29E+07	6.06E+07	3.69E+07	5.58E+07	1.16E+07	20.74	plasma	XCMS
Unkown	level4f204	390.7981	11.6	3.03E+06	4.73E+06	5.40E+06	5.19E+06	5.55E+06	4.78E+06	1.02E+06	21.43	plasma	LDA
Unkown	level4f204	390.7991	11.6	2.46E+06	2.31E+06	2.77E+06	2.80E+06	2.48E+06	2.57E+06	2.10E+05	8.18	plasma	CD
Unkown	level4f204	390.7991	11.5	8.02E+05	1.82E+04	1.00E+06	8.88E+05	9.58E+05	7.34E+05	4.07E+05	55.49	plasma	XCMS
Unkown	level4f205	392.7494	2.6	1.08E+07	8.24E+06	1.45E+07	1.69E+07	8.29E+06	1.18E+07	3.86E+06	32.85	plasma	LDA
Unkown	level4f205	392.7494	2.6	1.54E+06	1.88E+06	1.44E+06	1.34E+06	2.32E+06	1.71E+06	4.00E+05	23.44	plasma	CD
Unkown	level4f205	392.7492	2.8	2.88E+06	2.92E+06	2.60E+06	2.47E+06	2.61E+06	2.70E+06	1.93E+05	7.16	plasma	XCMS
Unkown	level4f206	398.3264	4.1	3.71E+06	3.13E+06	1.94E+06	2.95E+06	3.18E+06	2.98E+06	6.48E+05	21.74	plasma	LDA
Unkown	level4f206	398.3264	4.2	1.08E+06	5.82E+05	5.25E+05	6.83E+04	8.09E+04	4.67E+05	4.17E+05	89.46	plasma	CD
Unkown	level4f206	398.3264	4.0	9.81E+05	5.44E+05	5.24E+05	7.48E+05	8.40E+05	7.27E+05	1.95E+05	26.83	plasma	XCMS
Unkown	level4f207	400.2551	2.3	1.73E+06	2.30E+06	2.58E+06	3.33E+06	1.73E+06	2.33E+06	6.70E+05	28.72	plasma	LDA
Unkown	level4f207	400.2548	2.3	4.54E+05	6.67E+05	9.38E+05	9.71E+05	5.59E+05	7.18E+05	2.29E+05	31.95	plasma	CD
Unkown	level4f208	400.8281	11.6	2.21E+06	2.91E+05	3.11E+06	2.62E+06	3.00E+05	1.71E+06	1.33E+06	77.78	plasma	LDA
Unkown	level4f208	400.8279	11.6	1.13E+06	1.36E+06	1.38E+06	1.14E+06	1.24E+06	1.25E+06	1.16E+05	9.26	plasma	CD
Unkown	level4f208	400.8276	11.6	4.63E+05	6.53E+05	7.66E+05	6.08E+05	7.88E+05	6.56E+05	1.31E+05	20.03	plasma	XCMS
Unkown	level4f209	402.2340	2.5	3.89E+07	5.08E+07	6.79E+07	3.63E+07	3.58E+07	4.59E+07	1.37E+07	29.86	plasma	LDA
Unkown	level4f209	402.2345	3.0	7.83E+06	1.05E+07	7.81E+06	8.04E+06	6.47E+06	8.12E+06	1.45E+06	17.87	plasma	CD
Unkown	level4f209	402.2343	3.0	7.91E+06	9.56E+06	7.65E+06	8.23E+06	7.13E+06	8.10E+06	9.11E+05	11.26	plasma	XCMS
Unkown	level4f210	405.3730	1.9	5.07E+06	4.34E+06	4.27E+06	3.51E+06	2.59E+06	3.96E+06	9.41E+05	23.77	plasma	LDA
Unkown	level4f210	405.3730	1.9	1.38E+06	7.73E+05	8.13E+05	8.18E+05	3.58E+05	8.27E+05	3.62E+05	43.75	plasma	CD
Unkown	level4f211	407.2394	7.1	4.78E+07	6.14E+07	5.32E+07	6.20E+07	5.21E+07	5.53E+07	6.19E+06	11.19	plasma	LDA
Unkown	level4f211	407.2394	7.1	1.24E+07	1.65E+07	1.32E+07	1.71E+07	1.39E+07	1.46E+07	2.07E+06	14.12	plasma	CD
Unkown	level4f211	407.2335	7.0	4.79E+04	6.20E+04	9.39E+06	7.21E+04	1.26E+07	4.43E+06	6.08E+06	137.40	plasma	XCMS
Unkown	level4f212	410.6236	9.5	2.46E+06	4.46E+06	4.29E+06	4.48E+06	4.60E+06	4.06E+06	9.00E+05	22.16	plasma	LDA
Unkown	level4f212	410.6244	9.5	7.36E+05	2.29E+06	2.33E+06	2.15E+06	2.20E+06	1.94E+06	6.78E+05	34.91	plasma	CD
Unkown	level4f213	410.8575	11.6	4.24E+06	4.94E+06	5.60E+06	4.95E+06	2.64E+05	4.00E+06	2.14E+06	53.59	plasma	LDA
Unkown	level4f213	410.8569	11.6	1.60E+06	1.97E+06	2.13E+06	1.97E+06	1.84E+06	1.90E+06	1.95E+05	10.23	plasma	CD

Unkown	level4f213	410.8567	11.7	1.04E+06	1.27E+06	1.42E+06	1.28E+06	1.26E+06	1.25E+06	1.34E+05	10.72	plasma	XCMS
Unkown	level4f214	412.6226	9.5	1.98E+06	3.35E+06	3.62E+06	3.26E+06	3.81E+06	3.21E+06	7.17E+05	22.37	plasma	LDA
Unkown	level4f214	412.6226	9.5	6.22E+05	2.84E+05	9.95E+05	5.96E+05	1.25E+06	7.48E+05	3.75E+05	50.14	plasma	CD
Unkown	level4f215	414.7620	2.6	3.71E+06	5.58E+06	4.00E+06	3.66E+06	7.22E+06	4.83E+06	1.55E+06	32.06	plasma	LDA
Unkown	level4f215	414.7620	2.8	4.29E+05	6.05E+05	5.02E+05	4.09E+05	7.28E+05	5.34E+05	1.33E+05	24.82	plasma	CD
Unkown	level4f215	414.7621	2.8	9.75E+05	1.19E+06	9.43E+05	9.51E+05	8.31E+05	9.77E+05	1.30E+05	13.31	plasma	XCMS
Unkown	level4f216	420.8867	11.8	2.28E+07	4.04E+07	3.49E+07	3.02E+07	3.18E+07	3.20E+07	6.47E+06	20.21	plasma	LDA
Unkown	level4f216	420.8860	11.7	3.43E+06	7.14E+06	8.00E+06	6.83E+06	6.94E+06	6.47E+06	1.76E+06	27.23	plasma	CD
Unkown	level4f216	420.8859	11.7	5.54E+06	5.28E+06	5.64E+06	5.46E+06	5.32E+06	5.45E+06	1.49E+05	2.73	plasma	XCMS
Unkown	level4f217	424.3430	4.0	6.78E+06	4.88E+06	4.49E+06	5.41E+06	7.51E+06	5.81E+06	1.28E+06	22.08	plasma	LDA
Unkown	level4f217	424.3427	4.0	1.92E+06	1.34E+06	1.14E+06	1.51E+06	2.31E+06	1.64E+06	4.71E+05	28.64	plasma	CD
Unkown	level4f217	424.3425	4.0	1.82E+06	1.37E+06	1.19E+06	1.53E+06	2.04E+06	1.59E+06	3.44E+05	21.63	plasma	XCMS
Unkown	level4f218	426.3588	3.9	2.69E+07	1.99E+07	2.19E+07	2.62E+07	3.34E+07	2.57E+07	5.24E+06	20.42	plasma	LDA
Unkown	level4f218	426.3587	3.9	7.50E+06	6.16E+06	6.04E+06	7.53E+06	9.23E+06	7.29E+06	1.29E+06	17.75	plasma	CD
Unkown	level4f218	426.3585	3.9	7.17E+06	5.38E+06	5.56E+06	6.95E+06	8.17E+06	6.65E+06	1.17E+06	17.58	plasma	XCMS
Unkown	level4f219	428.2494	2.9	1.60E+07	1.07E+07	1.05E+07	6.92E+06	1.35E+07	1.15E+07	3.41E+06	29.65	plasma	LDA
Unkown	level4f219	428.2494	2.9	2.14E+06	2.26E+06	1.35E+06	9.81E+05	8.18E+05	1.51E+06	6.59E+05	43.71	plasma	CD
Unkown	level4f219	428.2496	2.9	2.53E+06	2.29E+06	2.82E+06	2.39E+06	2.51E+06	2.51E+06	1.99E+05	7.93	plasma	XCMS
Unkown	level4f220	428.7012	11.5	7.20E+06	1.38E+07	1.37E+07	1.23E+07	1.28E+07	1.20E+07	2.72E+06	22.78	plasma	LDA
Unkown	level4f220	428.7005	11.5	1.26E+07	2.15E+07	2.13E+07	2.16E+07	2.01E+07	1.94E+07	3.89E+06	20.02	plasma	CD
Unkown	level4f220	428.7005	11.5	1.83E+06	3.41E+06	2.89E+06	2.75E+06	3.14E+06	2.80E+06	6.01E+05	21.44	plasma	XCMS
Unkown	level4f221	431.9847	14.1	5.02E+06	5.20E+06	5.60E+06	4.31E+06	4.07E+06	4.84E+06	6.33E+05	13.07	plasma	LDA
Unkown	level4f221	431.9851	14.0	1.33E+06	1.27E+06	1.49E+06	1.20E+06	6.85E+05	1.19E+06	3.04E+05	25.47	plasma	CD
Unkown	level4f221	431.9850	14.1	1.22E+06	1.18E+06	1.42E+06	1.10E+06	1.42E+04	9.87E+05	5.57E+05	56.39	plasma	XCMS
Unkown	level4f222	433.2841	2.6	1.77E+07	2.12E+07	1.43E+07	1.69E+07	1.49E+07	1.70E+07	2.75E+06	16.18	plasma	LDA
Unkown	level4f222	433.2839	2.6	3.73E+06	5.88E+06	4.17E+06	4.47E+06	3.64E+06	4.38E+06	9.03E+05	20.62	plasma	CD
Unkown	level4f222	433.2847	2.6	4.66E+06	6.07E+06	3.54E+06	4.03E+06	3.63E+06	4.39E+06	1.04E+06	23.71	plasma	XCMS
Unkown	level4f223	435.3462	1.9	3.66E+06	3.23E+06	2.82E+06	2.23E+06	2.13E+06	2.81E+06	6.51E+05	23.17	plasma	LDA
Unkown	level4f223	435.3462	1.9	8.62E+05	9.20E+05	7.68E+05	6.90E+05	5.13E+05	7.51E+05	1.59E+05	21.23	plasma	CD
Unkown	level4f224	436.7751	2.7	2.11E+06	3.11E+06	1.91E+06	1.94E+06	2.16E+06	2.24E+06	4.93E+05	21.97	plasma	LDA

Unkown	level4f224	436.7751	2.6	4.15E+05	1.02E+06	4.99E+05	6.28E+05	4.27E+05	5.97E+05	2.50E+05	41.86	plasma	CD
Unkown	level4f225	437.2366	2.6	3.67E+06	3.26E+06	3.50E+06	3.24E+06	3.92E+06	3.52E+06	2.89E+05	8.20	plasma	LDA
Unkown	level4f225	437.2366	2.6	9.01E+05	7.72E+05	6.17E+05	4.80E+05	6.17E+05	6.78E+05	1.62E+05	23.94	plasma	CD
Unkown	level4f225	437.2360	2.6	9.00E+05	8.14E+05	6.43E+05	3.67E+05	7.74E+05	6.99E+05	2.08E+05	29.68	plasma	XCMS
Unkown	level4f226	438.7280	11.5	3.45E+06	5.54E+06	6.63E+06	5.70E+06	6.13E+06	5.49E+06	1.21E+06	22.13	plasma	LDA
Unkown	level4f226	438.7288	11.5	5.87E+06	9.54E+06	1.13E+07	9.51E+06	9.32E+06	9.10E+06	1.97E+06	21.65	plasma	CD
Unkown	level4f226	438.7291	11.5	8.27E+05	1.08E+06	1.63E+06	1.43E+06	1.48E+06	1.29E+06	3.26E+05	25.32	plasma	XCMS
Unkown	level4f227	440.7270	11.5	1.10E+07	1.89E+07	1.95E+07	1.77E+07	1.75E+07	1.69E+07	3.42E+06	20.19	plasma	LDA
Unkown	level4f227	440.7270	11.5	5.05E+06	9.15E+06	7.90E+06	7.96E+06	8.76E+06	7.76E+06	1.61E+06	20.72	plasma	CD
Unkown	level4f227	440.7264	11.5	2.63E+06	4.62E+06	4.61E+06	4.24E+06	4.23E+06	4.07E+06	8.24E+05	20.25	plasma	XCMS
Unkown	level4f228	441.9368	14.4	4.31E+06	6.31E+06	5.53E+06	5.55E+06	3.85E+06	5.11E+06	1.01E+06	19.73	plasma	LDA
Unkown	level4f228	441.9368	14.4	1.14E+06	1.61E+06	1.44E+06	1.42E+06	9.87E+05	1.32E+06	2.52E+05	19.08	plasma	CD
Unkown	level4f228	441.9367	14.4	7.01E+05	1.42E+06	1.39E+06	1.38E+06	9.93E+05	1.18E+06	3.18E+05	27.06	plasma	XCMS
Unkown	level4f229	446.2605	2.6	4.31E+07	3.44E+07	2.45E+07	2.44E+07	1.87E+07	2.90E+07	9.67E+06	33.31	plasma	LDA
Unkown	level4f229	446.2609	3.0	4.82E+06	5.97E+06	3.53E+06	5.57E+06	3.10E+06	4.60E+06	1.25E+06	27.17	plasma	CD
Unkown	level4f230	453.2099	2.6	4.15E+06	2.78E+06	2.33E+06	3.76E+06	2.41E+06	3.09E+06	8.23E+05	26.69	plasma	LDA
Unkown	level4f230	453.2099	2.6	9.01E+05	4.89E+05	4.96E+05	4.40E+05	2.58E+05	5.17E+05	2.35E+05	45.56	plasma	CD
Unkown	level4f230	453.2094	2.6	7.86E+05	3.69E+05	5.05E+05	5.65E+05	4.40E+05	5.33E+05	1.59E+05	29.92	plasma	XCMS
Unkown	level4f231	453.2099	3.0	5.15E+06	2.97E+06	2.80E+06	3.80E+06	3.72E+06	3.69E+06	9.29E+05	25.20	plasma	LDA
Unkown	level4f231	453.2102	3.0	1.72E+06	3.80E+05	6.22E+05	7.35E+05	8.14E+05	8.54E+05	5.11E+05	59.78	plasma	CD
Unkown	level4f231	453.2099	3.0	1.28E+06	5.96E+05	7.15E+05	8.30E+05	1.02E+06	8.87E+05	2.68E+05	30.25	plasma	XCMS
Unkown	level4f232	459.2792	2.6	6.36E+06	8.24E+06	6.47E+06	6.70E+06	6.29E+06	6.81E+06	8.10E+05	11.89	plasma	LDA
Unkown	level4f232	459.2792	2.6	1.78E+06	2.15E+06	1.97E+06	1.41E+06	1.71E+06	1.81E+06	2.81E+05	15.57	plasma	CD
Unkown	level4f232	459.2804	2.6	1.02E+06	1.87E+06	1.61E+06	1.33E+06	1.42E+06	1.45E+06	3.18E+05	21.88	plasma	XCMS
Unkown	level4f233	468.3100	8.1	4.20E+07	2.37E+07	2.52E+07	3.68E+07	6.40E+07	3.83E+07	1.63E+07	42.44	plasma	LDA
Unkown	level4f233	468.3101	8.2	9.32E+06	9.57E+06	6.81E+06	5.85E+06	8.69E+06	8.05E+06	1.64E+06	20.36	plasma	CD
Unkown	level4f233	468.3097	8.0	8.66E+06	6.33E+06	7.18E+06	5.49E+06	7.40E+06	7.01E+06	1.19E+06	16.98	plasma	XCMS
Unkown	level4f234	472.2762	2.9	2.85E+07	2.58E+07	3.01E+07	2.50E+07	2.00E+07	2.59E+07	3.86E+06	14.91	plasma	LDA
Unkown	level4f234	472.2762	3.2	8.49E+06	8.32E+06	9.33E+06	9.16E+06	8.28E+06	8.71E+06	4.95E+05	5.68	plasma	CD
Unkown	level4f234	472.2761	3.2	7.30E+06	7.70E+06	7.44E+06	9.21E+06	7.09E+06	7.75E+06	8.44E+05	10.90	plasma	XCMS

Unkown	level4f235	478.2948	7.8	1.49E+07	3.25E+07	1.40E+07	1.83E+07	1.58E+07	1.91E+07	7.67E+06	40.13	plasma	LDA
Unkown	level4f235	478.2942	7.7	3.89E+06	4.76E+06	4.39E+06	2.99E+06	4.54E+06	4.11E+06	7.07E+05	17.20	plasma	CD
Unkown	level4f235	478.2940	7.7	3.83E+06	4.26E+06	3.29E+06	3.84E+06	3.48E+06	3.74E+06	3.73E+05	9.96	plasma	XCMS
Unkown	level4f236	478.3301	8.3	5.86E+06	4.43E+06	3.04E+06	5.47E+06	7.02E+06	5.16E+06	1.50E+06	29.12	plasma	LDA
Unkown	level4f236	478.3301	8.2	8.91E+05	5.60E+05	7.44E+05	5.83E+05	2.99E+05	6.15E+05	2.22E+05	36.05	plasma	CD
Unkown	level4f236	478.3299	8.2	1.12E+06	7.87E+05	8.92E+05	3.22E+06	1.27E+06	1.46E+06	1.00E+06	68.94	plasma	XCMS
Unkown	level4f237	480.3094	8.1	5.18E+06	5.48E+06	5.67E+06	5.29E+06	5.37E+06	5.40E+06	1.89E+05	3.50	plasma	LDA
Unkown	level4f237	480.3095	8.1	9.45E+05	1.40E+06	1.21E+06	1.61E+06	1.33E+06	1.30E+06	2.46E+05	18.92	plasma	CD
Unkown	level4f237	480.3094	8.1	1.46E+06	1.49E+06	1.38E+06	1.13E+06	1.18E+06	1.33E+06	1.64E+05	12.32	plasma	XCMS
Unkown	level4f238	480.3097	7.5	6.24E+06	5.79E+06	5.33E+06	4.97E+06	5.06E+06	5.48E+06	5.34E+05	9.75	plasma	LDA
Unkown	level4f238	480.3096	7.5	5.38E+05	1.57E+06	1.03E+06	5.55E+05	6.95E+05	8.77E+05	4.33E+05	49.44	plasma	CD
Unkown	level4f238	480.3095	7.5	9.97E+05	1.51E+06	1.32E+06	1.13E+06	1.20E+06	1.23E+06	1.93E+05	15.69	plasma	XCMS
Unkown	level4f239	480.3450	6.9	6.13E+06	4.61E+06	4.51E+06	4.65E+06	5.79E+06	5.14E+06	7.63E+05	14.85	plasma	LDA
Unkown	level4f239	480.3450	6.9	1.72E+06	1.01E+06	8.86E+05	9.60E+05	1.09E+06	1.13E+06	3.35E+05	29.55	plasma	CD
Unkown	level4f239	480.3453	6.8	1.54E+06	1.13E+06	1.03E+06	1.15E+06	8.79E+05	1.15E+06	2.46E+05	21.45	plasma	XCMS
Unkown	level4f240	480.3455	7.6	3.02E+06	3.66E+06	3.24E+06	3.71E+06	3.78E+06	3.48E+06	3.32E+05	9.52	plasma	LDA
Unkown	level4f240	480.3455	7.8	7.04E+05	8.04E+05	5.65E+05	6.11E+05	1.02E+06	7.41E+05	1.82E+05	24.55	plasma	CD
Unkown	level4f240	480.3456	7.5	7.89E+05	8.81E+05	1.32E+06	1.35E+06	9.63E+05	1.06E+06	2.56E+05	24.15	plasma	XCMS
Unkown	level4f241	481.2628	2.6	3.08E+06	2.89E+06	2.54E+06	2.42E+06	2.58E+06	2.70E+06	2.74E+05	10.16	plasma	LDA
Unkown	level4f241	481.2628	2.6	1.05E+06	9.82E+05	6.70E+05	6.22E+05	6.14E+05	7.88E+05	2.12E+05	26.93	plasma	CD
Unkown	level4f241	481.2617	2.6	4.75E+05	7.75E+05	5.68E+05	6.80E+05	5.72E+05	6.14E+05	1.16E+05	18.81	plasma	XCMS
Unkown	level4f242	486.5535	9.5	8.49E+05	1.92E+06	1.72E+06	1.64E+06	1.73E+06	1.57E+06	4.18E+05	26.58	plasma	LDA
Unkown	level4f242	486.5535	9.5	2.98E+05	7.13E+05	6.62E+05	5.90E+05	6.27E+05	5.78E+05	1.63E+05	28.17	plasma	CD
Unkown	level4f243	488.6570	11.5	9.45E+06	1.59E+07	1.61E+07	1.49E+07	1.50E+07	1.43E+07	2.74E+06	19.23	plasma	LDA
Unkown	level4f243	488.6563	11.5	5.11E+06	8.74E+06	8.83E+06	8.29E+06	9.70E+06	8.14E+06	1.76E+06	21.69	plasma	CD
Unkown	level4f243	488.6563	11.5	2.21E+06	3.92E+06	3.45E+06	3.40E+06	3.70E+06	3.34E+06	6.62E+05	19.82	plasma	XCMS
Unkown	level4f244	488.8741	11.8	1.16E+07	1.44E+07	1.39E+07	1.28E+07	1.23E+07	1.30E+07	1.14E+06	8.79	plasma	LDA
Unkown	level4f244	488.8731	11.7	2.07E+06	3.37E+06	3.47E+06	4.04E+06	3.06E+06	3.20E+06	7.23E+05	22.58	plasma	CD
Unkown	level4f244	488.8731	11.7	2.10E+06	2.66E+06	2.69E+06	2.30E+06	2.46E+06	2.44E+06	2.48E+05	10.14	plasma	XCMS
Unkown	level4f245	490.2863	2.5	1.00E+07	2.00E+07	1.47E+07	1.09E+07	1.20E+07	1.35E+07	4.04E+06	29.90	plasma	LDA

Unkown	level4f245	490.2863	2.6	2.81E+06	5.91E+06	4.47E+06	2.94E+06	3.11E+06	3.85E+06	1.33E+06	34.65	plasma	CD
Unkown	level4f246	490.2863	3.0	1.05E+07	1.05E+07	8.20E+06	9.35E+06	6.96E+06	9.11E+06	1.54E+06	16.86	plasma	LDA
Unkown	level4f246	490.2863	3.0	2.49E+06	2.40E+06	1.76E+06	1.97E+06	1.40E+06	2.00E+06	4.52E+05	22.58	plasma	CD
Unkown	level4f246	490.2862	3.0	2.73E+06	2.79E+06	2.61E+06	2.41E+06	1.78E+06	2.46E+06	4.08E+05	16.54	plasma	XCMS
Unkown	level4f247	490.3727	1.9	6.79E+06	5.33E+06	5.74E+06	4.52E+06	5.48E+06	5.57E+06	8.17E+05	14.67	plasma	LDA
Unkown	level4f247	490.3727	1.9	1.64E+06	1.28E+06	1.48E+06	4.49E+05	1.71E+06	1.31E+06	5.10E+05	38.87	plasma	CD
Unkown	level4f248	494.3268	8.4	5.59E+07	5.25E+07	5.17E+07	5.57E+07	5.12E+07	5.34E+07	2.25E+06	4.22	plasma	LDA
Unkown	level4f248	494.3266	8.4	1.48E+07	1.47E+07	1.45E+07	1.43E+07	8.24E+06	1.33E+07	2.84E+06	21.35	plasma	CD
Unkown	level4f248	494.3263	8.3	1.35E+07	1.28E+07	1.20E+07	1.20E+07	1.22E+07	1.25E+07	6.52E+05	5.22	plasma	XCMS
Unkown	level4f249	497.2363	2.6	4.12E+06	2.73E+06	3.37E+06	2.81E+06	3.52E+06	3.31E+06	5.68E+05	17.17	plasma	LDA
Unkown	level4f249	497.2363	3.1	7.30E+05	3.37E+05	2.33E+05	3.51E+05	2.56E+05	3.81E+05	2.01E+05	52.75	plasma	CD
Unkown	level4f249	497.2362	2.7	7.26E+05	5.14E+05	5.92E+05	5.04E+05	5.92E+05	5.86E+05	8.90E+04	15.19	plasma	XCMS
Unkown	level4f250	498.3471	7.8	1.21E+08	1.17E+08	1.03E+08	1.15E+08	1.09E+08	1.13E+08	6.82E+06	6.02	plasma	LDA
Unkown	level4f250	498.3471	7.6	1.40E+07	1.41E+07	1.72E+07	1.66E+07	1.86E+07	1.61E+07	2.03E+06	12.59	plasma	CD
Unkown	level4f250	498.3470	7.6	1.43E+07	1.28E+07	1.03E+07	1.24E+07	1.19E+07	1.24E+07	1.44E+06	11.68	plasma	XCMS
Unkown	level4f251	498.6847	11.5	4.00E+06	7.08E+06	7.54E+06	6.08E+06	6.12E+06	6.16E+06	1.36E+06	22.10	plasma	LDA
Unkown	level4f251	498.6846	11.5	2.09E+06	3.72E+06	3.48E+06	3.58E+06	3.02E+06	3.18E+06	6.61E+05	20.79	plasma	CD
Unkown	level4f251	498.6848	11.5	9.20E+05	1.75E+06	1.84E+06	1.50E+06	1.51E+06	1.50E+06	3.57E+05	23.79	plasma	XCMS
Unkown	level4f252	499.2754	2.9	5.73E+06	5.58E+06	8.76E+06	4.61E+06	5.67E+06	6.07E+06	1.57E+06	25.87	plasma	LDA
Unkown	level4f252	499.2760	3.3	1.53E+07	1.08E+07	8.72E+06	1.37E+07	1.21E+07	1.21E+07	2.55E+06	21.02	plasma	CD
Unkown	level4f252	499.2748	3.3	1.14E+06	1.09E+06	8.58E+05	1.01E+06	9.08E+05	1.00E+06	1.19E+05	11.92	plasma	XCMS
Unkown	level4f253	499.9720	14.1	3.64E+06	3.80E+06	4.40E+06	4.15E+06	3.37E+06	3.87E+06	4.08E+05	10.54	plasma	LDA
Unkown	level4f253	499.9724	14.0	8.17E+05	1.08E+06	1.17E+06	1.02E+06	8.83E+05	9.93E+05	1.43E+05	14.36	plasma	CD
Unkown	level4f253	499.9726	14.0	9.09E+05	1.25E+04	1.13E+06	1.06E+06	1.39E+04	6.26E+05	5.65E+05	90.28	plasma	XCMS
Unkown	level4f254	501.9860	13.6	2.94E+06	3.11E+06	3.10E+06	2.69E+06	2.47E+06	2.86E+06	2.78E+05	9.74	plasma	LDA
Unkown	level4f254	501.9865	13.5	7.63E+05	8.46E+05	8.08E+05	7.25E+05	6.74E+05	7.63E+05	6.76E+04	8.86	plasma	CD
Unkown	level4f254	501.9868	13.5	7.80E+05	2.37E+05	4.90E+05	6.71E+05	5.83E+05	5.52E+05	2.06E+05	37.35	plasma	XCMS
Unkown	level4f255	502.2939	7.3	9.13E+06	8.66E+06	8.91E+06	1.42E+06	1.13E+07	7.88E+06	3.76E+06	47.68	plasma	LDA
Unkown	level4f255	502.2934	7.4	1.59E+06	2.29E+06	2.58E+06	2.54E+06	3.19E+06	2.44E+06	5.80E+05	23.80	plasma	CD
Unkown	level4f255	502.2936	7.4	2.21E+06	2.36E+06	2.57E+06	3.03E+06	2.62E+06	2.56E+06	3.11E+05	12.17	plasma	XCMS

Unkown	level4f256	502.2931	8.0	7.66E+06	9.43E+06	7.48E+06	9.93E+06	1.00E+07	8.90E+06	1.24E+06	13.89	plasma	LDA
Unkown	level4f256	502.2934	8.0	1.95E+06	2.40E+06	2.85E+06	2.94E+06	3.29E+06	2.69E+06	5.22E+05	19.42	plasma	CD
Unkown	level4f256	502.2935	8.0	1.83E+06	2.41E+06	2.10E+06	2.36E+06	2.48E+06	2.24E+06	2.69E+05	12.04	plasma	XCMS
Unkown	level4f257	503.3065	3.1	4.93E+06	5.35E+06	4.23E+06	4.80E+06	4.40E+06	4.74E+06	4.42E+05	9.33	plasma	LDA
Unkown	level4f257	503.3076	3.1	3.08E+07	3.27E+07	3.36E+07	3.23E+07	2.51E+07	3.09E+07	3.40E+06	11.02	plasma	CD
Unkown	level4f257	503.3066	2.6	2.15E+06	2.67E+06	2.43E+06	2.73E+06	8.95E+05	2.17E+06	7.50E+05	34.52	plasma	XCMS
Unkown	level4f258	503.3065	2.6	9.87E+06	1.04E+07	9.25E+06	1.06E+07	8.75E+06	9.79E+06	7.94E+05	8.11	plasma	LDA
Unkown	level4f258	503.3078	2.6	6.19E+07	3.54E+07	4.48E+07	5.18E+07	5.40E+07	4.96E+07	1.00E+07	20.20	plasma	CD
Unkown	level4f258	503.3066	2.6	2.15E+06	2.67E+06	2.43E+06	2.73E+06	8.95E+05	2.17E+06	7.50E+05	34.52	plasma	XCMS
Unkown	level4f259	503.4078	1.9	6.35E+06	5.82E+06	5.32E+06	7.51E+06	5.94E+06	6.19E+06	8.27E+05	13.37	plasma	LDA
Unkown	level4f259	503.4078	1.9	1.09E+06	1.31E+06	9.18E+05	6.32E+05	5.93E+05	9.08E+05	3.04E+05	33.47	plasma	CD
Unkown	level4f260	505.4247	1.9	3.75E+06	3.40E+06	3.76E+06	3.77E+06	2.74E+06	3.49E+06	4.44E+05	12.75	plasma	LDA
Unkown	level4f260	505.4247	2.0	5.91E+05	4.38E+05	6.21E+05	3.03E+05	3.08E+05	4.52E+05	1.51E+05	33.29	plasma	CD
Unkown	level4f261	506.3681	1.9	2.84E+06	2.90E+06	2.58E+06	2.33E+06	3.07E+06	2.74E+06	2.90E+05	10.58	plasma	LDA
Unkown	level4f261	506.3682	2.0	6.85E+05	4.94E+05	4.97E+05	5.93E+05	6.24E+05	5.79E+05	8.28E+04	14.31	plasma	CD
Unkown	level4f262	509.3550	2.0	2.39E+06	2.18E+06	2.08E+06	2.59E+06	3.33E+06	2.52E+06	4.97E+05	19.78	plasma	LDA
Unkown	level4f262	509.3559	2.0	7.93E+05	8.53E+05	7.01E+05	1.25E+06	1.04E+06	9.27E+05	2.19E+05	23.64	plasma	CD
Unkown	level4f262	509.3560	2.0	5.43E+05	4.60E+05	4.50E+05	5.96E+05	6.63E+05	5.42E+05	9.06E+04	16.71	plasma	XCMS
Unkown	level4f263	509.9236	14.4	1.84E+06	3.11E+06	2.54E+06	2.32E+06	1.68E+06	2.30E+06	5.72E+05	24.90	plasma	LDA
Unkown	level4f263	509.9236	14.4	5.48E+05	8.13E+05	6.97E+05	6.37E+05	4.55E+05	6.30E+05	1.37E+05	21.79	plasma	CD
Unkown	level4f263	509.9239	14.4	4.46E+05	5.88E+05	6.56E+05	6.05E+05	4.33E+05	5.46E+05	1.00E+05	18.38	plasma	XCMS
Unkown	level4f264	510.3562	7.5	1.43E+07	1.46E+07	1.19E+07	1.76E+07	1.78E+07	1.52E+07	2.48E+06	16.26	plasma	LDA
Unkown	level4f264	510.3566	7.5	3.75E+06	3.32E+06	3.68E+06	4.02E+06	5.94E+06	4.14E+06	1.03E+06	24.95	plasma	CD
Unkown	level4f264	510.3564	7.4	3.49E+06	5.26E+06	3.33E+06	3.01E+06	2.50E+06	3.52E+06	1.05E+06	29.76	plasma	XCMS
Unkown	level4f265	516.3020	2.8	3.36E+07	4.26E+07	3.99E+07	4.70E+07	2.94E+07	3.85E+07	7.02E+06	18.24	plasma	LDA
Unkown	level4f265	516.3023	2.9	1.13E+07	1.03E+07	1.12E+07	1.27E+07	1.07E+07	1.13E+07	9.19E+05	8.16	plasma	CD
Unkown	level4f265	516.3026	2.9	8.27E+06	7.83E+06	7.87E+06	8.49E+06	5.87E+06	7.66E+06	1.04E+06	13.61	plasma	XCMS
Unkown	level4f266	516.3023	3.3	5.62E+07	4.04E+07	4.62E+07	4.94E+07	4.07E+07	4.66E+07	6.60E+06	14.16	plasma	LDA
Unkown	level4f266	516.3024	3.3	1.47E+07	9.34E+06	7.98E+06	1.31E+07	1.18E+07	1.14E+07	2.74E+06	24.10	plasma	CD
Unkown	level4f266	516.3024	3.3	1.25E+07	9.14E+06	1.17E+07	1.25E+07	1.09E+07	1.13E+07	1.40E+06	12.38	plasma	XCMS

Unkown	level4f267	520.3335	2.6	1.93E+08	1.63E+08	1.56E+08	1.63E+08	1.47E+08	1.64E+08	1.74E+07	10.59	plasma	LDA
Unkown	level4f267	520.3339	2.7	5.92E+07	3.22E+07	4.18E+07	4.80E+07	5.15E+07	4.66E+07	1.02E+07	21.90	plasma	CD
Unkown	level4f267	520.3346	2.6	4.57E+07	3.63E+07	3.82E+07	3.95E+07	3.65E+07	3.92E+07	3.84E+06	9.77	plasma	XCMS
Unkown	level4f268	520.3344	3.1	1.19E+08	1.13E+08	1.04E+08	1.08E+08	9.93E+07	1.09E+08	7.64E+06	7.04	plasma	LDA
Unkown	level4f268	520.3344	3.1	2.95E+07	3.12E+07	3.23E+07	3.09E+07	2.39E+07	2.96E+07	3.29E+06	11.14	plasma	CD
Unkown	level4f268	520.3339	3.1	2.92E+07	2.52E+07	1.81E+07	2.57E+07	2.35E+07	2.43E+07	4.07E+06	16.76	plasma	XCMS
Unkown	level4f269	520.3415	7.3	2.36E+08	2.47E+08	3.07E+08	1.25E+09	1.45E+09	6.98E+08	6.00E+08	85.93	plasma	LDA
Unkown	level4f269	520.3414	7.5	2.19E+08	2.94E+08	2.06E+08	2.72E+08	2.59E+08	2.50E+08	3.67E+07	14.66	plasma	CD
Unkown	level4f269	520.3415	7.5	1.23E+08	2.17E+08	1.46E+08	2.26E+08	2.32E+08	1.89E+08	5.05E+07	26.72	plasma	XCMS
Unkown	level4f270	521.3381	2.8	3.62E+07	3.05E+07	2.61E+07	2.66E+07	2.50E+07	2.89E+07	4.61E+06	15.98	plasma	LDA
Unkown	level4f270	521.3381	2.7	5.51E+06	4.60E+06	3.85E+06	4.22E+06	2.85E+06	4.21E+06	9.77E+05	23.23	plasma	CD
Unkown	level4f270	521.3367	3.1	3.84E+06	3.32E+06	3.04E+06	2.67E+06	2.80E+06	3.13E+06	4.65E+05	14.84	plasma	XCMS
Unkown	level4f271	521.4205	1.9	4.40E+07	3.67E+07	3.18E+07	2.72E+07	2.58E+07	3.31E+07	7.44E+06	22.47	plasma	LDA
Unkown	level4f271	521.4209	2.0	1.13E+07	9.09E+06	9.26E+06	6.53E+06	4.19E+06	8.08E+06	2.76E+06	34.12	plasma	CD
Unkown	level4f272	524.3721	7.2	2.16E+08	6.02E+08	5.25E+08	1.25E+08	6.48E+08	4.23E+08	2.37E+08	56.01	plasma	LDA
Unkown	level4f272	524.3719	7.3	1.41E+08	1.29E+08	1.59E+08	1.41E+08	1.15E+08	1.37E+08	1.64E+07	11.94	plasma	CD
Unkown	level4f272	524.3720	7.3	1.09E+08	1.01E+08	9.11E+07	1.00E+08	1.01E+08	1.00E+08	6.22E+06	6.21	plasma	XCMS
Unkown	level4f273	524.3718	8.1	3.82E+08	3.86E+08	3.79E+08	5.08E+08	6.30E+08	4.57E+08	1.11E+08	24.31	plasma	LDA
Unkown	level4f273	524.3720	8.0	1.37E+08	1.07E+08	1.11E+08	1.26E+08	1.42E+08	1.25E+08	1.55E+07	12.43	plasma	CD
Unkown	level4f273	524.3719	7.9	8.93E+07	7.04E+07	8.10E+07	9.10E+07	1.06E+08	8.75E+07	1.30E+07	14.83	plasma	XCMS
Unkown	level4f274	526.2923	7.9	3.16E+06	3.27E+06	3.30E+06	3.27E+06	4.36E+06	3.47E+06	5.00E+05	14.41	plasma	LDA
Unkown	level4f274	526.2923	7.9	4.79E+05	6.49E+05	1.03E+06	1.10E+06	1.08E+06	8.67E+05	2.84E+05	32.80	plasma	CD
Unkown	level4f274	526.2926	7.9	7.87E+05	6.08E+05	8.75E+05	8.87E+05	9.85E+05	8.29E+05	1.42E+05	17.12	plasma	XCMS
Unkown	level4f275	531.8721	2.0	2.26E+06	1.61E+06	1.87E+06	2.65E+06	3.46E+06	2.37E+06	7.25E+05	30.53	plasma	LDA
Unkown	level4f275	531.8721	2.0	8.19E+05	5.25E+05	5.35E+05	6.88E+05	9.55E+05	7.05E+05	1.85E+05	26.28	plasma	CD
Unkown	level4f275	531.8712	2.0	4.71E+05	3.44E+05	3.99E+05	4.93E+05	7.98E+05	5.01E+05	1.76E+05	35.16	plasma	XCMS
Unkown	level4f276	534.2968	7.6	4.38E+07	5.50E+07	9.05E+07	7.15E+07	4.92E+07	6.20E+07	1.90E+07	30.65	plasma	LDA
Unkown	level4f276	534.2969	7.7	4.14E+06	1.30E+07	2.66E+07	1.54E+07	8.11E+06	1.34E+07	8.53E+06	63.49	plasma	CD
Unkown	level4f276	534.2966	7.6	7.13E+06	1.37E+07	2.11E+07	9.96E+06	5.09E+06	1.14E+07	6.31E+06	55.41	plasma	XCMS
Unkown	level4f277	534.3126	3.1	5.68E+06	5.80E+06	4.83E+06	5.23E+06	4.31E+06	5.17E+06	6.16E+05	11.91	plasma	LDA

Unkown	level4f277	534.3126	3.1	2.15E+06	1.67E+06	1.27E+06	1.48E+06	1.18E+06	1.55E+06	3.85E+05	24.82	plasma	CD
Unkown	level4f277	534.3121	3.1	1.48E+06	1.55E+06	1.15E+06	1.43E+06	1.09E+06	1.34E+06	2.07E+05	15.39	plasma	XCMS
Unkown	level4f278	534.3997	1.9	9.36E+06	6.22E+06	5.71E+06	7.72E+06	6.55E+06	7.11E+06	1.46E+06	20.49	plasma	LDA
Unkown	level4f278	534.3996	1.9	2.75E+06	1.70E+06	1.11E+06	2.09E+06	1.63E+06	1.85E+06	6.09E+05	32.82	plasma	CD
Unkown	level4f279	542.3237	8.2	1.33E+07	4.63E+06	7.39E+06	8.42E+06	1.00E+07	8.74E+06	3.20E+06	36.56	plasma	LDA
Unkown	level4f279	542.3239	8.2	2.38E+06	1.32E+06	1.28E+06	2.61E+06	3.32E+06	2.18E+06	8.74E+05	40.06	plasma	CD
Unkown	level4f279	542.3233	8.1	2.18E+06	1.23E+06	8.01E+05	2.05E+06	2.77E+06	1.81E+06	7.88E+05	43.61	plasma	XCMS
Unkown	level4f280	543.3019	2.9	3.98E+06	7.92E+06	3.71E+06	3.46E+06	3.31E+06	4.48E+06	1.94E+06	43.39	plasma	LDA
Unkown	level4f280	543.3019	2.9	3.26E+05	7.88E+05	2.80E+05	1.63E+05	1.73E+05	3.46E+05	2.57E+05	74.24	plasma	CD
Unkown	level4f281	543.3015	3.3	6.73E+06	6.44E+06	4.85E+06	5.90E+06	5.81E+06	5.95E+06	7.24E+05	12.17	plasma	LDA
Unkown	level4f281	543.3022	3.3	2.38E+07	1.82E+07	2.08E+07	1.71E+07	1.93E+07	1.99E+07	2.56E+06	12.91	plasma	CD
Unkown	level4f281	543.3014	3.3	1.56E+06	1.82E+06	1.38E+06	1.74E+06	1.53E+06	1.61E+06	1.73E+05	10.78	plasma	XCMS
Unkown	level4f282	544.3408	7.0	6.09E+07	1.90E+08	1.90E+08	5.37E+07	3.85E+07	1.07E+08	7.67E+07	71.88	plasma	LDA
Unkown	level4f282	544.3407	7.3	3.88E+07	2.04E+07	4.69E+07	3.02E+07	4.58E+07	3.64E+07	1.12E+07	30.63	plasma	CD
Unkown	level4f282	544.3410	7.2	2.45E+07	3.98E+07	3.67E+07	3.53E+07	3.97E+07	3.52E+07	6.31E+06	17.93	plasma	XCMS
Unkown	level4f283	544.3406	8.0	1.26E+08	1.08E+08	1.14E+08	1.39E+08	1.99E+08	1.37E+08	3.67E+07	26.73	plasma	LDA
Unkown	level4f283	544.3408	8.0	3.62E+07	3.44E+07	3.26E+07	3.88E+07	4.34E+07	3.71E+07	4.21E+06	11.34	plasma	CD
Unkown	level4f283	544.3409	7.9	2.91E+07	2.72E+07	2.66E+07	3.15E+07	3.76E+07	3.04E+07	4.47E+06	14.70	plasma	XCMS
Unkown	level4f284	545.3444	7.7	2.16E+07	2.00E+07	2.04E+07	2.81E+07	3.18E+07	2.44E+07	5.28E+06	21.66	plasma	LDA
Unkown	level4f284	545.3444	7.5	8.58E+05	4.96E+05	7.88E+05	1.23E+06	6.51E+06	1.97E+06	2.55E+06	128.93	plasma	CD
Unkown	level4f284	545.3441	7.9	6.03E+06	4.91E+06	5.72E+06	6.92E+06	8.24E+06	6.37E+06	1.27E+06	19.98	plasma	XCMS
Unkown	level4f285	546.3558	7.7	3.55E+07	2.82E+07	2.94E+07	3.71E+07	4.67E+07	3.54E+07	7.41E+06	20.96	plasma	LDA
Unkown	level4f285	546.3561	7.8	9.83E+06	8.35E+06	8.86E+06	1.01E+07	6.60E+06	8.74E+06	1.39E+06	15.87	plasma	CD
Unkown	level4f285	546.3561	8.0	7.10E+06	6.14E+06	6.88E+06	8.64E+06	1.06E+07	7.87E+06	1.78E+06	22.62	plasma	XCMS
Unkown	level4f286	547.3198	4.8	3.53E+07	2.05E+07	3.52E+07	1.69E+07	1.17E+07	2.39E+07	1.08E+07	45.23	plasma	LDA
Unkown	level4f286	547.3202	5.3	2.08E+06	3.81E+06	1.96E+06	4.65E+06	2.09E+06	2.92E+06	1.24E+06	42.38	plasma	CD
Unkown	level4f286	547.3200	4.7	4.30E+06	5.01E+06	4.10E+06	3.22E+06	1.95E+06	3.72E+06	1.17E+06	31.61	plasma	XCMS
Unkown	level4f287	547.3323	3.1	3.17E+06	3.03E+06	2.76E+06	2.76E+06	2.99E+06	2.94E+06	1.80E+05	6.12	plasma	LDA
Unkown	level4f287	547.3339	3.1	2.99E+07	3.25E+07	2.87E+07	3.20E+07	2.56E+07	2.98E+07	2.79E+06	9.36	plasma	CD
Unkown	level4f288	547.3322	2.6	5.84E+06	7.55E+06	7.51E+06	6.26E+06	6.75E+06	6.78E+06	7.55E+05	11.13	plasma	LDA

Unkown	level4f288	547.3340	2.7	4.71E+07	3.59E+07	4.33E+07	4.42E+07	2.01E+07	3.81E+07	1.09E+07	28.53	plasma	CD
Unkown	level4f288	547.3327	2.6	1.05E+06	1.76E+06	1.71E+06	2.46E+06	1.62E+06	1.72E+06	5.03E+05	29.27	plasma	XCMS
Unkown	level4f289	550.3946	1.9	3.94E+06	3.31E+06	3.62E+06	3.51E+06	3.20E+06	3.52E+06	2.88E+05	8.20	plasma	LDA
Unkown	level4f289	550.3946	2.0	7.58E+05	6.79E+05	8.21E+05	1.05E+06	9.68E+05	8.56E+05	1.53E+05	17.88	plasma	CD
Unkown	level4f290	558.2969	7.7	1.35E+07	1.16E+07	1.32E+07	1.46E+07	7.56E+06	1.21E+07	2.75E+06	22.75	plasma	LDA
Unkown	level4f290	558.2963	7.7	3.82E+06	3.89E+06	3.04E+06	3.82E+06	6.84E+05	3.05E+06	1.37E+06	44.86	plasma	CD
Unkown	level4f290	558.2963	7.5	1.93E+06	3.06E+06	2.67E+06	3.76E+06	1.10E+06	2.51E+06	1.03E+06	40.98	plasma	XCMS
Unkown	level4f291	558.3489	2.3	7.66E+06	5.85E+06	4.05E+06	3.70E+06	2.72E+06	4.80E+06	1.96E+06	40.88	plasma	LDA
Unkown	level4f291	558.3489	2.3	2.14E+06	1.56E+06	1.19E+06	1.01E+06	7.68E+05	1.33E+06	5.35E+05	40.15	plasma	CD
Unkown	level4f292	559.4717	1.9	1.55E+07	1.11E+07	8.70E+06	8.36E+06	7.34E+06	1.02E+07	3.28E+06	32.16	plasma	LDA
Unkown	level4f292	559.4713	1.9	4.20E+06	3.29E+06	3.01E+06	2.21E+06	2.06E+06	2.95E+06	8.68E+05	29.38	plasma	CD
Unkown	level4f292	559.4708	1.9	3.90E+06	2.85E+06	2.03E+06	1.97E+06	2.57E+05	2.20E+06	1.34E+06	60.79	plasma	XCMS
Unkown	level4f293	560.3123	7.4	2.06E+07	1.30E+07	7.87E+06	1.14E+07	9.73E+06	1.25E+07	4.92E+06	39.27	plasma	LDA
Unkown	level4f293	560.3123	7.4	5.56E+06	3.49E+06	6.71E+05	3.06E+06	1.77E+06	2.91E+06	1.85E+06	63.53	plasma	CD
Unkown	level4f293	560.3121	7.4	4.39E+06	1.94E+05	7.06E+05	3.12E+06	1.84E+06	2.05E+06	1.72E+06	84.13	plasma	XCMS
Unkown	level4f294	561.3312	3.6	8.13E+06	6.35E+06	6.57E+06	7.04E+06	5.69E+06	6.76E+06	9.07E+05	13.43	plasma	LDA
Unkown	level4f294	561.3312	3.3	2.03E+06	1.55E+06	1.50E+06	2.08E+06	1.36E+06	1.70E+06	3.27E+05	19.19	plasma	CD
Unkown	level4f294	561.3313	3.3	2.05E+06	1.35E+06	1.67E+06	1.63E+06	1.60E+06	1.66E+06	2.52E+05	15.17	plasma	XCMS
Unkown	level4f295	561.4865	1.9	1.07E+07	1.45E+07	9.02E+06	8.05E+06	7.39E+06	9.93E+06	2.85E+06	28.68	plasma	LDA
Unkown	level4f295	561.4870	1.9	3.50E+06	2.11E+06	3.16E+06	2.43E+06	2.33E+06	2.71E+06	5.95E+05	21.99	plasma	CD
Unkown	level4f296	562.3281	7.5	2.24E+07	1.41E+07	3.82E+06	7.97E+06	6.58E+06	1.10E+07	7.42E+06	67.63	plasma	LDA
Unkown	level4f296	562.3281	7.4	4.20E+06	4.08E+06	1.67E+05	2.24E+06	2.29E+06	2.60E+06	1.65E+06	63.61	plasma	CD
Unkown	level4f296	562.3277	7.3	5.11E+06	1.11E+05	3.11E+05	2.27E+06	1.55E+06	1.87E+06	2.02E+06	107.86	plasma	XCMS
Unkown	level4f297	564.3597	2.7	1.86E+08	1.76E+08	1.52E+08	1.54E+08	1.49E+08	1.63E+08	1.66E+07	10.14	plasma	LDA
Unkown	level4f297	564.3601	2.9	4.59E+07	3.19E+07	3.60E+06	1.35E+06	1.77E+07	2.01E+07	1.90E+07	94.29	plasma	CD
Unkown	level4f297	564.3607	2.6	2.68E+07	2.44E+07	3.63E+07	3.80E+07	3.40E+07	3.19E+07	5.99E+06	18.78	plasma	XCMS
Unkown	level4f298	565.3633	2.9	3.72E+07	3.28E+07	3.13E+07	2.95E+07	2.62E+07	3.14E+07	4.06E+06	12.94	plasma	LDA
Unkown	level4f298	565.3629	2.7	5.68E+06	5.94E+06	4.23E+06	4.73E+06	5.32E+06	5.18E+06	7.00E+05	13.52	plasma	CD
Unkown	level4f298	565.3633	2.7	5.37E+06	4.91E+06	4.60E+06	4.20E+06	4.03E+06	4.62E+06	5.41E+05	11.71	plasma	XCMS
Unkown	level4f299	568.3406	7.1	1.26E+06	1.31E+07	1.27E+07	1.79E+07	1.95E+07	1.29E+07	7.16E+06	55.49	plasma	LDA

Unkown	level4f299	568.3405	7.3	3.08E+06	8.92E+05	2.63E+06	4.90E+06	5.77E+06	3.45E+06	1.93E+06	55.79	plasma	CD
Unkown	level4f299	568.3404	7.2	3.49E+06	3.53E+06	3.50E+06	3.81E+06	6.62E+06	4.19E+06	1.37E+06	32.59	plasma	XCMS
Unkown	level4f300	568.3406	7.8	6.00E+05	1.10E+07	1.14E+07	1.54E+07	1.86E+07	1.14E+07	6.78E+06	59.56	plasma	LDA
Unkown	level4f300	568.3406	7.9	3.54E+06	3.33E+06	3.68E+06	4.21E+06	5.41E+06	4.03E+06	8.32E+05	20.63	plasma	CD
Unkown	level4f300	568.3405	7.8	3.53E+06	2.88E+06	3.22E+06	3.92E+06	5.05E+06	3.72E+06	8.37E+05	22.49	plasma	XCMS
Unkown	level4f301	568.4276	1.8	2.36E+06	1.70E+06	2.38E+06	2.63E+06	1.95E+06	2.21E+06	3.73E+05	16.91	plasma	LDA
Unkown	level4f301	568.4276	1.8	6.54E+05	4.30E+05	6.24E+05	6.81E+05	4.93E+05	5.77E+05	1.09E+05	18.93	plasma	CD
Unkown	level4f302	575.4664	1.9	1.20E+07	9.67E+06	7.38E+06	5.96E+06	5.91E+06	8.18E+06	2.61E+06	31.96	plasma	LDA
Unkown	level4f302	575.4672	1.9	4.64E+06	3.96E+06	2.78E+06	2.57E+06	2.62E+06	3.31E+06	9.34E+05	28.18	plasma	CD
Unkown	level4f302	575.4663	1.9	2.93E+06	2.35E+06	1.62E+06	1.47E+06	1.51E+06	1.97E+06	6.42E+05	32.52	plasma	XCMS
Unkown	level4f303	577.4826	1.9	6.48E+07	5.25E+07	4.54E+07	3.33E+07	2.38E+07	4.40E+07	1.60E+07	36.45	plasma	LDA
Unkown	level4f303	577.4825	1.9	2.17E+07	1.90E+07	1.39E+07	1.13E+07	8.34E+06	1.48E+07	5.47E+06	36.89	plasma	CD
Unkown	level4f304	578.3386	3.1	3.26E+06	3.31E+06	2.63E+06	2.83E+06	2.39E+06	2.88E+06	3.99E+05	13.82	plasma	LDA
Unkown	level4f304	578.3384	3.1	9.48E+05	1.01E+06	6.85E+05	7.60E+05	6.36E+05	8.07E+05	1.63E+05	20.15	plasma	CD
Unkown	level4f304	578.3379	3.1	7.40E+05	8.65E+05	6.23E+05	7.58E+05	6.14E+05	7.20E+05	1.04E+05	14.50	plasma	XCMS
Unkown	level4f305	578.4254	1.9	7.12E+06	5.83E+06	5.87E+06	7.67E+06	6.93E+06	6.68E+06	8.06E+05	12.05	plasma	LDA
Unkown	level4f305	578.4262	1.9	2.04E+06	2.01E+06	2.11E+06	2.12E+06	2.06E+06	2.07E+06	4.80E+04	2.32	plasma	CD
Unkown	level4f306	582.2966	7.5	3.01E+06	3.32E+06	6.99E+05	1.65E+06	1.46E+06	2.03E+06	1.10E+06	54.41	plasma	LDA
Unkown	level4f306	582.2966	7.8	1.27E+05	1.06E+06	1.47E+05	4.20E+04	1.58E+04	2.79E+05	4.42E+05	158.46	plasma	CD
Unkown	level4f306	582.2955	7.3	7.87E+05	1.98E+04	6.59E+04	3.52E+05	2.72E+05	2.99E+05	3.06E+05	102.15	plasma	XCMS
Unkown	level4f307	585.2715	6.7	5.94E+06	1.28E+07	1.19E+07	1.15E+07	7.63E+06	9.96E+06	2.99E+06	30.03	plasma	LDA
Unkown	level4f307	585.2714	6.8	3.52E+06	3.44E+06	3.96E+06	2.36E+06	2.23E+06	3.10E+06	7.65E+05	24.67	plasma	CD
Unkown	level4f307	585.2712	6.7	1.81E+06	2.55E+06	1.98E+06	1.83E+06	8.57E+05	1.80E+06	6.09E+05	33.73	plasma	XCMS
Unkown	level4f308	587.3275	2.6	2.83E+06	3.35E+06	2.99E+06	3.47E+06	2.98E+06	3.13E+06	2.71E+05	8.66	plasma	LDA
Unkown	level4f308	587.3275	2.7	6.63E+05	6.42E+05	8.26E+05	7.53E+05	7.25E+05	7.22E+05	7.39E+04	10.23	plasma	CD
Unkown	level4f309	587.3269	3.3	4.83E+06	4.23E+06	4.04E+06	3.67E+06	3.66E+06	4.09E+06	4.82E+05	11.80	plasma	LDA
Unkown	level4f309	587.3286	3.3	2.83E+07	2.45E+07	2.60E+07	2.08E+07	2.18E+07	2.43E+07	3.06E+06	12.60	plasma	CD
Unkown	level4f309	587.3273	3.3	1.12E+06	9.51E+05	7.64E+05	8.74E+05	7.14E+05	8.84E+05	1.61E+05	18.22	plasma	XCMS
Unkown	level4f310	591.3581	2.6	3.84E+06	3.91E+06	3.91E+06	3.78E+06	3.60E+06	3.81E+06	1.30E+05	3.41	plasma	LDA
Unkown	level4f310	591.3581	2.6	8.61E+05	8.69E+05	1.33E+06	1.16E+06	1.00E+06	1.05E+06	2.01E+05	19.19	plasma	CD

Unkown	level4f311	575.4640	1.9	1.20E+07	9.35E+06	7.31E+06	5.96E+06	5.91E+06	8.10E+06	2.57E+06	31.76	plasma	LDA
Unkown	level4f311	575.4672	1.9	4.64E+06	3.96E+06	2.78E+06	2.57E+06	2.62E+06	3.31E+06	9.34E+05	28.18	plasma	CD
Unkown	level4f311	575.4663	1.9	2.93E+06	2.35E+06	1.62E+06	1.47E+06	1.51E+06	1.97E+06	6.42E+05	32.52	plasma	XCMS
Unkown	level4f312	594.4230	2.0	5.23E+06	4.90E+06	3.87E+06	4.12E+06	2.98E+06	4.22E+06	8.87E+05	21.03	plasma	LDA
Unkown	level4f312	594.4230	2.0	1.00E+06	9.07E+05	9.03E+05	1.05E+06	6.96E+05	9.10E+05	1.35E+05	14.78	plasma	CD
Unkown	level4f313	595.4913	1.9	2.50E+07	2.02E+07	1.54E+07	1.00E+07	8.94E+06	1.59E+07	6.78E+06	42.62	plasma	LDA
Unkown	level4f313	595.4926	1.9	2.19E+07	2.17E+07	1.56E+07	1.27E+07	9.28E+06	1.62E+07	5.56E+06	34.26	plasma	CD
Unkown	level4f314	602.3755	2.3	7.37E+06	7.29E+06	3.51E+06	3.60E+06	2.79E+06	4.91E+06	2.23E+06	45.36	plasma	LDA
Unkown	level4f314	602.3755	2.4	2.20E+06	2.06E+06	9.79E+05	1.10E+06	9.16E+05	1.45E+06	6.25E+05	43.08	plasma	CD
Unkown	level4f315	606.5701	11.5	3.04E+06	5.80E+06	5.84E+06	4.61E+06	4.94E+06	4.85E+06	1.14E+06	23.57	plasma	LDA
Unkown	level4f315	606.5706	11.5	1.12E+06	2.28E+06	2.22E+06	2.50E+06	2.67E+06	2.16E+06	6.06E+05	28.07	plasma	CD
Unkown	level4f315	606.5702	11.5	5.74E+05	1.42E+06	1.36E+06	1.11E+06	1.29E+06	1.15E+06	3.42E+05	29.75	plasma	XCMS
Unkown	level4f316	608.3873	2.7	1.53E+08	1.42E+08	1.22E+08	1.25E+08	1.08E+08	1.30E+08	1.78E+07	13.72	plasma	LDA
Unkown	level4f316	608.3870	2.7	3.08E+07	3.73E+07	3.77E+07	3.01E+07	3.43E+07	3.40E+07	3.56E+06	10.46	plasma	CD
Unkown	level4f316	608.3867	2.6	3.64E+07	1.97E+07	3.02E+07	3.09E+07	2.79E+07	2.90E+07	6.06E+06	20.89	plasma	XCMS
Unkown	level4f317	608.3868	3.2	9.38E+07	8.37E+07	7.87E+07	8.24E+07	7.32E+07	8.24E+07	7.58E+06	9.21	plasma	LDA
Unkown	level4f317	608.3870	3.2	2.99E+07	2.50E+07	2.40E+07	2.36E+07	9.26E+06	2.24E+07	7.74E+06	34.62	plasma	CD
Unkown	level4f317	608.3866	3.2	2.35E+07	2.07E+07	1.94E+07	2.04E+07	1.87E+07	2.05E+07	1.84E+06	8.96	plasma	XCMS
Unkown	level4f318	608.4720	1.9	1.09E+07	9.68E+06	1.14E+07	1.30E+07	2.20E+07	1.34E+07	4.98E+06	37.15	plasma	LDA
Unkown	level4f318	608.4719	1.9	2.70E+06	2.41E+06	2.81E+06	3.31E+06	6.92E+06	3.63E+06	1.87E+06	51.53	plasma	CD
Unkown	level4f319	609.3894	3.0	3.54E+07	2.99E+07	2.69E+07	2.85E+07	2.54E+07	2.92E+07	3.84E+06	13.13	plasma	LDA
Unkown	level4f319	609.3894	2.7	5.53E+06	4.79E+06	4.12E+06	4.46E+06	4.00E+06	4.58E+06	6.14E+05	13.41	plasma	CD
Unkown	level4f319	609.3894	2.7	3.75E+06	4.17E+06	3.50E+06	3.83E+06	3.23E+06	3.70E+06	3.53E+05	9.54	plasma	XCMS
Unkown	level4f320	622.4508	1.9	5.76E+06	4.79E+06	5.35E+06	5.81E+06	6.13E+06	5.57E+06	5.17E+05	9.29	plasma	LDA
Unkown	level4f320	622.4519	1.9	1.69E+06	1.51E+06	1.52E+06	1.83E+06	1.57E+06	1.62E+06	1.36E+05	8.39	plasma	CD
Unkown	level4f321	634.8762	13.3	1.14E+08	1.09E+08	1.18E+08	1.31E+08	1.15E+08	1.17E+08	8.16E+06	6.95	plasma	LDA
Unkown	level4f321	634.8770	13.5	1.37E+07	1.70E+07	1.75E+07	3.25E+07	1.35E+07	1.88E+07	7.83E+06	41.55	plasma	CD
Unkown	level4f322	638.4484	2.0	3.07E+06	3.28E+06	2.51E+06	2.62E+06	3.27E+06	2.95E+06	3.65E+05	12.39	plasma	LDA
Unkown	level4f322	638.4477	2.0	7.34E+05	9.93E+05	8.36E+05	8.28E+05	1.04E+06	8.87E+05	1.28E+05	14.41	plasma	CD
Unkown	level4f323	646.4012	2.4	5.81E+06	4.21E+06	2.62E+06	2.43E+06	2.41E+06	3.50E+06	1.50E+06	42.76	plasma	LDA

Unkown	level4f323	646.4012	2.4	1.71E+06	1.20E+06	7.54E+05	7.63E+05	6.97E+05	1.02E+06	4.33E+05	42.23	plasma	CD
Unkown	level4f323	646.4009	2.4	1.35E+06	1.12E+06	6.55E+05	6.29E+05	5.52E+05	8.60E+05	3.51E+05	40.84	plasma	XCMS
Unkown	level4f324	648.3812	2.7	3.03E+07	3.04E+07	2.54E+07	3.30E+07	2.20E+07	2.82E+07	4.41E+06	15.62	plasma	LDA
Unkown	level4f324	648.3812	2.7	7.64E+06	6.51E+06	1.37E+07	6.89E+06	1.27E+07	9.49E+06	3.43E+06	36.18	plasma	CD
Unkown	level4f324	648.3819	2.7	8.37E+06	6.66E+06	6.49E+06	8.88E+06	6.41E+06	7.36E+06	1.17E+06	15.90	plasma	XCMS
Unkown	level4f325	648.3813	2.9	5.26E+07	5.21E+07	5.07E+07	4.49E+07	4.49E+07	4.90E+07	3.86E+06	7.86	plasma	LDA
Unkown	level4f325	648.3814	2.9	1.40E+07	1.35E+07	1.37E+07	1.35E+07	1.27E+07	1.35E+07	5.10E+05	3.78	plasma	CD
Unkown	level4f325	648.3817	2.9	1.47E+07	1.22E+07	1.19E+07	1.20E+07	1.12E+07	1.24E+07	1.33E+06	10.69	plasma	XCMS
Unkown	level4f326	648.3822	3.3	8.88E+07	7.19E+07	7.15E+07	6.82E+07	6.29E+07	7.26E+07	9.74E+06	13.40	plasma	LDA
Unkown	level4f326	648.3818	3.3	2.69E+07	2.24E+07	1.94E+07	2.29E+07	2.04E+07	2.24E+07	2.90E+06	12.95	plasma	CD
Unkown	level4f326	648.3816	3.3	2.04E+07	1.58E+07	1.66E+07	1.69E+07	1.49E+07	1.69E+07	2.09E+06	12.37	plasma	XCMS
Unkown	level4f327	649.3841	2.8	3.91E+06	5.16E+06	3.66E+06	5.22E+06	3.80E+06	4.35E+06	7.74E+05	17.81	plasma	LDA
Unkown	level4f327	649.3841	2.7	6.51E+05	6.06E+05	9.98E+05	1.05E+06	9.12E+05	8.43E+05	2.03E+05	24.03	plasma	CD
Unkown	level4f327	649.3842	2.9	1.74E+06	1.80E+06	1.55E+06	2.12E+06	1.31E+06	1.70E+06	3.01E+05	17.70	plasma	XCMS
Unkown	level4f328	649.3843	3.3	1.35E+07	9.95E+06	1.04E+07	1.10E+07	8.84E+06	1.07E+07	1.73E+06	16.07	plasma	LDA
Unkown	level4f328	649.3843	3.3	2.55E+06	2.00E+06	3.32E+06	2.25E+06	1.82E+06	2.39E+06	5.89E+05	24.65	plasma	CD
Unkown	level4f328	649.3841	3.3	3.45E+06	2.64E+06	2.74E+06	2.85E+06	2.27E+06	2.79E+06	4.26E+05	15.29	plasma	XCMS
Unkown	level4f329	652.4120	3.2	6.34E+07	5.07E+07	5.10E+07	5.23E+07	4.65E+07	5.28E+07	6.33E+06	12.00	plasma	LDA
Unkown	level4f329	652.4126	3.2	2.01E+07	1.54E+07	1.46E+07	1.55E+07	1.52E+07	1.62E+07	2.26E+06	13.96	plasma	CD
Unkown	level4f329	652.4125	3.2	1.53E+07	1.27E+07	1.30E+07	1.32E+07	1.17E+07	1.32E+07	1.31E+06	9.95	plasma	XCMS
Unkown	level4f330	652.4120	2.7	9.73E+07	8.30E+07	7.37E+07	7.53E+07	6.24E+07	7.83E+07	1.29E+07	16.44	plasma	LDA
Unkown	level4f330	652.4127	2.7	2.90E+07	2.51E+07	6.85E+06	1.82E+07	1.33E+07	1.85E+07	8.90E+06	48.08	plasma	CD
Unkown	level4f330	652.4130	2.7	1.70E+07	9.83E+06	1.29E+07	1.35E+07	1.22E+07	1.31E+07	2.59E+06	19.81	plasma	XCMS
Unkown	level4f331	652.5009	1.9	4.55E+06	3.91E+06	4.69E+06	6.27E+06	9.77E+06	5.84E+06	2.37E+06	40.53	plasma	LDA
Unkown	level4f331	652.5009	1.9	3.59E+05	7.75E+05	2.39E+05	2.06E+06	1.23E+06	9.32E+05	7.41E+05	79.52	plasma	CD
Unkown	level4f332	663.4933	1.7	6.55E+05	7.42E+05	8.73E+05	2.36E+06	4.68E+06	1.86E+06	1.72E+06	92.56	plasma	LDA
Unkown	level4f332	663.4930	1.7	1.40E+05	1.76E+05	4.77E+05	1.06E+06	1.68E+06	7.08E+05	6.59E+05	93.11	plasma	CD
Unkown	level4f332	663.4916	1.7	1.20E+05	1.10E+06	2.15E+05	3.31E+06	1.20E+06	1.19E+06	1.28E+06	108.21	plasma	XCMS
Unkown	level4f333	666.4783	1.9	3.77E+06	2.86E+06	3.05E+06	3.53E+06	4.09E+06	3.46E+06	5.06E+05	14.64	plasma	LDA
Unkown	level4f333	666.4784	1.9	1.10E+06	7.61E+05	8.26E+05	1.05E+06	1.34E+06	1.02E+06	2.32E+05	22.89	plasma	CD

Unkown	level4f334	670.4702	1.7	5.70E+06	5.57E+06	8.89E+06	1.63E+07	1.81E+07	1.09E+07	5.92E+06	54.25	plasma	LDA
Unkown	level4f334	670.4702	1.8	1.45E+06	1.28E+06	2.58E+06	4.72E+06	5.12E+06	3.03E+06	1.80E+06	59.49	plasma	CD
Unkown	level4f335	671.9924	1.7	2.06E+05	1.52E+05	3.62E+05	6.43E+05	1.56E+06	5.84E+05	5.78E+05	98.90	plasma	LDA
Unkown	level4f335	671.9924	1.8	6.66E+04	6.57E+04	1.40E+05	2.73E+05	5.86E+05	2.26E+05	2.18E+05	96.44	plasma	CD
Unkown	level4f335	671.9911	1.7	4.86E+04	3.05E+04	8.40E+04	1.71E+05	4.08E+05	1.49E+05	1.55E+05	104.27	plasma	XCMS
Unkown	level4f336	340.2092	2.6	1.92E+06	3.79E+06	2.89E+06	2.71E+06	3.02E+06	2.87E+06	6.70E+05	23.38	plasma	LDA
Unkown	level4f336	340.2113	2.7	1.21E+05	2.24E+05	5.47E+05	5.77E+05	4.54E+05	3.85E+05	2.02E+05	52.65	plasma	XCMS
Unkown	level4f337	692.4071	2.9	3.14E+07	3.37E+07	2.82E+07	2.03E+07	2.45E+07	2.76E+07	5.34E+06	19.33	plasma	LDA
Unkown	level4f337	692.4071	2.9	8.93E+06	9.33E+06	7.67E+06	9.90E+06	7.97E+06	8.76E+06	9.30E+05	10.62	plasma	CD
Unkown	level4f337	692.4076	2.9	8.96E+06	8.12E+06	6.70E+06	6.39E+06	5.23E+06	7.08E+06	1.47E+06	20.83	plasma	XCMS
Unkown	level4f338	692.4071	3.4	5.92E+07	4.76E+07	4.53E+07	4.74E+07	4.06E+07	4.80E+07	6.85E+06	14.27	plasma	LDA
Unkown	level4f338	692.4075	3.4	1.90E+07	1.62E+07	1.48E+07	1.60E+07	1.42E+07	1.60E+07	1.84E+06	11.46	plasma	CD
Unkown	level4f338	692.4075	3.4	1.47E+07	1.12E+07	1.10E+07	1.21E+07	1.02E+07	1.19E+07	1.73E+06	14.57	plasma	XCMS
Unkown	level4f339	692.4078	2.7	1.49E+07	1.69E+07	1.60E+07	1.78E+07	1.24E+07	1.56E+07	2.12E+06	13.59	plasma	LDA
Unkown	level4f339	692.4078	2.7	8.93E+06	4.24E+06	2.45E+06	2.57E+06	2.22E+06	4.08E+06	2.83E+06	69.30	plasma	CD
Unkown	level4f339	692.4073	2.7	4.14E+06	2.97E+06	4.14E+06	5.48E+06	3.41E+06	4.03E+06	9.53E+05	23.66	plasma	XCMS
Unkown	level4f340	693.4091	3.1	3.58E+06	3.54E+06	2.48E+06	3.14E+06	3.14E+06	3.17E+06	4.43E+05	13.96	plasma	LDA
Unkown	level4f340	693.4091	2.6	5.50E+05	5.49E+05	2.68E+05	5.12E+05	2.08E+05	4.17E+05	1.66E+05	39.75	plasma	CD
Unkown	level4f340	693.4101	3.4	2.58E+06	1.79E+06	1.89E+06	1.93E+06	1.61E+06	1.96E+06	3.68E+05	18.80	plasma	XCMS
Unkown	level4f341	348.7221	2.6	5.44E+06	5.17E+06	5.42E+06	3.94E+06	5.59E+06	5.11E+06	6.74E+05	13.19	plasma	LDA
Unkown	level4f341	348.7230	2.6	8.58E+06	2.89E+06	9.21E+06	3.48E+06	6.19E+06	6.07E+06	2.87E+06	47.28	plasma	CD
Unkown	level4f342	696.4362	3.2	2.91E+07	2.39E+07	2.26E+07	2.15E+07	1.83E+07	2.31E+07	3.98E+06	17.23	plasma	LDA
Unkown	level4f342	696.4381	3.2	9.03E+06	8.27E+06	9.17E+06	9.75E+06	9.17E+06	9.08E+06	5.27E+05	5.81	plasma	CD
Unkown	level4f342	696.4383	3.2	7.52E+06	6.42E+06	5.74E+06	5.60E+06	5.04E+06	6.06E+06	9.52E+05	15.71	plasma	XCMS
Unkown	level4f343	696.4387	2.8	4.04E+07	2.15E+07	2.16E+07	2.45E+07	1.62E+07	2.48E+07	9.19E+06	37.00	plasma	LDA
Unkown	level4f343	696.4387	2.8	8.96E+06	7.85E+06	9.27E+06	7.82E+06	6.71E+06	8.12E+06	1.02E+06	12.56	plasma	CD
Unkown	level4f343	696.4386	2.8	7.74E+06	5.30E+06	5.88E+06	6.20E+06	4.06E+06	5.84E+06	1.34E+06	22.97	plasma	XCMS
Unkown	level4f344	702.8648	13.4	3.77E+07	3.07E+07	4.52E+07	3.35E+07	3.04E+07	3.55E+07	6.16E+06	17.35	plasma	LDA
Unkown	level4f344	702.8642	13.4	4.50E+06	4.08E+06	4.53E+06	1.67E+06	3.99E+06	3.75E+06	1.19E+06	31.74	plasma	CD
Unkown	level4f345	710.5046	1.9	2.27E+06	2.05E+06	1.78E+06	2.33E+06	2.12E+06	2.11E+06	2.17E+05	10.28	plasma	LDA

Unkown	level4f345	710.5046	1.9	5.30E+05	5.29E+05	4.52E+05	7.41E+05	7.05E+05	5.91E+05	1.25E+05	21.13	plasma	CD
Unkown	level4f346	711.0709	10.7	2.10E+07	1.38E+07	1.41E+07	2.22E+07	1.65E+07	1.75E+07	3.89E+06	22.18	plasma	LDA
Unkown	level4f346	711.0713	10.6	1.09E+07	1.24E+07	9.01E+06	1.59E+07	1.63E+07	1.29E+07	3.18E+06	24.60	plasma	CD
Unkown	level4f346	711.0714	10.6	4.13E+06	3.07E+06	3.10E+06	4.27E+06	3.54E+06	3.62E+06	5.62E+05	15.52	plasma	XCMS
Unkown	level4f347	713.0679	10.7	2.93E+07	2.26E+07	2.78E+07	2.92E+07	2.74E+07	2.73E+07	2.75E+06	10.10	plasma	LDA
Unkown	level4f347	713.0679	10.8	4.65E+06	6.53E+06	6.25E+06	8.01E+06	7.41E+06	6.57E+06	1.28E+06	19.49	plasma	CD
Unkown	level4f347	713.0684	10.6	6.13E+06	3.79E+06	5.01E+06	5.57E+06	5.67E+06	5.23E+06	9.00E+05	17.20	plasma	XCMS
Unkown	level4f348	715.0647	10.7	1.91E+07	1.54E+07	1.48E+07	1.71E+07	1.68E+07	1.66E+07	1.69E+06	10.18	plasma	LDA
Unkown	level4f348	715.0647	10.6	6.40E+06	3.62E+06	4.35E+06	5.52E+06	5.83E+06	5.14E+06	1.13E+06	22.03	plasma	CD
Unkown	level4f348	715.0652	10.7	3.07E+06	2.52E+06	2.42E+06	2.96E+06	2.90E+06	2.77E+06	2.87E+05	10.36	plasma	XCMS
Unkown	level4f349	360.2061	2.7	2.38E+06	7.25E+06	4.73E+06	3.42E+06	2.26E+06	4.01E+06	2.07E+06	51.54	plasma	LDA
Unkown	level4f349	360.2077	2.7	1.84E+06	1.08E+06	1.45E+06	1.28E+06	8.69E+05	1.31E+06	3.71E+05	28.42	plasma	CD
Unkown	level4f349	360.2071	2.7	6.11E+05	1.42E+06	5.74E+05	8.80E+05	4.99E+05	7.96E+05	3.75E+05	47.17	plasma	XCMS
Unkown	level4f350	360.2069	3.3	2.86E+06	3.10E+06	4.34E+06	2.23E+06	3.97E+06	3.30E+06	8.54E+05	25.87	plasma	LDA
Unkown	level4f350	360.2070	3.4	6.88E+05	1.06E+06	1.19E+06	7.97E+05	8.01E+05	9.08E+05	2.10E+05	23.12	plasma	XCMS
Unkown	level4f351	732.5526	2.6	1.91E+06	2.08E+06	1.88E+06	1.47E+06	1.50E+06	1.77E+06	2.72E+05	15.35	plasma	LDA
Unkown	level4f351	732.5526	2.6	7.46E+05	8.84E+05	8.23E+05	6.24E+05	5.03E+05	7.16E+05	1.54E+05	21.49	plasma	CD
Unkown	level4f351	732.5543	2.6	5.86E+05	5.46E+05	7.02E+05	4.80E+05	3.92E+05	5.41E+05	1.16E+05	21.47	plasma	XCMS
Unkown	level4f352	736.4333	3.4	2.67E+07	2.11E+07	1.92E+07	2.07E+07	1.68E+07	2.09E+07	3.66E+06	17.51	plasma	LDA
Unkown	level4f352	736.4338	3.4	2.48E+07	2.03E+07	2.36E+07	1.81E+07	1.63E+07	2.06E+07	3.58E+06	17.37	plasma	CD
Unkown	level4f352	736.4334	3.4	6.82E+06	5.80E+06	5.03E+06	5.49E+06	4.44E+06	5.51E+06	8.90E+05	16.14	plasma	XCMS
Unkown	level4f353	736.4356	2.9	1.32E+07	1.16E+07	1.05E+07	9.47E+06	1.05E+07	1.11E+07	1.41E+06	12.77	plasma	LDA
Unkown	level4f353	736.4343	2.9	2.86E+06	5.14E+06	2.48E+06	3.83E+06	3.87E+06	3.63E+06	1.03E+06	28.46	plasma	CD
Unkown	level4f353	736.4335	3.0	4.23E+06	3.26E+06	2.73E+06	2.72E+06	3.25E+06	3.24E+06	6.15E+05	18.98	plasma	XCMS
Unkown	level4f354	740.4635	2.8	1.17E+07	7.09E+06	5.49E+06	5.85E+06	7.41E+06	7.51E+06	2.47E+06	32.95	plasma	LDA
Unkown	level4f354	740.4653	2.8	1.92E+07	1.82E+07	1.58E+07	1.60E+07	1.52E+07	1.69E+07	1.74E+06	10.34	plasma	CD
Unkown	level4f354	740.4640	2.8	1.85E+06	1.77E+06	1.57E+06	1.59E+06	1.24E+06	1.60E+06	2.36E+05	14.70	plasma	XCMS
Unkown	level4f355	740.4639	3.3	6.49E+06	5.82E+06	5.66E+06	4.78E+06	4.34E+06	5.42E+06	8.57E+05	15.81	plasma	LDA
Unkown	level4f355	740.4654	3.3	2.62E+07	2.40E+07	2.68E+07	2.35E+07	2.26E+07	2.46E+07	1.81E+06	7.36	plasma	CD
Unkown	level4f355	740.4641	3.3	1.80E+06	1.48E+06	1.53E+06	1.24E+06	1.21E+06	1.45E+06	2.40E+05	16.54	plasma	XCMS

Unkown	level4f356	377.2340	3.0	2.54E+07	2.67E+07	1.71E+07	1.88E+07	1.75E+07	2.11E+07	4.56E+06	21.60	plasma	LDA
Unkown	level4f356	377.2340	2.9	5.68E+06	5.89E+06	3.18E+06	4.74E+06	5.53E+06	5.00E+06	1.11E+06	22.11	plasma	XCMS
Unkown	level4f356	377.2340	3.0	6.18E+06	3.82E+06	3.33E+06	3.43E+06	2.80E+06	3.91E+06	1.32E+06	33.74	plasma	CD
Unkown	level4f357	756.5541	2.6	6.06E+06	4.13E+06	5.71E+06	3.39E+06	4.06E+06	4.67E+06	1.15E+06	24.70	plasma	LDA
Unkown	level4f357	756.5540	2.6	1.12E+06	1.47E+06	1.40E+06	1.69E+06	2.07E+06	1.55E+06	3.56E+05	22.92	plasma	CD
Unkown	level4f358	379.2495	2.8	6.08E+07	3.90E+07	3.81E+07	4.06E+07	1.33E+07	3.84E+07	1.69E+07	43.98	plasma	LDA
Unkown	level4f358	379.2496	2.8	1.10E+07	5.18E+06	7.27E+06	8.01E+06	6.60E+06	7.61E+06	2.16E+06	28.35	plasma	XCMS
Unkown	level4f358	379.2495	2.6	1.12E+06	1.47E+06	1.40E+06	1.69E+06	2.07E+06	1.55E+06	3.56E+05	22.92	plasma	CD
Unkown	level4f359	758.5721	2.6	1.08E+08	1.05E+08	9.24E+07	8.83E+07	9.14E+07	9.70E+07	8.85E+06	9.13	plasma	LDA
Unkown	level4f359	758.5714	2.6	4.42E+07	6.29E+07	3.90E+07	5.50E+07	4.44E+07	4.91E+07	9.67E+06	19.69	plasma	CD
Unkown	level4f359	758.5706	2.6	3.08E+07	3.44E+07	3.31E+07	3.02E+07	2.56E+07	3.08E+07	3.36E+06	10.89	plasma	XCMS
Unkown	level4f361	382.2206	3.2	3.32E+06	3.28E+06	3.39E+06	6.01E+06	1.45E+06	3.49E+06	1.63E+06	46.71	plasma	LDA
Unkown	level4f361	382.2199	3.4	9.48E+05	1.10E+06	9.33E+05	8.79E+05	7.08E+05	9.13E+05	1.40E+05	15.32	plasma	XCMS
Unkown	level4f361	382.2203	3.4	4.21E+05	1.36E+06	1.15E+06	9.98E+05	3.90E+05	8.64E+05	4.39E+05	50.78	plasma	CD
Unkown	level4f363	780.4589	3.0	5.79E+06	5.71E+06	2.22E+06	5.24E+06	3.51E+06	4.50E+06	1.57E+06	34.91	plasma	LDA
Unkown	level4f363	780.4601	3.0	7.99E+06	8.13E+06	5.70E+06	8.48E+06	5.84E+06	7.23E+06	1.34E+06	18.59	plasma	CD
Unkown	level4f364	390.7336	2.7	1.44E+07	1.66E+07	1.40E+07	1.29E+07	1.22E+07	1.40E+07	1.68E+06	11.95	plasma	LDA
Unkown	level4f364	390.7336	2.7	4.43E+06	5.46E+06	3.80E+06	4.36E+06	2.67E+06	4.15E+06	1.02E+06	24.58	plasma	CD
Unkown	level4f364	390.7340	2.7	3.81E+06	5.32E+06	3.56E+06	3.48E+06	3.12E+06	3.86E+06	8.51E+05	22.06	plasma	XCMS
Unkown	level4f365	390.7336	3.0	2.49E+07	2.49E+07	2.14E+07	2.47E+07	1.90E+07	2.30E+07	2.69E+06	11.68	plasma	LDA
Unkown	level4f365	390.7339	3.0	6.28E+06	7.04E+06	5.02E+06	6.99E+06	5.34E+06	6.14E+06	9.28E+05	15.12	plasma	XCMS
Unkown	level4f365	390.7336	3.0	7.99E+06	8.13E+06	5.70E+06	8.48E+06	5.84E+06	7.23E+06	1.34E+06	18.59	plasma	CD
Unkown	level4f366	780.5547	2.6	3.28E+06	6.32E+06	6.99E+06	1.04E+07	9.39E+06	7.27E+06	2.78E+06	38.25	plasma	LDA
Unkown	level4f366	780.5547	2.6	1.27E+06	2.36E+06	2.58E+06	2.76E+06	3.11E+06	2.42E+06	6.98E+05	28.91	plasma	CD
Unkown	level4f366	780.5535	2.6	9.54E+05	1.12E+06	1.07E+06	1.75E+06	7.93E+05	1.14E+06	3.64E+05	32.03	plasma	XCMS
Unkown	level4f367	782.5706	4.5	5.41E+07	3.09E+07	3.96E+07	3.57E+07	3.90E+07	3.99E+07	8.66E+06	21.73	plasma	LDA
Unkown	level4f367	782.5706	4.5	1.83E+07	9.38E+06	8.13E+06	1.09E+07	8.45E+06	1.10E+07	4.20E+06	38.10	plasma	CD
Unkown	level4f368	782.5706	2.6	6.17E+07	1.18E+08	1.01E+08	1.42E+08	1.29E+08	1.10E+08	3.11E+07	28.21	plasma	LDA
Unkown	level4f368	782.5709	2.6	2.82E+07	5.33E+07	4.28E+07	6.15E+07	2.56E+07	4.23E+07	1.56E+07	36.79	plasma	CD
Unkown	level4f368	782.5706	2.6	1.73E+07	2.11E+07	2.15E+07	2.24E+07	2.05E+07	2.06E+07	1.93E+06	9.39	plasma	XCMS

Unkown	level4f369	392.7492	3.3	1.52E+07	1.46E+07	1.39E+07	1.47E+06	1.23E+07	1.15E+07	5.69E+06	49.58	plasma	LDA
Unkown	level4f369	392.7494	3.3	3.85E+06	3.39E+06	3.56E+06	3.89E+06	3.16E+06	3.57E+06	3.11E+05	8.70	plasma	XCMS
Unkown	level4f369	392.7492	3.3	4.56E+06	5.17E+06	3.90E+06	4.65E+06	3.35E+06	4.33E+06	7.09E+05	16.39	plasma	CD
Unkown	level4f370	784.5867	2.8	1.48E+07	1.72E+07	1.63E+07	1.79E+07	1.55E+07	1.63E+07	1.27E+06	7.76	plasma	LDA
Unkown	level4f370	784.5875	2.6	1.21E+07	2.39E+07	1.44E+07	2.73E+07	2.24E+07	2.00E+07	6.50E+06	32.48	plasma	CD
Unkown	level4f370	784.5853	2.6	4.01E+06	4.11E+06	4.40E+06	4.37E+06	3.54E+06	4.09E+06	3.48E+05	8.50	plasma	XCMS
Unkown	level4f371	80.9480	11.5	8.84E+07	1.14E+08	1.13E+08	9.95E+07	1.04E+08	1.04E+08	1.05E+07	10.16	plasma	LDA
Unkown	level4f371	80.9479	11.5	3.26E+07	4.46E+07	3.90E+07	3.63E+07	3.97E+07	3.84E+07	4.40E+06	11.46	plasma	CD
Unkown	level4f371	80.9480	11.5	2.02E+07	2.82E+07	2.43E+07	2.32E+07	2.34E+07	2.39E+07	2.88E+06	12.05	plasma	XCMS
Unkown	level4f372	399.2470	3.4	1.28E+08	1.12E+08	1.20E+08	1.10E+08	8.71E+07	1.11E+08	1.54E+07	13.85	plasma	LDA
Unkown	level4f372	399.2470	3.4	4.75E+07	4.04E+07	4.36E+07	4.49E+07	3.62E+07	4.25E+07	4.38E+06	10.30	plasma	CD
Unkown	level4f372	399.2470	3.4	2.99E+07	2.91E+07	3.93E+07	2.65E+07	2.19E+07	2.94E+07	6.39E+06	21.76	plasma	XCMS
Unkown	level4f373	399.2470	2.6	4.75E+07	3.49E+07	3.15E+07	4.13E+07	2.74E+07	3.65E+07	7.96E+06	21.79	plasma	LDA
Unkown	level4f373	399.2473	3.0	2.15E+07	1.96E+07	1.69E+07	2.04E+07	2.14E+07	1.99E+07	1.87E+06	9.38	plasma	XCMS
Unkown	level4f373	399.2470	2.3	1.32E+07	1.01E+07	7.70E+06	9.55E+06	4.70E+06	9.04E+06	3.12E+06	34.57	plasma	CD
Unkown	level4f374	399.7484	2.7	1.07E+07	7.55E+06	5.95E+06	5.60E+06	6.56E+06	7.27E+06	2.04E+06	28.07	plasma	LDA
Unkown	level4f374	399.7487	3.0	5.98E+06	5.39E+06	3.94E+06	5.00E+06	5.20E+06	5.10E+06	7.48E+05	14.67	plasma	XCMS
Unkown	level4f374	399.7484	2.7	2.17E+06	8.76E+05	1.66E+06	8.58E+05	2.76E+05	1.17E+06	7.47E+05	63.89	plasma	CD
Unkown	level4f375	399.7487	3.0	2.57E+07	2.02E+07	1.46E+07	2.04E+07	2.04E+07	2.02E+07	3.92E+06	19.34	plasma	LDA
Unkown	level4f375	399.7487	3.0	5.98E+06	5.39E+06	3.94E+06	5.00E+06	5.20E+06	5.10E+06	7.48E+05	14.67	plasma	XCMS
Unkown	level4f375	399.7487	3.0	7.81E+06	6.58E+06	2.91E+06	5.42E+06	5.54E+06	5.65E+06	1.81E+06	32.06	plasma	CD
Unkown	level4f376	401.2628	2.8	5.87E+07	4.28E+07	4.17E+07	5.01E+07	3.97E+07	4.66E+07	7.83E+06	16.81	plasma	LDA
Unkown	level4f376	401.2628	2.8	1.44E+07	1.11E+07	1.04E+07	1.21E+07	1.34E+07	1.23E+07	1.64E+06	13.32	plasma	XCMS
Unkown	level4f376	401.2628	2.8	1.57E+07	1.43E+07	1.09E+07	1.42E+07	1.39E+07	1.38E+07	1.75E+06	12.66	plasma	CD
Unkown	level4f377	401.2628	3.3	7.40E+07	6.32E+07	6.50E+07	6.84E+07	5.49E+07	6.51E+07	7.00E+06	10.76	plasma	LDA
Unkown	level4f377	401.2626	3.3	1.77E+07	1.49E+07	1.74E+07	1.68E+07	1.34E+07	1.60E+07	1.84E+06	11.45	plasma	XCMS
Unkown	level4f377	401.2628	3.3	2.54E+07	2.39E+07	2.22E+07	2.11E+07	1.88E+07	2.23E+07	2.52E+06	11.31	plasma	CD
Unkown	level4f378	806.5705	2.6	3.43E+07	2.90E+07	2.72E+07	4.13E+07	3.84E+07	3.41E+07	5.99E+06	17.59	plasma	LDA
Unkown	level4f378	806.5705	2.8	5.94E+06	1.33E+07	8.18E+06	1.90E+07	1.71E+07	1.27E+07	5.59E+06	44.02	plasma	CD
Unkown	level4f378	806.5701	2.6	4.43E+06	6.06E+06	6.09E+06	7.58E+06	7.18E+06	6.27E+06	1.22E+06	19.55	plasma	XCMS

Unkown	level4f379	806.5710	4.3	8.24E+06	8.28E+06	5.84E+06	1.09E+07	6.49E+06	7.94E+06	1.95E+06	24.61	plasma	LDA
Unkown	level4f379	806.5710	4.2	3.04E+06	2.11E+06	2.59E+06	3.45E+06	2.85E+06	2.81E+06	5.02E+05	17.89	plasma	CD
Unkown	level4f380	404.2327	2.7	2.24E+06	3.99E+06	2.96E+06	3.62E+06	3.51E+06	3.26E+06	6.80E+05	20.86	plasma	LDA
Unkown	level4f380	404.2326	2.6	4.18E+05	8.39E+05	5.67E+05	5.84E+05	7.93E+05	6.40E+05	1.74E+05	27.12	plasma	XCMS
Unkown	level4f380	404.2327	2.7	4.64E+05	1.21E+06	5.94E+05	6.77E+05	1.12E+06	8.12E+05	3.30E+05	40.65	plasma	CD
Unkown	level4f381	808.5855	2.8	9.88E+06	7.70E+06	7.37E+06	1.13E+07	9.22E+06	9.09E+06	1.61E+06	17.69	plasma	LDA
Unkown	level4f381	808.5855	2.6	2.17E+06	3.82E+06	3.90E+06	5.18E+06	5.37E+06	4.09E+06	1.29E+06	31.45	plasma	CD
Unkown	level4f381	808.5855	2.6	1.55E+06	1.83E+06	1.77E+06	2.33E+06	2.16E+06	1.93E+06	3.15E+05	16.31	plasma	XCMS
Unkown	level4f382	810.6013	2.7	5.51E+06	5.95E+06	5.36E+06	5.74E+06	4.19E+06	5.35E+06	6.88E+05	12.86	plasma	LDA
Unkown	level4f382	810.6010	2.6	2.90E+06	3.19E+06	2.07E+06	3.38E+06	2.21E+06	2.75E+06	5.86E+05	21.33	plasma	CD
Unkown	level4f382	810.6008	2.6	1.52E+06	1.58E+06	1.62E+06	1.38E+06	1.38E+06	1.49E+06	1.10E+05	7.35	plasma	XCMS
Unkown	level4f384	412.7469	3.0	1.66E+07	1.70E+07	1.27E+07	1.58E+07	1.27E+07	1.50E+07	2.12E+06	14.16	plasma	LDA
Unkown	level4f384	412.7468	3.5	7.41E+06	7.05E+06	6.89E+06	6.83E+06	5.85E+06	6.81E+06	5.78E+05	8.50	plasma	XCMS
Unkown	level4f384	412.7469	3.0	5.69E+06	3.54E+06	4.65E+06	5.00E+06	3.90E+06	4.56E+06	8.60E+05	18.87	plasma	CD
Unkown	level4f385	412.7468	3.5	3.02E+07	2.58E+07	2.64E+07	2.64E+07	2.13E+07	2.60E+07	3.15E+06	12.11	plasma	LDA
Unkown	level4f385	412.7468	3.5	7.41E+06	7.05E+06	6.89E+06	6.83E+06	5.85E+06	6.81E+06	5.78E+05	8.50	plasma	XCMS
Unkown	level4f385	412.7468	3.5	9.79E+06	1.13E+07	8.04E+06	9.58E+06	7.21E+06	9.19E+06	1.60E+06	17.46	plasma	CD
Unkown	level4f386	414.7618	2.6	3.71E+06	5.58E+06	4.00E+06	3.66E+06	4.16E+06	4.22E+06	7.87E+05	18.64	plasma	LDA
Unkown	level4f386	414.7620	2.8	4.29E+05	6.05E+05	5.02E+05	4.09E+05	7.28E+05	5.34E+05	1.33E+05	24.82	plasma	CD
Unkown	level4f386	414.7622	2.6	1.20E+06	1.33E+06	9.76E+05	1.01E+06	1.01E+06	1.10E+06	1.53E+05	13.86	plasma	XCMS
Unkown	level4f387	414.7620	3.3	4.23E+06	5.22E+06	5.29E+06	4.84E+06	3.93E+06	4.70E+06	6.00E+05	12.75	plasma	LDA
Unkown	level4f387	414.7621	3.3	1.09E+06	1.16E+06	1.29E+06	1.44E+06	1.16E+06	1.23E+06	1.38E+05	11.24	plasma	XCMS
Unkown	level4f387	414.7621	2.8	9.75E+05	1.19E+06	9.43E+05	9.51E+05	8.31E+05	9.77E+05	1.30E+05	13.31	plasma	XCMS
Unkown	level4f387	414.7620	3.4	1.18E+06	1.32E+06	1.25E+06	1.54E+06	1.30E+06	1.32E+06	1.33E+05	10.11	plasma	CD
Unkown	level4f388	84.0445	20.4	5.72E+06	7.66E+06	6.85E+06	7.66E+06	6.25E+06	6.83E+06	8.57E+05	12.55	plasma	LDA
Unkown	level4f388	84.0445	20.4	1.66E+06	1.20E+06	1.57E+06	1.20E+06	1.28E+06	1.38E+06	2.16E+05	15.67	plasma	CD
Unkown	level4f388	84.0444	20.3	1.33E+06	1.64E+06	5.48E+05	1.70E+06	8.99E+05	1.22E+06	4.94E+05	40.33	plasma	XCMS
Unkown	level4f389	421.2603	2.7	5.86E+07	5.21E+07	5.43E+07	6.67E+07	4.48E+07	5.53E+07	8.10E+06	14.63	plasma	LDA
Unkown	level4f389	421.2603	2.7	1.65E+07	1.58E+07	1.58E+07	1.60E+07	1.15E+07	1.51E+07	2.05E+06	13.54	plasma	CD
Unkown	level4f390	421.2603	3.0	1.38E+08	1.09E+08	9.89E+07	1.30E+08	1.17E+08	1.19E+08	1.58E+07	13.31	plasma	LDA

Unkown	level4f390	421.2604	3.0	3.82E+07	1.11E+05	2.64E+07	2.87E+07	2.73E+07	2.42E+07	1.42E+07	58.94	plasma	XCMS
Unkown	level4f390	421.2603	3.0	5.63E+07	4.35E+07	3.32E+07	3.80E+07	3.17E+07	4.05E+07	9.94E+06	24.53	plasma	CD
Unkown	level4f391	421.2603	3.5	2.00E+08	1.76E+08	1.64E+08	1.77E+08	1.42E+08	1.72E+08	2.13E+07	12.39	plasma	LDA
Unkown	level4f391	421.2603	3.5	4.20E+07	4.14E+07	4.16E+07	3.99E+07	3.87E+07	4.07E+07	1.38E+06	3.39	plasma	XCMS
Unkown	level4f391	421.2603	3.5	6.95E+07	6.65E+07	6.93E+07	6.67E+07	5.60E+07	6.56E+07	5.53E+06	8.43	plasma	CD
Unkown	level4f393	423.2764	2.8	4.28E+07	3.59E+07	3.22E+07	3.93E+07	3.46E+07	3.70E+07	4.13E+06	11.18	plasma	LDA
Unkown	level4f393	423.2764	2.8	1.22E+07	1.07E+07	9.67E+06	1.25E+07	1.04E+07	1.11E+07	1.23E+06	11.07	plasma	CD
Unkown	level4f393	423.2761	2.8	1.11E+07	9.27E+06	8.16E+06	1.30E+07	8.77E+06	1.01E+07	1.98E+06	19.65	plasma	XCMS
Unkown	level4f394	423.2757	3.3	6.25E+07	5.28E+07	5.39E+07	5.59E+07	4.49E+07	5.40E+07	6.33E+06	11.72	plasma	LDA
Unkown	level4f394	423.2760	3.3	1.51E+07	1.24E+07	1.33E+07	1.38E+07	1.12E+07	1.31E+07	1.48E+06	11.27	plasma	XCMS
Unkown	level4f394	423.2757	3.3	2.02E+07	1.55E+07	1.85E+07	1.96E+07	1.65E+07	1.80E+07	2.00E+06	11.09	plasma	CD
Unkown	level4f395	423.2764	2.7	1.79E+07	1.20E+07	7.61E+06	1.03E+07	7.50E+06	1.11E+07	4.29E+06	38.75	plasma	LDA
Unkown	level4f395	423.2764	2.7	3.40E+06	2.23E+06	1.29E+06	1.60E+06	1.74E+06	2.05E+06	8.28E+05	40.35	plasma	CD
Unkown	level4f398	430.2649	4.9	1.38E+07	1.34E+07	4.15E+06	1.12E+07	1.07E+07	1.07E+07	3.88E+06	36.39	plasma	LDA
Unkown	level4f398	430.2648	4.9	3.05E+06	1.14E+06	1.45E+06	1.03E+06	7.73E+05	1.49E+06	9.05E+05	60.80	plasma	XCMS
Unkown	level4f399	434.7597	2.6	4.96E+06	8.22E+06	6.91E+06	7.33E+06	6.47E+06	6.78E+06	1.20E+06	17.74	plasma	LDA
Unkown	level4f399	434.7603	2.7	1.46E+05	1.69E+06	1.37E+06	1.69E+06	1.42E+06	1.26E+06	6.43E+05	50.83	plasma	XCMS
Unkown	level4f399	434.7597	2.6	1.58E+06	2.95E+06	2.32E+06	2.27E+06	1.92E+06	2.21E+06	5.10E+05	23.11	plasma	CD
Unkown	level4f400	434.7605	3.5	1.21E+07	1.63E+07	1.37E+07	1.24E+07	1.02E+07	1.29E+07	2.27E+06	17.53	plasma	LDA
Unkown	level4f400	434.7605	3.5	3.96E+06	5.29E+06	4.45E+06	3.74E+06	3.38E+06	4.16E+06	7.40E+05	17.76	plasma	CD
Unkown	level4f400	434.7599	3.5	3.05E+06	4.09E+06	3.82E+06	4.97E+06	2.71E+06	3.73E+06	8.92E+05	23.93	plasma	XCMS
Unkown	level4f401	434.7605	3.0	8.01E+06	9.17E+06	6.43E+06	7.65E+06	6.48E+06	7.55E+06	1.15E+06	15.19	plasma	LDA
Unkown	level4f401	434.7603	2.7	1.46E+05	1.69E+06	1.37E+06	1.69E+06	1.42E+06	1.26E+06	6.43E+05	50.83	plasma	XCMS
Unkown	level4f401	434.7605	3.0	1.68E+06	2.95E+06	1.16E+06	2.65E+06	1.92E+06	2.07E+06	7.25E+05	35.02	plasma	CD
Unkown	level4f402	89.0687	17.8	1.17E+07	1.26E+07	1.33E+07	9.56E+06	8.87E+06	1.12E+07	1.92E+06	17.12	plasma	LDA
Unkown	level4f402	89.0687	17.7	3.71E+06	3.19E+06	2.89E+06	2.88E+06	1.21E+06	2.77E+06	9.38E+05	33.81	plasma	CD
Unkown	level4f402	89.0687	17.8	3.46E+06	1.43E+06	2.91E+06	1.25E+06	8.25E+05	1.98E+06	1.14E+06	57.77	plasma	XCMS
Unkown	level4f403	443.2739	3.5	1.98E+08	1.87E+08	1.64E+08	1.72E+08	1.40E+08	1.72E+08	2.25E+07	13.03	plasma	LDA
Unkown	level4f403	443.2735	3.5	4.33E+07	4.35E+07	4.11E+07	4.07E+07	3.27E+07	4.03E+07	4.41E+06	10.96	plasma	XCMS
Unkown	level4f403	443.2739	3.5	7.08E+07	7.15E+07	6.75E+07	6.83E+07	5.07E+07	6.58E+07	8.60E+06	13.07	plasma	CD

Unkown	level4f404	443.2734	3.0	1.39E+08	1.15E+08	9.68E+07	1.30E+08	1.09E+08	1.18E+08	1.67E+07	14.11	plasma	LDA
Unkown	level4f404	443.2735	3.0	3.16E+07	2.65E+07	2.41E+07	2.86E+07	2.62E+07	2.74E+07	2.84E+06	10.37	plasma	XCMS
Unkown	level4f404	443.2734	3.0	4.53E+07	3.70E+07	2.81E+07	3.04E+07	2.98E+07	3.41E+07	7.09E+06	20.77	plasma	CD
Unkown	level4f405	443.2744	2.7	5.65E+07	5.15E+07	5.51E+07	5.55E+07	3.98E+07	5.17E+07	6.88E+06	13.32	plasma	LDA
Unkown	level4f405	443.2738	2.7	1.44E+07	1.40E+07	1.12E+07	1.36E+07	9.87E+06	1.26E+07	1.97E+06	15.61	plasma	XCMS
Unkown	level4f405	443.2744	2.7	1.22E+07	1.15E+07	1.37E+07	1.60E+07	2.98E+07	1.66E+07	7.57E+06	45.51	plasma	CD
Unkown	level4f407	445.2886	3.4	3.88E+07	3.68E+07	3.34E+07	3.52E+07	2.97E+07	3.48E+07	3.46E+06	9.96	plasma	LDA
Unkown	level4f407	445.2890	3.4	1.00E+07	8.60E+06	8.73E+06	8.93E+06	7.54E+06	8.76E+06	8.85E+05	10.10	plasma	XCMS
Unkown	level4f407	445.2886	3.4	1.15E+07	1.09E+07	1.26E+07	1.29E+07	9.93E+06	1.15E+07	1.21E+06	10.52	plasma	CD
Unkown	level4f408	445.2892	2.9	3.38E+07	3.07E+07	2.37E+07	2.98E+07	2.56E+07	2.87E+07	4.06E+06	14.12	plasma	LDA
Unkown	level4f408	445.2892	2.9	6.50E+06	6.11E+06	4.84E+06	5.94E+06	5.36E+06	5.75E+06	6.54E+05	11.37	plasma	XCMS
Unkown	level4f408	445.2892	2.9	2.11E+06	5.62E+06	5.40E+06	8.70E+06	6.66E+06	5.70E+06	2.39E+06	41.98	plasma	CD
Unkown	level4f409	90.9766	13.6	4.24E+08	7.06E+08	6.15E+08	7.03E+08	6.20E+08	6.14E+08	1.14E+08	18.65	plasma	LDA
Unkown	level4f409	90.9766	13.5	8.50E+07	1.09E+08	5.86E+07	7.19E+07	6.28E+07	7.75E+07	2.04E+07	26.31	plasma	CD
Unkown	level4f409	90.9765	13.6	1.54E+06	3.94E+07	6.43E+05	8.73E+05	6.26E+07	2.10E+07	2.86E+07	136.02	plasma	XCMS
Unkown	level4f410	91.0542	14.6	1.22E+07	1.06E+07	1.14E+07	1.10E+07	1.13E+07	1.13E+07	5.79E+05	5.12	plasma	LDA
Unkown	level4f410	91.0543	14.6	3.04E+06	2.30E+06	2.15E+06	2.58E+06	2.18E+06	2.45E+06	3.71E+05	15.13	plasma	CD
Unkown	level4f410	91.0544	14.7	2.91E+06	2.55E+06	2.71E+06	2.61E+06	1.07E+06	2.37E+06	7.40E+05	31.20	plasma	XCMS
Unkown	level4f411	452.2787	5.0	3.85E+07	4.30E+07	1.29E+07	3.53E+07	3.02E+07	3.20E+07	1.16E+07	36.42	plasma	LDA
Unkown	level4f411	452.2785	5.1	3.59E+06	1.02E+07	4.03E+06	2.67E+06	3.84E+06	4.88E+06	3.04E+06	62.42	plasma	XCMS
Unkown	level4f411	452.2787	5.0	1.21E+07	1.16E+07	7.45E+06	5.33E+06	5.58E+06	8.41E+06	3.25E+06	38.60	plasma	CD
Unkown	level4f412	456.7728	2.7	2.40E+06	3.30E+06	3.35E+06	2.68E+06	2.61E+06	2.87E+06	4.30E+05	15.01	plasma	LDA
Unkown	level4f412	456.7728	2.7	4.71E+05	1.54E+06	4.26E+05	1.33E+06	7.74E+05	9.10E+05	5.06E+05	55.65	plasma	CD
Unkown	level4f413	456.7729	3.6	4.93E+06	6.86E+06	4.04E+06	5.42E+06	4.49E+06	5.15E+06	1.08E+06	21.06	plasma	LDA
Unkown	level4f413	456.7728	3.6	1.38E+06	1.67E+06	1.05E+06	1.41E+06	1.11E+06	1.32E+06	2.48E+05	18.71	plasma	XCMS
Unkown	level4f413	456.7729	3.6	1.84E+06	2.56E+06	1.41E+06	1.69E+06	1.29E+06	1.76E+06	4.98E+05	28.37	plasma	CD
Unkown	level4f414	459.2812	2.6	6.44E+06	9.89E+06	6.52E+06	6.81E+06	6.28E+06	7.19E+06	1.52E+06	21.18	plasma	LDA
Unkown	level4f414	459.2792	2.6	1.78E+06	2.15E+06	1.97E+06	1.41E+06	1.71E+06	1.81E+06	2.81E+05	15.57	plasma	CD
Unkown	level4f414	459.2804	2.6	1.02E+06	1.87E+06	1.61E+06	1.33E+06	1.42E+06	1.45E+06	3.18E+05	21.88	plasma	XCMS
Unkown	level4f415	465.2863	2.7	4.36E+07	3.82E+07	3.27E+07	4.89E+07	3.15E+07	3.90E+07	7.34E+06	18.84	plasma	LDA

Unkown	level4f415	465.2867	2.7	1.13E+07	9.36E+06	7.88E+06	1.01E+07	9.00E+06	9.53E+06	1.28E+06	13.38	plasma	XCMS
Unkown	level4f415	465.2863	2.7	4.84E+06	1.75E+07	1.09E+07	1.19E+07	8.19E+06	1.07E+07	4.71E+06	44.16	plasma	CD
Unkown	level4f416	465.2866	3.1	1.19E+08	9.94E+07	8.63E+07	9.66E+07	8.16E+07	9.66E+07	1.45E+07	14.97	plasma	LDA
Unkown	level4f416	465.2866	3.1	3.30E+07	2.41E+07	2.20E+07	2.29E+07	2.05E+07	2.45E+07	4.92E+06	20.11	plasma	XCMS
Unkown	level4f416	465.2866	3.1	3.98E+07	2.50E+07	3.39E+07	3.44E+07	2.86E+07	3.24E+07	5.70E+06	17.60	plasma	CD
Unkown	level4f417	465.2865	3.5	1.71E+08	1.53E+08	1.49E+08	1.39E+08	1.15E+08	1.45E+08	2.06E+07	14.17	plasma	LDA
Unkown	level4f417	465.2865	3.5	4.15E+07	4.10E+07	5.31E+07	3.29E+07	2.74E+07	3.92E+07	9.76E+06	24.90	plasma	XCMS
Unkown	level4f417	465.2865	3.5	6.02E+07	5.98E+07	6.04E+07	5.71E+07	4.69E+07	5.69E+07	5.74E+06	10.09	plasma	CD
Unkown	level4f418	467.3016	2.6	3.16E+06	3.36E+06	2.38E+06	3.22E+06	2.65E+06	2.96E+06	4.17E+05	14.10	plasma	LDA
Unkown	level4f418	467.3016	2.6	3.93E+05	1.21E+06	7.40E+05	8.11E+05	5.93E+05	7.49E+05	3.01E+05	40.25	plasma	CD
Unkown	level4f419	467.3018	3.4	1.89E+07	1.90E+07	1.95E+07	1.89E+07	1.37E+07	1.80E+07	2.42E+06	13.48	plasma	LDA
Unkown	level4f419	467.3019	3.4	4.92E+06	5.03E+06	5.03E+06	4.83E+06	3.40E+06	4.64E+06	6.99E+05	15.06	plasma	XCMS
Unkown	level4f419	467.3018	3.4	6.34E+06	6.19E+06	5.69E+06	5.64E+06	5.23E+06	5.82E+06	4.48E+05	7.71	plasma	CD
Unkown	level4f420	467.3016	2.9	1.03E+07	9.55E+06	7.20E+06	9.70E+06	8.02E+06	8.96E+06	1.30E+06	14.49	plasma	LDA
Unkown	level4f420	467.3020	2.9	2.48E+06	2.55E+06	1.91E+06	2.12E+06	1.96E+06	2.20E+06	2.97E+05	13.47	plasma	XCMS
Unkown	level4f420	467.3016	2.9	1.79E+06	3.48E+06	2.23E+06	2.60E+06	1.93E+06	2.41E+06	6.77E+05	28.11	plasma	CD
Unkown	level4f421	474.2913	5.2	9.28E+07	8.33E+07	7.69E+07	8.01E+07	6.49E+07	7.96E+07	1.02E+07	12.78	plasma	LDA
Unkown	level4f421	474.2916	5.2	1.26E+07	6.49E+06	1.31E+07	1.20E+07	8.77E+06	1.06E+07	2.86E+06	26.97	plasma	XCMS
Unkown	level4f422	478.7854	2.6	1.03E+06	2.39E+06	2.11E+06	1.54E+06	1.62E+06	1.74E+06	5.28E+05	30.40	plasma	LDA
Unkown	level4f422	478.7854	2.6	4.21E+05	1.04E+06	3.95E+05	4.01E+05	4.36E+05	5.39E+05	2.83E+05	52.46	plasma	CD
Unkown	level4f424	487.2993	2.6	2.35E+07	1.99E+07	1.56E+07	2.15E+07	1.33E+07	1.88E+07	4.18E+06	22.28	plasma	LDA
Unkown	level4f424	487.2993	2.6	7.12E+06	6.32E+06	1.38E+07	6.56E+06	3.90E+06	7.54E+06	3.72E+06	49.27	plasma	CD
Unkown	level4f424	487.2995	2.7	5.67E+06	5.14E+06	3.83E+06	5.06E+06	3.05E+06	4.55E+06	1.08E+06	23.64	plasma	XCMS
Unkown	level4f425	487.2993	3.1	7.30E+07	6.10E+07	5.55E+07	6.37E+07	5.45E+07	6.15E+07	7.45E+06	12.12	plasma	LDA
Unkown	level4f425	487.2998	3.1	1.78E+07	1.46E+07	1.06E+07	1.58E+07	1.28E+07	1.43E+07	2.78E+06	19.39	plasma	XCMS
Unkown	level4f425	487.2993	3.1	2.35E+07	2.34E+07	1.38E+07	2.09E+07	1.73E+07	1.98E+07	4.17E+06	21.07	plasma	CD
Unkown	level4f426	487.2996	3.6	1.03E+08	1.07E+08	9.98E+07	9.82E+07	8.34E+07	9.82E+07	8.89E+06	9.05	plasma	LDA
Unkown	level4f426	487.2996	3.6	2.37E+07	2.51E+07	2.21E+07	2.83E+07	1.88E+07	2.36E+07	3.52E+06	14.91	plasma	XCMS
Unkown	level4f426	487.2996	3.6	3.89E+07	4.22E+07	2.87E+07	4.10E+07	3.23E+07	3.66E+07	5.88E+06	16.05	plasma	CD
Unkown	level4f427	487.8015	2.6	7.79E+06	8.86E+06	6.88E+06	6.94E+06	3.60E+06	6.81E+06	1.97E+06	28.92	plasma	LDA

Unkown	level4f427	487.8011	3.1	6.41E+06	4.48E+06	4.83E+06	4.86E+06	3.76E+06	4.87E+06	9.69E+05	19.91	plasma	XCMS
Unkown	level4f427	487.8015	2.6	1.77E+06	1.04E+06	1.38E+06	1.46E+06	6.26E+05	1.25E+06	4.36E+05	34.78	plasma	CD
Unkown	level4f428	489.3147	3.5	7.75E+06	7.55E+06	7.11E+06	7.00E+06	5.22E+06	6.92E+06	1.00E+06	14.48	plasma	LDA
Unkown	level4f428	489.3147	3.5	1.96E+06	1.75E+06	1.84E+06	1.83E+06	1.27E+06	1.73E+06	2.70E+05	15.59	plasma	XCMS
Unkown	level4f428	489.3147	3.5	2.97E+06	2.89E+06	2.35E+06	2.20E+06	1.68E+06	2.42E+06	5.28E+05	21.85	plasma	CD
Unkown	level4f429	489.3149	2.9	3.89E+06	3.70E+06	3.73E+06	3.56E+06	4.00E+06	3.78E+06	1.71E+05	4.53	plasma	LDA
Unkown	level4f429	489.3149	2.9	9.48E+05	8.18E+05	1.02E+06	5.61E+05	7.46E+05	8.19E+05	1.80E+05	21.96	plasma	CD
Unkown	level4f430	496.3049	5.2	1.13E+08	1.07E+08	1.02E+08	9.93E+07	8.48E+07	1.01E+08	1.05E+07	10.35	plasma	LDA
Unkown	level4f430	496.3047	5.2	1.37E+07	8.44E+06	7.83E+06	1.06E+07	8.02E+06	9.72E+06	2.48E+06	25.51	plasma	XCMS
Carnosine	level1f1	227.1139	9.1	8.43E+07	4.70E+07	4.70E+07	NA	NA	5.94E+07	2.16E+07	36.28	CSF	LDA
Carnosine	level1f1	227.1138	8.7	6.38E+04	1.52E+07	3.32E+04	NA	NA	5.09E+06	8.74E+06	171.56	CSF	XCMS
Alanine	level1f2	90.0549	17.4	5.18E+07	4.58E+07	4.58E+07	NA	NA	4.78E+07	3.44E+06	7.20	CSF	LDA
Alanine	level1f2	90.0549	16.7	4.23E+07	3.68E+07	3.76E+06	NA	NA	2.76E+07	2.08E+07	75.46	CSF	CD
Choline	level1f3	104.1072	7.5	3.44E+08	3.06E+08	3.06E+08	NA	NA	3.19E+08	2.21E+07	6.92	CSF	LDA
Choline	level1f3	104.1070	7.6	1.16E+08	9.04E+07	1.77E+07	NA	NA	7.48E+07	5.11E+07	68.33	CSF	CD
Serine	level1f4	106.0499	21.5	1.80E+07	1.48E+07	1.48E+07	NA	NA	1.59E+07	1.86E+06	11.71	CSF	LDA
Serine	level1f4	106.0495	21.1	9.80E+06	6.11E+06	1.11E+06	NA	NA	5.67E+06	4.36E+06	76.90	CSF	CD
Serine	level1f4	106.0500	21.0	2.43E+04	3.41E+06	3.09E+06	NA	NA	2.18E+06	1.87E+06	85.94	CSF	XCMS
Creatinine	level1f5	114.0662	9.0	6.23E+08	5.77E+08	5.39E+08	NA	NA	5.80E+08	4.21E+07	7.26	CSF	LDA
Creatinine	level1f5	114.0662	8.9	4.38E+08	3.57E+08	1.61E+08	NA	NA	3.19E+08	1.43E+08	44.82	CSF	CD
Creatinine	level1f5	114.0662	8.7	6.17E+07	1.73E+06	1.18E+08	NA	NA	6.05E+07	5.82E+07	96.22	CSF	XCMS
Proline	level1f6	116.0706	15.0	3.78E+06	3.49E+06	3.49E+06	NA	NA	3.59E+06	1.67E+05	4.66	CSF	LDA
Valine	level1f7	118.0862	11.0	6.93E+08	6.57E+08	6.57E+08	NA	NA	6.69E+08	2.09E+07	3.13	CSF	LDA
Valine	level1f7	118.0862	11.0	4.98E+07	2.52E+07	9.04E+06	NA	NA	2.80E+07	2.05E+07	73.22	CSF	CD
Taurine	level1f8	126.0219	15.3	1.37E+07	1.71E+07	1.47E+07	NA	NA	1.52E+07	1.77E+06	11.68	CSF	LDA
Taurine	level1f8	126.0222	14.9	2.82E+06	2.26E+04	3.19E+06	NA	NA	2.01E+06	1.73E+06	86.12	CSF	XCMS
Creatine	level1f9	132.0766	15.3	4.71E+08	4.16E+08	3.94E+08	NA	NA	4.27E+08	4.00E+07	9.37	CSF	LDA
Creatine	level1f9	132.0767	15.2	1.55E+08	1.39E+08	3.14E+07	NA	NA	1.08E+08	6.71E+07	61.93	CSF	CD
Isoleucine	level1f10	132.1017	10.3	2.14E+08	1.79E+08	1.79E+08	NA	NA	1.91E+08	2.05E+07	10.75	CSF	LDA
Leucine	level1f10	132.1017	10.3	7.17E+06	1.66E+07	2.27E+07	NA	NA	1.55E+07	7.84E+06	50.56	CSF	CD

Leucine	level1f10	132.1009	10.6	2.93E+04	7.77E+05	4.63E+05	NA	NA	4.23E+05	3.75E+05	88.74	CSF	XCMS
Leucine	level1f11	132.1019	10.0	1.17E+08	1.03E+08	1.03E+08	NA	NA	1.08E+08	8.19E+06	7.58	CSF	LDA
Isoleucine	level1f11	132.1024	10.6	1.59E+07	8.48E+06	2.27E+07	NA	NA	1.57E+07	7.12E+06	45.35	CSF	CD
Isoleucine	level1f11	132.1009	10.6	2.93E+04	7.77E+05	4.63E+05	NA	NA	4.23E+05	3.75E+05	88.74	CSF	XCMS
Asparagine	level1f12	133.0607	22.1	2.29E+07	2.19E+07	1.96E+07	NA	NA	2.15E+07	1.69E+06	7.86	CSF	LDA
Asparagine	level1f12	133.0606	21.8	1.96E+07	1.65E+07	1.71E+06	NA	NA	1.26E+07	9.59E+06	75.92	CSF	CD
Asparagine	level1f12	133.0610	21.5	3.64E+04	4.82E+06	3.61E+06	NA	NA	2.82E+06	2.49E+06	88.15	CSF	XCMS
Glutamine	level1f13	147.0764	21.3	1.47E+09	1.29E+09	1.30E+09	NA	NA	1.35E+09	1.02E+08	7.56	CSF	LDA
Glutamine	level1f13	147.0760	21.0	1.33E+09	1.08E+09	2.54E+08	NA	NA	8.88E+08	5.62E+08	63.36	CSF	CD
Glutamine	level1f13	147.0764	20.7	2.99E+08	2.97E+06	1.97E+08	NA	NA	1.66E+08	1.50E+08	90.44	CSF	XCMS
Lysine	level1f14	147.1128	30.5	3.76E+07	3.05E+07	2.41E+07	NA	NA	3.07E+07	6.75E+06	21.99	CSF	LDA
Lysine	level1f14	147.1126	30.8	1.25E+08	1.16E+08	1.50E+06	NA	NA	8.07E+07	6.87E+07	85.16	CSF	CD
Glutamate	level1f15	148.0604	19.3	2.53E+06	2.10E+06	1.71E+06	NA	NA	2.11E+06	4.12E+05	19.50	CSF	LDA
Methionine	level1f16	150.0583	12.1	9.00E+06	1.05E+07	1.05E+07	NA	NA	1.00E+07	8.84E+05	8.82	CSF	LDA
Methionine	level1f16	150.0583	11.5	5.41E+06	3.12E+06	1.30E+05	NA	NA	2.89E+06	2.65E+06	91.64	CSF	CD
Histidine	level1f17	156.0767	29.7	6.31E+07	4.39E+07	4.39E+07	NA	NA	5.03E+07	1.11E+07	22.01	CSF	LDA
Histidine	level1f17	156.0765	29.9	1.16E+08	7.38E+07	3.66E+04	NA	NA	6.33E+07	5.87E+07	92.74	CSF	CD
Carnitine	level1f18	162.1125	9.4	2.23E+08	1.48E+08	1.48E+08	NA	NA	1.73E+08	4.30E+07	24.87	CSF	LDA
Carnitine	level1f18	162.1126	8.8	5.80E+07	4.98E+07	3.64E+07	NA	NA	4.81E+07	1.09E+07	22.64	CSF	CD
Carnitine	level1f18	162.1126	8.9	7.60E+07	5.40E+07	3.52E+07	NA	NA	5.51E+07	2.04E+07	37.08	CSF	XCMS
Phenylalanine	level1f19	166.0862	11.2	1.41E+08	9.98E+07	9.98E+07	NA	NA	1.14E+08	2.39E+07	21.05	CSF	LDA
Phenylalanine	level1f19	166.0863	10.6	5.93E+07	4.40E+07	1.28E+07	NA	NA	3.87E+07	2.37E+07	61.31	CSF	CD
Phenylalanine	level1f19	166.0865	10.9	2.74E+07	3.03E+07	2.29E+07	NA	NA	2.68E+07	3.73E+06	13.92	CSF	XCMS
Arginine	level1f20	175.1189	29.3	2.74E+08	2.46E+08	2.53E+08	NA	NA	2.57E+08	1.44E+07	5.60	CSF	LDA
Arginine	level1f20	175.1186	29.9	2.42E+08	2.07E+08	8.81E+03	NA	NA	1.49E+08	1.31E+08	87.40	CSF	CD
Arginine	level1f20	175.1271	28.7	2.78E+04	3.01E+04	9.10E+06	NA	NA	3.05E+06	5.23E+06	171.56	CSF	XCMS
Citrulline	level1f21	176.1029	22.9	8.82E+06	7.58E+06	8.70E+06	NA	NA	8.37E+06	6.83E+05	8.16	CSF	LDA
Citrulline	level1f21	176.1025	22.6	1.02E+07	8.49E+06	4.80E+05	NA	NA	6.38E+06	5.17E+06	81.14	CSF	CD
Methioninesulfoxide	level1f22	166.0532	21.2	2.58E+06	1.50E+06	1.50E+06	NA	NA	1.86E+06	6.19E+05	33.26	CSF	LDA
Methioninesulfoxide	level1f22	166.0529	21.0	8.20E+05	2.75E+05	3.81E+05	NA	NA	4.92E+05	2.89E+05	58.73	CSF	XCMS

Threonine	level1f23	120.0655	19.1	1.10E+08	9.89E+07	1.00E+08	NA	NA	1.03E+08	6.10E+06	5.91	CSF	LDA
Threonine	level1f23	120.0653	18.8	1.13E+08	8.18E+07	1.21E+07	NA	NA	6.90E+07	5.17E+07	74.90	CSF	CD
Threonine	level1f23	120.0652	18.5	1.93E+05	2.44E+07	1.45E+07	NA	NA	1.31E+07	1.22E+07	93.35	CSF	XCMS
L-Pyroglutamic acid	level2f1	130.0500	4.0	4.39E+07	4.66E+07	4.66E+07	NA	NA	4.57E+07	1.54E+06	3.38	CSF	LDA
L-Pyroglutamic acid	level2f1	130.0500	4.1	4.06E+07	4.11E+07	1.83E+07	NA	NA	3.33E+07	1.30E+07	39.09	CSF	CD
L-Pyroglutamic acid	level2f1	130.0497	3.8	2.49E+06	9.70E+06	9.37E+04	NA	NA	4.09E+06	5.00E+06	122.16	CSF	XCMS
5-Aminolevulinic acid	level2f2	132.0655	3.0	1.36E+07	1.08E+07	1.08E+07	NA	NA	1.17E+07	1.64E+06	13.96	CSF	LDA
5-Aminolevulinic acid	level2f2	132.0655	3.1	2.80E+07	2.14E+07	6.70E+05	NA	NA	1.67E+07	1.43E+07	85.46	CSF	CD
5-Aminolevulinic acid	level2f2	132.0655	2.9	1.45E+06	2.46E+06	2.12E+06	NA	NA	2.01E+06	5.13E+05	25.55	CSF	XCMS
Acetylcholine	level2f3	146.1174	7.8	7.47E+06	5.97E+06	5.18E+06	NA	NA	6.21E+06	1.17E+06	18.79	CSF	LDA
Acetylcholine	level2f3	146.1174	7.7	4.81E+06	3.92E+06	5.38E+05	NA	NA	3.09E+06	2.26E+06	72.98	CSF	CD
1-Methylguanine	level2f4	166.0720	8.6	5.64E+06	4.18E+06	4.18E+06	NA	NA	4.67E+06	8.42E+05	18.02	CSF	LDA
1-Methylguanine	level2f4	166.0720	9.2	6.13E+06	1.27E+07	5.38E+06	NA	NA	8.07E+06	4.03E+06	49.95	CSF	CD
1-Methylguanine	level2f4	166.0719	8.6	3.97E+06	1.16E+06	1.03E+06	NA	NA	2.05E+06	1.66E+06	80.96	CSF	XCMS
Theophylline @RT:3.051	level2f5	181.0716	2.9	3.66E+07	3.28E+07	3.28E+07	NA	NA	3.41E+07	2.19E+06	6.42	CSF	LDA
Theophylline @RT:3.051	level2f5	181.0722	3.0	1.03E+06	8.11E+05	1.61E+07	NA	NA	5.99E+06	8.79E+06	146.59	CSF	CD
Theophylline @RT:3.051	level2f5	181.0717	2.8	3.44E+06	7.21E+06	6.35E+06	NA	NA	5.67E+06	1.98E+06	34.84	CSF	XCMS
Indole-3-acrylic acid	level2f6	188.0702	11.3	5.96E+07	3.66E+07	4.42E+07	NA	NA	4.68E+07	1.17E+07	25.03	CSF	LDA
Indole-3-acrylic acid	level2f6	188.0702	10.6	1.15E+07	3.24E+06	1.08E+06	NA	NA	5.27E+06	5.49E+06	104.27	CSF	CD
Indole-3-acrylic acid	level2f6	188.0696	11.0	8.70E+04	1.18E+07	8.67E+06	NA	NA	6.86E+06	6.07E+06	88.51	CSF	XCMS
N6,N6,N6-Trimethyl-L-lysine	level2f7	189.1594	27.2	2.27E+08	1.98E+08	2.05E+08	NA	NA	2.10E+08	1.51E+07	7.19	CSF	LDA
N6,N6,N6-Trimethyl-L-lysine	level2f7	189.1594	28.2	1.18E+07	9.69E+06	4.34E+05	NA	NA	7.31E+06	6.04E+06	82.73	CSF	CD
Pantothenic acid	level2f8	220.1178	3.3	5.42E+06	3.51E+06	3.51E+06	NA	NA	4.15E+06	1.11E+06	26.68	CSF	LDA
Pantothenic acid	level2f8	220.1179	3.6	1.50E+06	8.41E+05	1.02E+05	NA	NA	8.13E+05	6.97E+05	85.80	CSF	CD
Pantothenic acid	level2f8	220.1177	3.2	9.22E+05	1.38E+06	8.31E+05	NA	NA	1.04E+06	2.92E+05	27.97	CSF	XCMS
N-Acetylneuraminicacid(NeuAc)	level2f9	310.1128	17.7	2.88E+07	3.38E+07	2.98E+07	NA	NA	3.08E+07	2.67E+06	8.67	CSF	LDA
N-Acetylneuraminicacid(NeuAc)	level2f9	310.1128	16.5	5.88E+07	9.59E+07	7.01E+06	NA	NA	5.39E+07	4.46E+07	82.81	CSF	CD
N-Methylpyrrolidone @RT:3.152	level3f2	100.0754	2.6	4.67E+07	4.26E+07	4.26E+07	NA	NA	4.40E+07	2.37E+06	5.39	CSF	LDA
N-Methylpyrrolidone @RT:3.152	level3f2	100.0757	2.7	2.61E+07	1.49E+07	6.42E+07	NA	NA	3.51E+07	2.58E+07	73.72	CSF	CD
N-Methylpyrrolidone @RT:3.152	level3f2	100.0632	2.5	1.43E+07	8.11E+04	1.23E+07	NA	NA	8.91E+06	7.72E+06	86.57	CSF	XCMS

	104680	level3f3	84.0441	21.3	2.57E+07	2.24E+07	2.24E+07	NA	NA	2.35E+07	1.94E+06	8.28	CSF	LDA
	104680	level3f3	84.0440	21.0	1.58E+07	1.56E+07	2.95E+06	NA	NA	1.15E+07	7.38E+06	64.34	CSF	CD
	104680	level3f3	84.0442	20.7	5.30E+06	4.67E+06	4.89E+06	NA	NA	4.95E+06	3.21E+05	6.47	CSF	XCMS
Selsun @RT:10.584		level3f3	112.8963	10.2	4.99E+08	3.01E+08	3.01E+08	NA	NA	3.67E+08	1.14E+08	31.01	CSF	LDA
Selsun @RT:10.584		level3f3	112.8956	10.1	1.23E+08	4.64E+06	3.94E+07	NA	NA	5.56E+07	6.08E+07	109.21	CSF	CD
Selsun @RT:10.584		level3f3	112.8956	10.1	4.21E+07	1.33E+06	4.84E+07	NA	NA	3.06E+07	2.56E+07	83.50	CSF	XCMS
Nipam		level3f4	114.0916	2.5	1.97E+07	1.63E+07	1.63E+07	NA	NA	1.75E+07	1.98E+06	11.33	CSF	LDA
Nipam		level3f4	114.0915	2.5	2.58E+07	7.03E+06	8.89E+06	NA	NA	1.39E+07	1.03E+07	74.25	CSF	CD
Nipam		level3f4	114.0913	2.4	4.36E+06	5.67E+06	2.29E+06	NA	NA	4.11E+06	1.70E+06	41.50	CSF	XCMS
n-Hexanamide		level3f5	116.1069	7.0	2.75E+07	3.31E+07	3.31E+07	NA	NA	3.12E+07	3.25E+06	10.41	CSF	LDA
n-Hexanamide		level3f5	116.1070	7.1	6.55E+07	5.15E+07	1.96E+07	NA	NA	4.55E+07	2.35E+07	51.71	CSF	CD
Indole;1-Benzazole		level3f6	118.0658	11.4	2.27E+07	2.01E+07	2.11E+07	NA	NA	2.13E+07	1.30E+06	6.10	CSF	LDA
Indole;1-Benzazole		level3f6	118.0654	10.8	3.93E+06	4.09E+06	3.28E+06	NA	NA	3.77E+06	4.28E+05	11.38	CSF	XCMS
Indole;1-Benzazole		level3f6	118.0658	10.6	9.27E+06	9.79E+06	2.89E+06	NA	NA	7.32E+06	3.84E+06	52.50	CSF	CD
[FAamino(5:0)]2S-amino-pentanoicacid		level3f7	118.0864	14.9	2.15E+07	2.12E+07	2.12E+07	NA	NA	2.13E+07	2.14E+05	1.00	CSF	LDA
[FAamino(5:0)]2S-amino-pentanoicacid		level3f7	118.0861	15.1	7.82E+06	1.20E+07	3.60E+06	NA	NA	7.80E+06	4.18E+06	53.68	CSF	CD
[FAamino(5:0)]2S-amino-pentanoicacid		level3f7	118.0861	14.4	4.86E+06	1.01E+06	3.60E+06	NA	NA	3.16E+06	1.96E+06	62.21	CSF	XCMS
Indoline @RT:10.521		level3f8	120.0806	11.2	1.07E+08	8.01E+07	8.01E+07	NA	NA	8.92E+07	1.58E+07	17.71	CSF	LDA
Indoline @RT:10.521		level3f8	120.0808	10.6	4.58E+07	3.25E+07	5.69E+06	NA	NA	2.80E+07	2.04E+07	72.96	CSF	CD
Indoline @RT:10.521		level3f8	120.0810	10.9	1.67E+07	2.39E+07	1.80E+07	NA	NA	1.95E+07	3.84E+06	19.65	CSF	XCMS
	107089	level3f9	124.1120	5.1	4.44E+06	5.95E+06	5.45E+06	NA	NA	5.28E+06	7.71E+05	14.61	CSF	LDA
	107089	level3f9	124.1121	5.4	1.86E+07	1.40E+07	8.91E+06	NA	NA	1.38E+07	4.83E+06	34.91	CSF	CD
	107089	level3f9	124.1121	4.5	1.03E+06	9.89E+05	8.66E+05	NA	NA	9.62E+05	8.63E+04	8.97	CSF	XCMS
Pivaloylacetonitrile @RT:2.934		level3f10	126.0912	3.2	1.72E+07	1.39E+07	1.39E+07	NA	NA	1.50E+07	1.90E+06	12.66	CSF	LDA
Pivaloylacetonitrile @RT:2.934		level3f10	126.0916	2.8	2.88E+07	2.13E+07	4.74E+07	NA	NA	3.25E+07	1.34E+07	41.27	CSF	CD
Pivaloylacetonitrile @RT:2.934		level3f10	126.0915	3.2	3.96E+06	4.05E+06	2.66E+06	NA	NA	3.56E+06	7.77E+05	21.84	CSF	XCMS
3-Guanidinopropanoate @RT:10.972		level3f11	132.0770	10.9	1.08E+08	1.39E+08	1.09E+08	NA	NA	1.18E+08	1.75E+07	14.75	CSF	LDA
3-Guanidinopropanoate		level3f11	132.0755	11.8	3.49E+06	1.47E+05	1.93E+05	NA	NA	1.28E+06	1.92E+06	150.18	CSF	XCMS

@RT:10.972													
3-Guanidinopropanoate @RT:10.972	level3f11	132.0770	11.0	3.61E+06	1.28E+06	1.69E+07	NA	NA	7.25E+06	8.40E+06	115.87	CSF	CD
allopurinol @RT:6.877	level3f12	137.0457	6.7	2.10E+08	1.86E+08	1.86E+08	NA	NA	1.94E+08	1.43E+07	7.36	CSF	LDA
allopurinol @RT:6.877	level3f12	137.0457	6.9	1.05E+07	1.01E+08	6.36E+03	NA	NA	3.70E+07	5.53E+07	149.38	CSF	CD
allopurinol @RT:9.326	level3f13	137.0460	9.5	9.22E+06	7.61E+06	1.30E+07	NA	NA	9.94E+06	2.77E+06	27.87	CSF	LDA
allopurinol @RT:9.326	level3f13	137.0460	9.3	1.01E+07	7.82E+06	4.42E+06	NA	NA	7.44E+06	2.85E+06	38.31	CSF	CD
allopurinol @RT:9.326	level3f13	137.0459	9.4	1.33E+06	1.48E+06	9.94E+05	NA	NA	1.27E+06	2.49E+05	19.67	CSF	XCMS
4-Nitroaniline	level3f14	139.0503	6.4	2.20E+06	7.51E+05	7.51E+05	NA	NA	1.23E+06	8.38E+05	67.84	CSF	LDA
4-Nitroaniline	level3f14	139.0503	7.7	4.09E+06	5.03E+06	3.45E+06	NA	NA	4.19E+06	7.94E+05	18.94	CSF	CD
Crotono-betaine @RT:7.108	level3f15	142.1222	6.6	2.24E+07	3.73E+07	3.66E+07	NA	NA	3.21E+07	8.44E+06	26.29	CSF	LDA
Crotono-betaine @RT:7.108	level3f15	142.1222	7.1	3.11E+05	1.62E+06	6.93E+06	NA	NA	2.95E+06	3.50E+06	118.74	CSF	CD
Stachydrine @RT:10.878	level3f16	144.1018	11.6	2.45E+08	2.18E+08	1.60E+08	NA	NA	2.07E+08	4.36E+07	21.01	CSF	LDA
Stachydrine @RT:10.878	level3f16	144.1019	11.1	5.08E+06	1.17E+06	1.34E+05	NA	NA	2.13E+06	2.61E+06	122.50	CSF	CD
Stachydrine @RT:10.878	level3f16	144.1022	11.1	2.17E+07	3.33E+04	3.44E+07	NA	NA	1.87E+07	1.74E+07	92.86	CSF	XCMS
Xanthine @RT:8.398	level3f17	153.0404	8.3	3.87E+07	3.91E+07	3.60E+07	NA	NA	3.79E+07	1.68E+06	4.42	CSF	LDA
Xanthine @RT:8.398	level3f17	153.0407	7.9	1.80E+03	1.49E+03	1.40E+03	NA	NA	1.56E+03	2.09E+02	13.35	CSF	CD
Xanthine @RT:8.398	level3f17	153.0405	7.9	9.67E+04	1.32E+05	8.16E+06	NA	NA	2.80E+06	4.65E+06	166.12	CSF	XCMS
N1-Methyl-2-pyridone-5-carboxamide	level3f18	153.0659	3.8	2.60E+07	2.57E+07	2.06E+07	NA	NA	2.41E+07	3.05E+06	12.64	CSF	LDA
N1-Methyl-2-pyridone-5-carboxamide	level3f18	153.0659	3.8	4.18E+07	3.84E+07	1.06E+07	NA	NA	3.02E+07	1.71E+07	56.64	CSF	CD
N1-Methyl-2-pyridone-5-carboxamide	level3f18	153.0659	3.6	8.87E+05	5.26E+06	2.16E+04	NA	NA	2.06E+06	2.81E+06	136.58	CSF	XCMS
TO0127900	level3f19	156.1383	6.4	6.59E+06	1.52E+07	1.43E+07	NA	NA	1.20E+07	4.73E+06	39.36	CSF	LDA
TO0127900	level3f19	156.1383	6.5	1.33E+07	7.64E+06	1.59E+07	NA	NA	1.23E+07	4.24E+06	34.49	CSF	CD
L-Carnitinamide	level3f20	161.1285	28.5	1.03E+06	8.03E+05	8.03E+05	NA	NA	8.80E+05	1.34E+05	15.19	CSF	LDA
L-Carnitinamide	level3f20	161.1283	29.2	1.05E+07	8.62E+06	6.10E+03	NA	NA	6.37E+06	5.59E+06	87.75	CSF	CD
Ascorbicacid	level3f21	177.0393	11.4	7.19E+05	6.82E+05	7.44E+05	NA	NA	7.15E+05	3.11E+04	4.36	CSF	LDA
Ascorbicacid	level3f21	177.0435	11.3	3.00E+05	2.31E+05	1.92E+05	NA	NA	2.41E+05	5.51E+04	22.86	CSF	XCMS
apronalide @RT:3.474	level3f22	185.1281	3.2	4.80E+07	3.76E+07	3.76E+07	NA	NA	4.11E+07	6.02E+06	14.65	CSF	LDA
apronalide @RT:3.474	level3f22	185.1285	2.9	5.62E+07	4.05E+07	4.23E+04	NA	NA	3.23E+07	2.90E+07	89.85	CSF	CD

apronalide @RT:3.474	level3f22	185.1285	3.2	9.64E+06	1.09E+07	7.09E+06	NA	NA	9.22E+06	1.96E+06	21.21	CSF	XCMS
Homoarginine	level3f23	189.1345	29.0	6.95E+05	5.08E+05	5.08E+05	NA	NA	5.70E+05	1.08E+05	18.97	CSF	LDA
Homoarginine	level3f23	189.1345	29.5	2.44E+06	2.20E+06	1.87E+04	NA	NA	1.55E+06	1.33E+06	85.89	CSF	CD
N-Acetyl-L-histidine	level3f24	198.0871	16.5	2.05E+07	2.24E+07	2.24E+07	NA	NA	2.17E+07	1.10E+06	5.07	CSF	LDA
N-Acetyl-L-histidine	level3f24	198.0871	16.0	1.06E+07	9.05E+06	9.98E+05	NA	NA	6.89E+06	5.17E+06	74.95	CSF	CD
Symmetricdimethylarginine	level3f25	203.1497	25.3	3.79E+07	3.91E+07	4.08E+07	NA	NA	3.92E+07	1.46E+06	3.72	CSF	LDA
Mutagen X	level3f26	216.9221	13.3	4.07E+07	4.17E+07	4.17E+07	NA	NA	4.14E+07	5.69E+05	1.37	CSF	LDA
Mutagen X	level3f26	216.9224	13.8	3.76E+07	2.43E+07	6.33E+06	NA	NA	2.27E+07	1.57E+07	68.94	CSF	CD
Mutagen X	level3f26	216.9223	13.1	1.07E+07	9.71E+06	8.88E+06	NA	NA	9.78E+06	9.32E+05	9.53	CSF	XCMS
(E)-Furazolidone	level3f27	226.0450	15.6	1.00E+07	7.63E+06	7.63E+06	NA	NA	8.42E+06	1.37E+06	16.23	CSF	LDA
(E)-Furazolidone	level3f27	226.0450	15.2	2.32E+06	2.21E+06	2.52E+06	NA	NA	2.35E+06	1.58E+05	6.72	CSF	CD
Bis(carboxymethyl)trithiocarbonate	level3f28	226.9514	14.7	1.40E+09	1.40E+09	1.41E+09	NA	NA	1.40E+09	9.97E+06	0.71	CSF	LDA
Bis(carboxymethyl)trithiocarbonate	level3f28	226.9511	14.8	5.96E+08	4.34E+08	2.15E+08	NA	NA	4.15E+08	1.91E+08	46.08	CSF	CD
Bis(carboxymethyl)trithiocarbonate	level3f28	226.9517	14.2	6.18E+05	8.90E+07	1.28E+08	NA	NA	7.26E+07	6.53E+07	89.99	CSF	XCMS
4-Nonanoylmorpholine @RT:2.086	level3f29	228.1963	2.0	4.10E+06	8.63E+06	8.63E+06	NA	NA	7.12E+06	2.62E+06	36.73	CSF	LDA
4-Nonanoylmorpholine @RT:2.086	level3f29	228.1961	2.1	4.73E+06	4.13E+06	2.54E+07	NA	NA	1.14E+07	1.21E+07	106.05	CSF	CD
Leu-Pro(Leucyl-Proline) @RT:8.207	level3f30	229.1540	8.2	1.48E+07	1.02E+07	1.02E+07	NA	NA	1.17E+07	2.65E+06	22.61	CSF	LDA
Leu-Pro(Leucyl-Proline) @RT:8.207	level3f30	229.1540	8.3	4.02E+06	1.97E+06	1.91E+05	NA	NA	2.06E+06	1.92E+06	92.93	CSF	CD
Alanylalanylalanine	level3f31	232.1288	20.9	3.21E+06	2.49E+06	2.49E+06	NA	NA	2.73E+06	4.17E+05	15.26	CSF	LDA
Alanylalanylalanine	level3f31	232.1288	20.6	4.73E+06	4.58E+06	1.87E+05	NA	NA	3.17E+06	2.58E+06	81.53	CSF	CD
Ethyl 4-[(2-methoxy-2-oxoethyl)carbamoyl]-1H-imidazole-5-carboxylate	level3f32	256.0924	18.1	6.72E+06	6.33E+06	6.33E+06	NA	NA	6.46E+06	2.25E+05	3.49	CSF	LDA
Ethyl 4-[(2-methoxy-2-oxoethyl)carbamoyl]-1H-imidazole-5-carboxylate	level3f32	256.0925	17.9	4.90E+06	5.05E+06	3.58E+05	NA	NA	3.44E+06	2.67E+06	77.62	CSF	CD
Lotaustralin	level3f33	262.1288	9.1	3.18E+06	4.01E+06	4.01E+06	NA	NA	3.73E+06	4.84E+05	12.96	CSF	LDA
Lotaustralin	level3f33	262.1286	8.6	1.71E+07	1.74E+07	5.60E+06	NA	NA	1.34E+07	6.73E+06	50.33	CSF	CD
2 7-Anhydro-alpha-N-acetylneuraminicacid	level3f34	292.1031	17.8	2.40E+07	2.47E+07	2.86E+07	NA	NA	2.57E+07	2.47E+06	9.60	CSF	LDA
2 7-Anhydro-alpha-N-acetylneuraminicacid	level3f34	292.1031	16.5	3.77E+07	4.00E+07	5.11E+07	NA	NA	4.29E+07	7.15E+06	16.66	CSF	CD

[3-(4-methylbenzoyl)isoxazol-4-yl](thien-2-yl)methanone	level3f35	298.0528	32.9	1.85E+05	1.35E+05	1.44E+05	NA	NA	1.55E+05	2.68E+04	17.29	CSF	LDA
[3-(4-methylbenzoyl)isoxazol-4-yl](thien-2-yl)methanone	level3f35	298.0528	32.3	4.15E+06	3.36E+06	3.05E+03	NA	NA	2.50E+06	2.20E+06	87.92	CSF	CD
Dimantine	level3f36	298.3472	2.6	2.10E+05	1.72E+05	1.72E+05	NA	NA	1.84E+05	2.21E+04	11.96	CSF	LDA
Dimantine	level3f36	298.3472	2.6	1.40E+05	1.55E+05	5.82E+06	NA	NA	2.04E+06	3.27E+06	160.67	CSF	CD
N-Allyl-3,5-dimethoxy-N-[5-(4-pyridinyl)-1,3,4-thiadiazol-2-yl]benzamide	level3f37	383.1162	15.7	8.54E+06	8.50E+06	6.88E+06	NA	NA	7.97E+06	9.49E+05	11.90	CSF	LDA
N-Allyl-3,5-dimethoxy-N-[5-(4-pyridinyl)-1,3,4-thiadiazol-2-yl]benzamide	level3f37	383.1162	15.5	7.83E+06	4.48E+06	2.11E+06	NA	NA	4.81E+06	2.87E+06	59.74	CSF	CD
Piperidine	level3f38	86.0968	10.8	3.24E+07	2.70E+07	2.70E+07	NA	NA	2.88E+07	3.14E+06	10.91	CSF	LDA
Piperidine	level3f38	86.0968	10.5	9.94E+03	6.70E+05	1.14E+05	NA	NA	2.65E+05	3.55E+05	134.10	CSF	CD
Unkown	level4f1	114.8929	10.1	2.45E+08	3.26E+08	2.17E+08	NA	NA	2.63E+08	5.65E+07	21.50	CSF	LDA
Unkown	level4f1	114.8928	10.1	6.15E+07	9.96E+07	2.10E+07	NA	NA	6.07E+07	3.93E+07	64.78	CSF	CD
Unkown	level4f1	114.8931	10.0	2.11E+07	9.75E+05	2.39E+07	NA	NA	1.54E+07	1.25E+07	81.61	CSF	XCMS
Unkown	level4f2	121.0722	4.4	6.39E+07	4.51E+07	4.51E+07	NA	NA	5.13E+07	1.09E+07	21.16	CSF	LDA
Unkown	level4f2	121.0723	4.8	1.47E+08	1.39E+08	2.76E+07	NA	NA	1.05E+08	6.69E+07	63.89	CSF	CD
Unkown	level4f2	121.0719	4.3	2.89E+04	1.46E+07	1.03E+07	NA	NA	8.29E+06	7.47E+06	90.11	CSF	XCMS
Unkown	level4f3	151.1436	7.4	1.56E+07	1.15E+07	9.50E+06	NA	NA	1.22E+07	3.13E+06	25.61	CSF	LDA
Unkown	level4f3	151.1440	7.4	2.96E+07	2.00E+07	2.54E+06	NA	NA	1.74E+07	1.37E+07	78.96	CSF	CD
Unkown	level4f4	158.9636	14.7	7.32E+08	7.08E+08	7.08E+08	NA	NA	7.16E+08	1.38E+07	1.93	CSF	LDA
Unkown	level4f4	158.9637	14.8	3.26E+08	2.81E+08	2.01E+08	NA	NA	2.69E+08	6.33E+07	23.51	CSF	CD
Unkown	level4f4	158.9635	14.3	7.43E+07	4.60E+07	7.88E+07	NA	NA	6.64E+07	1.78E+07	26.81	CSF	XCMS
Unkown	level4f5	181.0079	14.8	4.21E+07	9.45E+07	9.45E+07	NA	NA	7.70E+07	3.03E+07	39.32	CSF	LDA
Unkown	level4f5	181.0079	15.1	1.24E+07	1.38E+07	1.01E+04	NA	NA	8.72E+06	7.58E+06	86.90	CSF	CD
Unkown	level4f5	181.0072	14.3	6.23E+06	6.95E+06	1.69E+06	NA	NA	4.96E+06	2.85E+06	57.48	CSF	XCMS
Unkown	level4f6	192.0553	22.9	3.83E+06	2.11E+06	2.11E+06	NA	NA	2.68E+06	9.95E+05	37.10	CSF	LDA
Unkown	level4f6	192.0553	22.7	4.64E+07	4.19E+07	3.54E+06	NA	NA	3.06E+07	2.36E+07	76.94	CSF	CD
Unkown	level4f7	200.0433	22.9	1.36E+06	2.39E+06	2.39E+06	NA	NA	2.05E+06	5.97E+05	29.19	CSF	LDA
Unkown	level4f8	203.0525	14.9	1.89E+07	1.93E+07	1.93E+07	NA	NA	1.92E+07	2.50E+05	1.30	CSF	LDA

Unkown	level4f8	203.0526	15.5	4.52E+07	2.53E+07	6.14E+06	NA	NA	2.56E+07	1.95E+07	76.47	CSF	CD
Unkown	level4f8	203.0523	15.1	4.05E+06	7.86E+05	4.54E+06	NA	NA	3.12E+06	2.04E+06	65.29	CSF	XCMS
Unkown	level4f9	205.0677	14.8	1.52E+07	1.20E+07	1.87E+07	NA	NA	1.53E+07	3.32E+06	21.68	CSF	LDA
Unkown	level4f9	205.0677	15.2	5.04E+06	3.66E+06	6.24E+03	NA	NA	2.90E+06	2.60E+06	89.63	CSF	CD
Unkown	level4f10	205.1547	28.9	9.91E+07	9.47E+07	9.55E+07	NA	NA	9.64E+07	2.36E+06	2.44	CSF	LDA
Unkown	level4f10	205.1546	29.5	4.67E+06	4.51E+06	4.15E+04	NA	NA	3.07E+06	2.63E+06	85.47	CSF	CD
Unkown	level4f11	274.0918	17.8	4.39E+07	4.42E+07	5.12E+07	NA	NA	4.64E+07	4.15E+06	8.93	CSF	LDA
Unkown	level4f12	276.1190	24.8	2.81E+07	2.43E+07	2.43E+07	NA	NA	2.56E+07	2.23E+06	8.71	CSF	LDA
Unkown	level4f12	276.1186	24.6	1.68E+07	1.47E+07	7.05E+05	NA	NA	1.07E+07	8.73E+06	81.51	CSF	CD
Unkown	level4f12	276.1051	24.1	3.72E+06	1.18E+04	1.07E+04	NA	NA	1.25E+06	2.14E+06	171.65	CSF	XCMS
Unkown	level4f13	290.0750	22.9	1.67E+05	1.65E+05	2.15E+05	NA	NA	1.82E+05	2.84E+04	15.55	CSF	LDA
Unkown	level4f13	290.0750	22.8	1.79E+06	1.06E+07	7.22E+04	NA	NA	4.14E+06	5.63E+06	135.94	CSF	CD
Unkown	level4f14	294.9388	14.7	2.28E+08	2.26E+08	2.26E+08	NA	NA	2.26E+08	1.20E+06	0.53	CSF	LDA
Unkown	level4f14	294.9386	14.8	1.13E+08	7.78E+07	4.09E+07	NA	NA	7.71E+07	3.59E+07	46.49	CSF	CD
Unkown	level4f14	294.9381	14.3	2.20E+05	1.55E+07	1.53E+05	NA	NA	5.30E+06	8.86E+06	167.12	CSF	XCMS
Unkown	level4f15	316.9824	15.0	4.73E+07	3.92E+07	3.96E+07	NA	NA	4.20E+07	4.60E+06	10.94	CSF	LDA
Unkown	level4f15	316.9827	15.0	1.06E+07	1.08E+07	6.30E+03	NA	NA	7.14E+06	6.18E+06	86.54	CSF	CD
Unkown	level4f16	347.0644	21.4	1.02E+07	1.20E+05	9.67E+04	NA	NA	3.46E+06	5.80E+06	167.77	CSF	LDA
Unkown	level4f16	347.0644	20.9	2.78E+07	1.45E+05	2.49E+06	NA	NA	1.01E+07	1.53E+07	151.14	CSF	CD
Unkown	level4f16	347.0647	20.7	8.90E+06	1.93E+06	2.68E+04	NA	NA	3.62E+06	4.67E+06	129.14	CSF	XCMS
Unkown	level4f17	362.9264	14.7	5.06E+08	5.05E+08	5.05E+08	NA	NA	5.05E+08	7.05E+05	0.14	CSF	LDA
Unkown	level4f17	362.9260	14.7	8.47E+07	1.40E+08	3.07E+07	NA	NA	8.52E+07	5.48E+07	64.32	CSF	CD
Unkown	level4f17	362.9255	14.2	7.18E+07	5.83E+07	4.50E+07	NA	NA	5.84E+07	1.34E+07	23.00	CSF	XCMS
Unkown	level4f18	420.8843	13.4	1.86E+07	1.63E+07	1.66E+07	NA	NA	1.72E+07	1.28E+06	7.45	CSF	LDA
Unkown	level4f18	420.8847	13.9	1.98E+07	1.63E+07	8.64E+06	NA	NA	1.49E+07	5.70E+06	38.25	CSF	CD
Unkown	level4f18	420.8846	13.2	3.34E+06	3.25E+06	3.52E+06	NA	NA	3.37E+06	1.39E+05	4.11	CSF	XCMS
Unkown	level4f19	430.9134	14.7	8.63E+08	8.88E+08	8.88E+08	NA	NA	8.80E+08	1.45E+07	1.65	CSF	LDA
Unkown	level4f19	430.9134	14.0	1.09E+08	1.03E+07	9.65E+07	NA	NA	7.19E+07	5.37E+07	74.67	CSF	CD
Unkown	level4f19	430.9129	14.2	1.20E+08	9.76E+07	7.73E+07	NA	NA	9.83E+07	2.14E+07	21.77	CSF	XCMS
Unkown	level4f20	498.9006	14.7	5.27E+08	5.42E+08	5.42E+08	NA	NA	5.37E+08	8.79E+06	1.64	CSF	LDA

Unkown	level4f20	498.9009	14.6	2.34E+08	1.27E+08	5.04E+07	NA	NA	1.37E+08	9.20E+07	67.08	CSF	CD
Unkown	level4f20	498.9003	14.3	7.50E+07	3.43E+07	7.27E+07	NA	NA	6.07E+07	2.29E+07	37.71	CSF	XCMS
Unkown	level4f21	566.8885	14.6	2.55E+08	2.56E+08	2.56E+08	NA	NA	2.56E+08	4.95E+05	0.19	CSF	LDA
Unkown	level4f21	566.8880	14.8	5.23E+07	6.52E+07	2.31E+07	NA	NA	4.69E+07	2.16E+07	46.10	CSF	CD
Unkown	level4f21	566.8875	14.3	2.22E+07	3.19E+07	3.68E+07	NA	NA	3.03E+07	7.47E+06	24.65	CSF	XCMS
Unkown	level4f22	634.8746	14.6	1.63E+08	1.68E+08	1.68E+08	NA	NA	1.66E+08	2.95E+06	1.78	CSF	LDA
Unkown	level4f22	634.8751	14.7	4.20E+07	3.97E+07	2.64E+07	NA	NA	3.60E+07	8.40E+06	23.32	CSF	CD
Unkown	level4f22	634.8746	14.3	1.96E+07	2.19E+07	2.57E+07	NA	NA	2.24E+07	3.09E+06	13.82	CSF	XCMS
Unkown	level4f23	702.8629	14.6	9.92E+07	9.69E+07	9.69E+07	NA	NA	9.77E+07	1.34E+06	1.38	CSF	LDA
Unkown	level4f23	702.8629	14.8	3.06E+07	1.83E+07	1.25E+07	NA	NA	2.05E+07	9.23E+06	45.13	CSF	CD
Unkown	level4f23	702.8615	14.3	1.26E+07	1.35E+07	6.60E+06	NA	NA	1.09E+07	3.77E+06	34.49	CSF	XCMS
Unkown	level4f24	123.0407	15.5	4.39E+06	7.93E+06	5.48E+06	NA	NA	5.93E+06	1.82E+06	30.63	CSF	LDA
Unkown	level4f24	123.0554	15.2	7.24E+05	1.74E+06	5.39E+04	NA	NA	8.40E+05	8.50E+05	101.20	CSF	XCMS

Table S2: List of parameters used for data processing with XCMS online (version of XCMS online 2.7.2, XCMS 1.47.3, CAMERA 1.34.0)

Parameters	Values
Parameter ID	HPLC/Orbitrap (136)
**Feature detection**	
method	centWave
ppm	3
snthr	6
peakwidth	10 60
mzdifference	0.01
prefilter peaks	3
prefilter intensity	100
noise	0
**Retention time correction**	
method	orbiwarp
profStep	1
**Grouping**	
method	density
bw	5
mzwid	0.015
minfrac	0.5
minsamp	1
**Annotation**	
feature.Annotation.CAMERA.annotate	isotopes
feature.Annotation.CAMERA.mzabs	0.015
feature.Annotation.CAMERA.ppm	5
featureAnnotation.CAMERA.sigma	6
featureAnnotation.CAMERA.perfwhm	0.6
featureAnnotation.CAMERA.maxcharge	3
featureAnnotation.CAMERA.maxiso	5
featureAnnotation.CAMERA.intensity	into
